# *Cbx3*/HP1γ deficiency confers enhanced tumor-killing capacity on CD8^+^ T cells

**DOI:** 10.1038/srep42888

**Published:** 2017-02-21

**Authors:** Michael Sun, Ngoc Ha, Duc-Hung Pham, Megan Frederick, Bandana Sharma, Chie Naruse, Masahide Asano, Matthew E. Pipkin, Rani E. George, To-Ha Thai

**Affiliations:** 1 Beth Israel Deaconess Medical Center, Harvard Medical School, Department of Pathology, Boston, MA 02215, USA; 2 Department of Neurobiology and Anatomy, Drexel University, College of Medicine, 2900 Queen Lane, Philadelphia, PA 19129, USA; 3 Division of Gastroenterology, Hepatology and Nutrition, Cincinnati Children’s Hospital Medical Center, Cincinnati, OH 45229, USA; 4 Department of Cancer Biology, The Scripps Research Institute, Jupiter, FL, 33458, USA; 5 Department of Pediatric Hematology/Oncology, Dana-Farber Cancer Institute and Boston Children’s Hospital, Boston, MA 02215, USA; 6 Institute of Laboratory Animals, Graduate School of Medicine, Kyoto University, Yoshida-Konoe-cho, Sakyo-ku, Kyoto, 606-8501, Japan; 7 Department of Pediatrics, Harvard Medical School, Boston, MA 02115, USA

## Abstract

*Cbx3*/HP1γ is a histone reader whose function in the immune system is not completely understood. Here, we demonstrate that in CD8^+^ T cells, *Cbx3*/HP1γ insufficiency leads to chromatin remodeling accompanied by enhanced *Prf1, Gzmb* and *Ifng* expression. In tumors obtained from *Cbx3*/HP1γ-insufficient mice or wild type mice treated with *Cbx3*/HP1γ-insufficient CD8^+^ T cells, there is an increase of CD8^+^ effector T cells expressing the stimulatory receptor *Klrk1*/NKG2D, a decrease in CD4^+^ CD25^+^ FOXP3^+^ regulatory T cells (Treg cells) as well as CD25^+^ CD4^+^ T cells expressing the inhibitory receptor CTLA4. Together these changes in the tumor immune environment may have mitigated tumor burden in *Cbx3*/HP1γ-insufficient mice or wild type mice treated with *Cbx3*/HP1γ-insufficient CD8^+^ T cells. These findings suggest that targeting *Cbx3*/HP1γ can represent a rational therapeutic approach to control growth of solid tumors.

The heterochromatin protein 1 (HP1) family of proteins are histone readers that associate with modified histones, and are involved in epigenetic regulations^1^,^2^. *Cbx3*/HP1γ interacts with the methyl groups of histone H3 at lysine 9 (H3K9Me3) and with methyl transferases as well as other proteins^3^,^4^,^5^,^6^. It has been shown to associate with both heterochromatin and euchromatin suggesting it may participate in transcriptional repression and activation, respectively^7^,^8^. Additionally, *Cbx3*/HP1γ controls gene expression through two intimately related processes: transcriptional elongation and co-transcriptional splicing of nascent RNA transcripts^9^,^10^,^11^,^12^. Homozygous germline deletion of *Cbx3*/HP1γ in mice causes embryonic lethality^13^,^14^. Previously we have shown that *Cbx3*/HP1γ haploinsufficiency is sufficient to regulate the high-affinity antibody response to protein antigens^15^. However, it has not been determined how *Cbx3*/HP1γ does so.

After encountering infected or malignant cells, naïve CD8^+^ T cells become activated and differentiate into effector cells to clear the infectious agent or control tumor growth^16^. Majority of effector cells die, but a small number will become memory cells. However, under persistent antigen exposure as in cancer or chronic infections, a subset of CD8^+^ effector T cells can enter an altered differentiation program known as T-cell exhaustion (T_EX_)^17^. CD8^+^ T_EX_ cells express unique transcription factors as well as those shared with effector/memory cells, among them are *Tbx21*/T-bet, *Eomesodermin (Eomes*) and *Prdm1*/Blimp-1. However, these factors interact with distinct partners in addition to being differentially expressed in the two populations^17^,^18^. More importantly, CD8^+^ T_EX_ cells can be reversed to become functional. Thus, identifying novel mechanisms that can reinvigorate exhausted CD8^+^ T cells would provide means to control tumor growth.

The ability of CD8^+^ effector T cells to kill targets requires the production of lytic molecules, perforin (encoded by *Pfr1*) and granzyme B (encoded by *Gzmb*), and the inflammatory cytokine interferon γ (INFγ, encoded by *Ifng*). Induction of *Pfr1, Gzmb* and *Ifng* is regulated in part by the transcription factor Runt-related 3 (Runx3)^19^,^20^.

Cytotoxic CD8^+^ T cells detect malignant-self either directly through interaction of the T-cell receptor (TCR) on CD8^+^ T cells with tumor antigens or indirectly in the form of so-called “danger signals” such as the retinoic acid early transcript-1 (RAE-1) and related ligands. Nonetheless, most activated cytotoxic CD8^+^ T cells (human and mouse) share the expression of the natural killer group 2, member D receptor (NKG2D) encoded by *Klrk1*, which upon binding to RAE-1 or related ligands, induced on tumor cells, stimulates effector responses that are independent of TCR^21^. For many tumors, tumor infiltrating *Klrk1*/NKG2D^+^ CD3^+^ CD8^+^ T cells or T cells expressing chimeric *Klrk1*/NKG2D receptor were shown to have promising anti-tumor efficacy^22^,^23^,^24^,^25^,^26^,^27^.

The elimination of CD4^+^ FOXP3^+^ Treg cells by T cells expressing the chimeric *Klrk1*/NKG2D receptor in the ovarian tumor microenvironment inhibited tumor growth and increased survival in mice^27^. Similarly, treatment of melanoma tumor-bearing mice with monoclonal antibody specific for CTLA4 led to the selective depletion of CD4^+^ Treg cells and a concomitant increase of CD8^+^ effector T cells within tumor lesions resulting in increased survival^28^,^29^. These results suggest that finding new means to manipulate the ratio of effector CD8^+^ T cells over CD4^+^ Treg cells is a viable strategy to control cancer.

Neuroblastoma (NB) is the most common extracranial solid tumor of childhood and the third most common cause of pediatric cancer death^30^. Despite the use of multimodal therapy, patients with high-risk NB have a poor prognosis due to high rate of relapse. Treatment with anti-GD2, a ganglioside expressed on a subset of NB tumors cells, is the current standard of care for high-risk NB^31^,^32^,^33^. However, this therapy causes severe pain requiring the use of continuous opiate infusions because GD2 is also expressed on pain fibers, and not all patients respond to this therapy^34^. Therefore, there is a need to explore novel approaches including immunotherapies to control high-risk NB.

Here we find that adoptive transfer of *Cbx3*/HP1γ-insufficient CD8^+^ effector T cells alone into wt tumor-bearing mice greatly limits tumor growth. Within the tumor microenvironment of *Cbx3*/HP1γ-insufficient mice or wt mice treated with *Cbx3*/HP1γ-insufficient CD8^+^ T cells, we detect an increase of *Klrk1*/NKG2D^+^ infiltrating CD8^+^ effector T cells, a concomitant decrease in CD4^+^ Treg cells. We propose that together these changes may have provided the anti-tumor immunity observed in mice.

## Results

### *Cbx3*/HP1γ-insufficient CD8^+^ T cells are endowed with enhanced effector capacity

**Figure 1:**
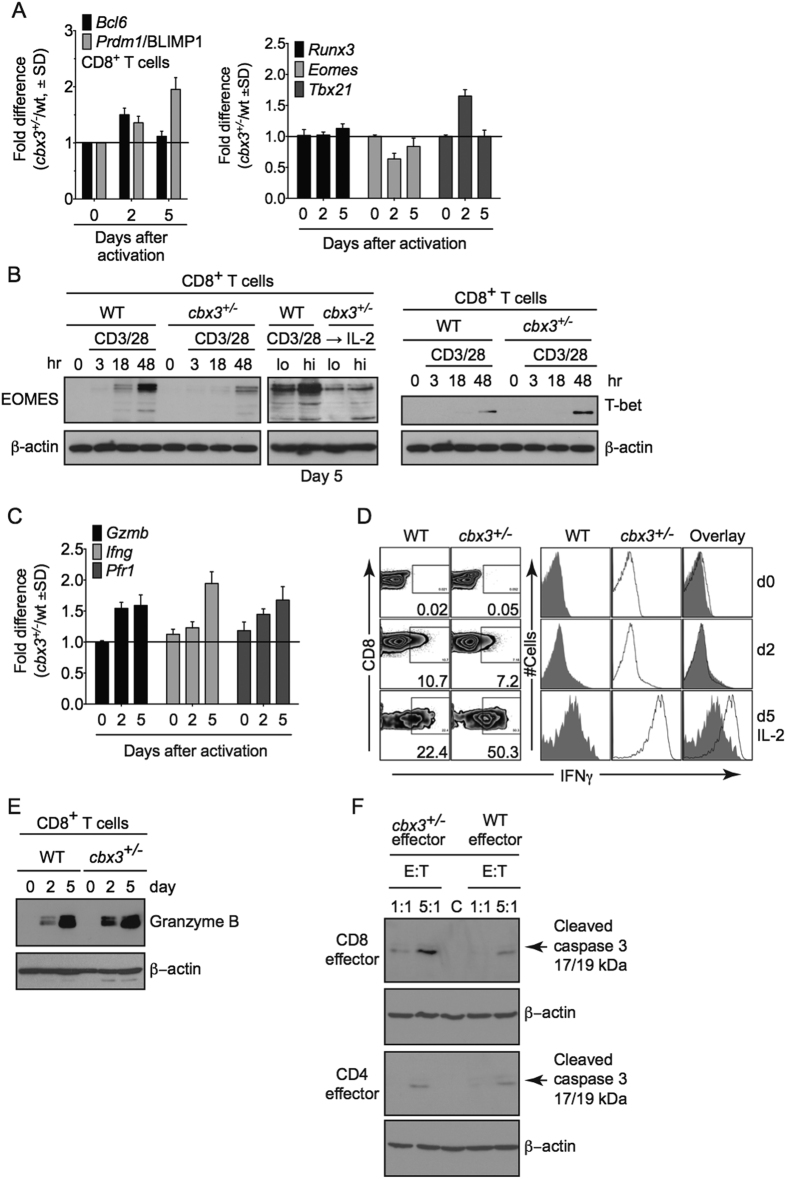
*Cbx3*/HP1γ-insufficient CD8^+^ effector T cells are endowed with enhanced killing capacity. (**A**) *Bcl6, Prdm1*/Blimp, *Runx3, Eomes* and *Tbx21* mRNA levels were assessed by RT-qPCR from *in vitro*-activated CD8^+^ T cells; *p* = *0.05* for *Eomes* day 5. (**B**) Eomes and T-bet proteins were detected by Western blots using total lysates from cells generated as in (**A**); *lo:* low rIL-2 concentration (10 U/ml), *hi*: high IL-2 (100 U/ml. (**C**) *Gzmb, Ifng* and *Prf1* mRNA levels were determined by RT-qPCR using cells as in (**A**). (**D**) The frequency of IFNγ^+^ population was determined using intracellular FACS with cells as in (**A**). Numbers in FACS plots represented percent cells. Histograms indicated IFNγ protein expression levels. (**E**) Granzyme B protein expression was detected by Western blots using cells as in (**A**). (**F**) Effector CD4^+^ and CD8^+^ T cells were co-cultured with target NB-9464 cells at a 1:1 or 5:1 effector to target ratio for 24 hrs. Apoptosis, indicated by the presence of cleaved caspase 3, was assessed with Western blots using total NB-9464 cell lysates from co-cultures. All results were representative of 3–5 independent experiments. For (**A** and **C**), results represented fold difference; unit 1 indicated no change (n = 10 of each genotype). Full-length Western blots are shown in Supplementary Information. For **A** and **C**, statistical analysis was performed with GrathPad unpaired student t-test.

Recently, we showed that in C57BL/6 mice, haploinsufficiency of *Cbx3*/HP1γ impairs the lymphoid-tissue germinal center (GC) reaction and high-affinity antibody response against a thymus (T)-dependent antigen (Ag) conjugated to chicken gamma globulin (NP-CGG)^15^. The effects were CD8^+^ -T-cell-intrinsic. Additionally, *Cbx3*/HP1γ protein was induced in CD8^+^ T cells after activation, and its level was greatly reduced in insufficient cells (Fig. S1A). These results prompted us to ask what CD8^+^ T-cell functions are controlled by *Cbx3*/HP1γ and by what mechanisms. Quantitative polymerase chain reaction (qPCR) and Western blot analyses demonstrated that on day 2 after activation, *Cbx3*/HP1γ-insufficient CD8^+^ T cells expressed more *Bcl6, Prdm1* and *Tbx21* compared to control cells (Fig. 1A,B). On day 5 of activation, *Prdm-1* expression was sustained at a high level in *Cbx3*/HP1γ-insufficient CD8^+^ T cells while that of *Runx3* and *Bcl6* was unaltered (Fig. 1A). By contrast, Eomes expression was repressed in *Cbx3*/HP1γ-insufficient CD8^+^ T cells on both days compared to control cells (Fig. 1A,B). Expression of *Prf1, Gzmb* and *Ifng* was upregulated in *Cbx3*/HP1γ-insufficient CD8^+^ T cells compared to controls (∼1.5 fold, 2 fold and 1.5 fold increase, respectively) (Fig. 1C–E). Correspondingly, a higher percentage of IFNγ^+^ cells was detected in *Cbx3*/HP1γ-insufficient cultures (50%) compared to wt controls (22%), and insufficient CD8^+^ T cells also produced a higher amount of IFNγ protein (Fig. 1D). A phenotypic survey of wt and insufficient activated CD8^+^ T cells showed they expressed similar levels of IL-2, CD25, CD127 (IL-7Rα) and CD132 (common γ chain or IL-2Rγ) (Fig. S1B–D). Similar frequency of effector (CD44^hi^CD62L^lo^) and central (CD44^hi^CD62L^hi^) memory CD8^+^ T cells was detected in wt and *Cbx3*/HP1γ-insufficient mice suggesting that activation did not appear to favor the expansion or reduction of either population in *Cbx3*/HP1γ-insufficient mice (Fig. S1E,F). To determine if the augmented production of perforin, granzyme B and IFNγ would endow *Cbx3*/HP1γ-insufficient CD8^+^ T cells to better induce apoptosis than wt cells, *in vitro*-activated CD8^+^ and CD4^+^ T cells were co-cultured with target neuroblastoma NB-9464 tumor cells^35^, and tumor-cell apoptosis assessed by measuring cleaved/activated caspase 3 (CC3) production. At a ratio of 5:1 (5 effector cells to 1 target cell) more CC3 was detected in tumor cells co-cultured with *Cbx3*/HP1γ-insufficient CD8^+^ effector T cells compared to control co-cultures (Fig. 1F). Wild type and insufficient CD4^+^ activated T cells induced similar CC3 level. These results show that after activation, *Cbx3*/HP1γ-insufficient CD8^+^ T cells could differentiate into effector cells armed with enhanced killing capacity to induce apoptosis in tumor cells.

### *Cbx3*/HP1γ deficiency limits tumor growth in mice

**Figure 2:**
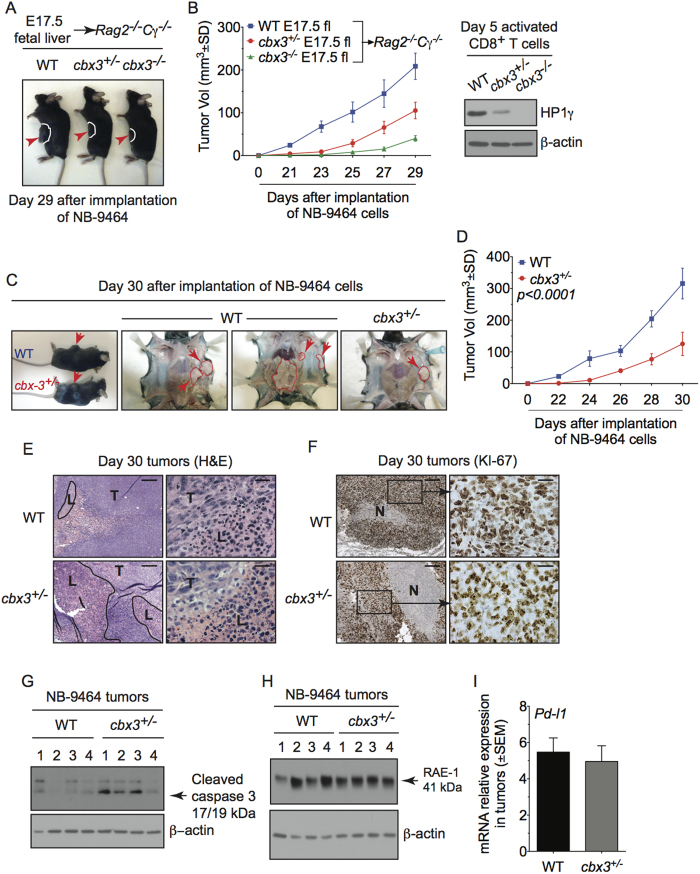
*Cbx3*/HP1γ deficiency limits tumor growth in mice. (**A**) Fetal liver reconstitution was performed with fetal liver cells from wt littermate, *Cbx3*/HP1γ^+/−^ and *Cbx3*/HP1γ^−/−^ day 17.5 embryos (E17.5) into *Rag2*^−/−^*cγ*^−/−^ recipient mice (n = 5-6 recipients for each genotype). Ten weeks after reconstitution, chimeric mice were implanted subcutaneously (sc) with NB-9464 tumor cells. *Red arrows* indicated tumors. (**B**) Tumor volume was measured on day 21 and every 2 days until day 29. Total *Cbx3*/HP1γ protein expression was assessed in day 5 *in vitro*-generated CD8^+^ effector T cells from chimeric mice. (**C**) Tumor implantation in wt and *Cbx3*/HP1γ-insufficient mice was performed as in (**A**). *Red arrows* and *red outlines* indicated tumor size and tumor in the peritoneal cavity of wt mice; n = 8 for each genotype. (**D**) Tumor volume was measured. (**E**) Tumor morphology and lymphocyte infiltration was assessed by hematoxylin and eosin (H&E) stain on paraffin sections of day 30 tumors. L = lymphocyte, T = tumor cells, *red outlines* indicated lymphocyte regions. (**F**) KI-67 was detected by immunohistochemistry on paraffin sections of day 30 tumors. Brown stain indicated KI-67 positivity, white unstained areas showed necrosis. For (**E** and **F**), images were shown as 100X (left, 10X ocular and 10X objective lens) and 400X (right, 10X ocular and 40X objective lens); 25 μm scale bar. (**G**) Day 30 tumors were excised and tumor cells were lysed. Cleaved caspase 3 was detected by Western blots. (**H**) RAE-1 protein expression was determined by Western blots in total lysates of day 30 tumors excised from wt and *Cbx3*/HP1γ-insufficient mice. (**I**) Relative *Pd-l1* mRNA expression was measured by RT-qPCR using day 30 tumor cells. All results were representative of 3 independent experiments with 4 different tumors. Full-length Western blots are shown in Supplementary Information. Statistical analysis was performed with the Graphpad Two-Way Anova (**B** and **D**) and student t-test (**I**).

CD8^+^ effector T cells can directly kill tumor cells by releasing perforin, granzyme B and IFNγ. We reasoned that *Cbx3*/HP1γ-insufficient CD8^+^ T cells armed with heightened killing capacity would be more efficient in controlling tumor growth compared to wt cells. To exclude the contribution of non-immune cells in tumor killing, we performed fetal liver reconstitution of *Rag2*^−/−^*cγ*^−/−^ mice, which have no T, B, NKT or NK cells. At week 10, mice reconstituted with wt littermate, *Cbx3*/HP1γ^+/−^ or *Cbx3*/HP1γ^−/−^ E17.5 fetal livers were implanted subcutaneously (sc) with NB-9464 tumor cells. Tumor growth was visible in mice reconstituted with wt fetal livers compared to those receiving *Cbx3*/HP1γ^+/−^ or *Cbx3*/HP1γ^−/−^ fetal livers (Fig. 2A). Concordantly, tumor volume was reduced in mice reconstituted with *Cbx3*/HP1γ^+/−^ or *Cbx3*/HP1γ^−/−^ fetal livers compared to those receiving wt fetal livers (Fig. 2B). As expected, CD8^+^ T cells from *Cbx3*/HP1γ^+/−^ and *Cbx3*/HP1γ^−/−^ fetal liver chimeras expressed significantly less and no *Cbx3*/HP1γ, respectively (Fig. 2B). To confirm that tumor volume reduction occurred in our *Cbx3*/HP1γ-insufficient mice as well, NB-9464 tumor cells were implanted in wt and *Cbx3*/HP1γ-insufficient mice. At day 30 after implantation, tumors from *Cbx3*/HP1γ-insufficient mice were smaller than those from wt mice, and ∼50% of tumor-bearing wt mice suffered tumor invasion into their peritoneal cavity, which was not observed in *Cbx3*/HP1γ-insufficient mice (Fig. 2C). Median volume of day 30 tumors from *Cbx3*/HP1γ-insufficient mice was 2.4 fold lower than that of wt mice (Fig. 2D). NB-9464 tumor-bearing *Cbx3*/HP1γ-insufficient mice survived longer than controls (Fig. S2). Tumors from *Cbx3*/HP1γ-insufficient mice exhibited extensive lymphocytic infiltration, decreased tumor-cell proliferation (less KI-67^+^ tumor cells) as well as large areas of necrosis, and increased tumor-cell apoptosis (higher levels of CC3) (Fig. 2E–G). By contrast, tumors excised from wt or *Cbx3*/HP1γ-insufficient mice expressed similar levels of the stimulatory RAE-1 and inhibitory programmed cell death ligand 1 (PD-L1) ligands, both of which were induced in tumor cells (Fig. 2H,I). These results demonstrate that *Cbx3*/HP1γ-insufficient mice are as efficient in controlling tumor growth as *Cbx3*/HP1γ^−/−^ fetal liver chimeras.

### *Cbx3*/HP1γ insufficiency increases CD8^+^ effector T cells and reduces CD4^+^ Treg cells in tumor microenvironment

**Figure 3:**
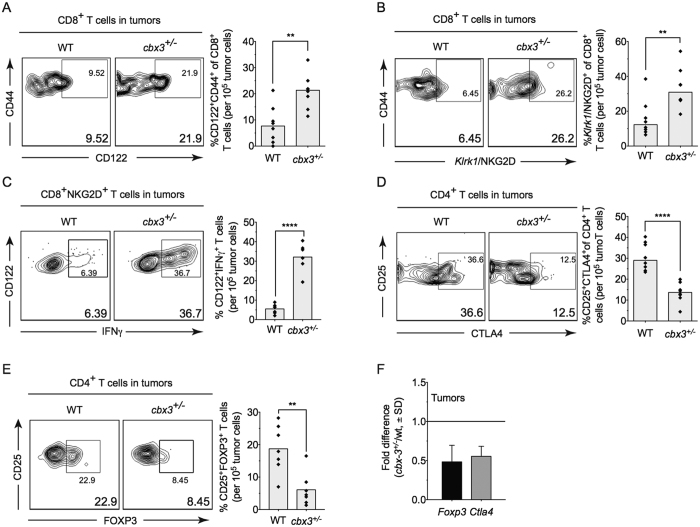
*Cbx3*/HP1γ insufficiency increases CD8^+^ effector T cells and reduces CD4+ Treg cells in tumor microenvironment. Flow cytometry was performed to identify tumor-infiltrating lymphocyte populations within day 30 tumors excised from wt and *Cbx3*/HP1γ-insufficient mice (n = 8). Graphs compiled FACS data. (**A**) NK1.1^−^CD122^+^ CD44^+^ CD3^+^ CD8^+^ T cells were gated from total CD8^+^ T-cell population in day 30 tumor-cell suspensions. Percent NK1.1^−^CD122^+^ CD44^+^ CD3^+^ CD8^+^ T cells were calculated from total CD8^+^ T cells, **p = *0.0023*. (**B**) NK1.1^−^NKG2D^+^ CD44^+^ CD3^+^ CD8^+^ T cells were gated from total CD8^+^ T-cell population in day 30 tumor-cell suspensions. Percent NKG2D^+^ CD44^+^ CD3^+^ CD8^+^ T cells were calculated from total CD8^+^ T cells, **p = *0.0067*. (**C**) CD122^+^ IFNγ^+^ cells were gated from CD8^+^ NKG2D^+^ T cells. Percent was calculated from total CD8^+^ NKG2D^+^ T cells, ****p < *0.0001*. (**D**) CD25^+^ CTLA4^+^ T cells were gated from total CD4^+^ T-cell population within day 30 tumors from wt or *Cbx3*/HP1γ^+/−^ mice. Percent CD25^+^ CTLA4^+^ T cells were calculated from total CD4^+^ T cell-population, ****p < *0.0001*. (**E**) CD25^+^ FOXP3^+^ cells were gated from CD4^+^ T cells. Percent was calculated from total CD4^+^ T-cell population, **p = *0.003*. (**F**) *Ctla4* and *Foxp3* mRNA expression was detected by RT-qPCR in day 30 tumors (4 tumors from each mouse strain). Results represented fold difference; unit 1 indicated no change. Each symbol represented an individual mouse; bars represented group median. Statistical analysis was performed with the GraphPad unpaired student t-test.

The successful control of tumor is in part dictated by the simultaneous activation of immune cells including CD8^+^ effector T cells and the elimination of immune suppressive elements in the tumor microenvironment. Within the microenvironment of NB-9464 tumors from *Cbx3*/HP1γ-insufficient mice, we detected an increased frequency of CD122^+^ CD44^+^ CD8^+^ (2.8 fold higher) T cells expressing the stimulatory receptor *Klrk1*/NKG2D (2.5-fold higher; Fig. 3A,B). The frequency of CD122^+^ IFNγ^+^ T cells among the CD8^+^ NKG2D^+^ population was 5 fold higher in tumors from *Cbx3*/HP1γ-insufficient mice compared to wt mice (Fig. 3C). The frequency of CD25^+^ CD4^+^ T cells expressing the inhibitory receptor CTLA4 was ∼2.6-fold lower in tumors from *Cbx3*/HP1γ-insufficient mice (Fig. 3D). Additionally, there was a ∼3-fold decrease in the frequency and number of CD25^+^ CD4^+^ FOXP3^+^ T cells (Fig. 3E and S3) as well as a reduction in *Foxp3* and *Ctla4* expression (Fig. 3F). It is plausible that NK and NKT cells, known to also participate in tumor clearance^21^,^36^, might have caused tumor growth reduction seen in our *Cbx3*/HP1γ-insufficient mice. However, we observed no differences in the frequency of NKT or *Klrk1*/NKG2D^+^ NKT cells in tumors from wt or *Cbx3*/HP1γ-insufficient mice (Fig. S4A,B). No discernable differences were observed in the frequency of tumor-infiltrating CTLA4^+^ CD8^+^ T cells from either wt or *Cbx3*/HP1γ-insufficient mice (Fig. S4C). Similar frequency of tumor-infiltrating ICOS^+^ CD4^+^ and ICOS^+^ CD8^+^ T cells was recovered from wt and *Cbx3*/HP1γ-insufficient mice (Fig. S4D). Tumor-infiltrating NK cells were not detected in tumors from either wt or *Cbx3*/HP1γ-insufficient mice (Fig. S4E). Tumors from wt and *Cbx3*/HP1γ-insufficient mice contained similar frequency of CD80^+^ and CD86^+^ cells (Fig. S4F). Relatively little differences were observed for other immune cells including B220^+^ B cells, Gr1^+^ and Mac-1^+^ myeloid cells in tumors from wt and *Cbx3*/HP1γ-insufficient mice (Fig. S4G,H). *Cbx3*/HP1γ-insufficient CD4^+^ T cells did not have the propensity to adopt a T_H_1 phenotype under non-polarizing condition (Fig. S4I). Moreover, *Cbx3*/HP1γ insufficiency did not affect the generation of natural CD4^+^ Treg cells in tumor bearing mice (Fig. S4J). Together, these results show that in tumors from *Cbx3*/HP1γ-insufficient mice there is an increase in CD8^+^ effector T cells and a decrease of CD4^+^ Treg cells as well as CD4^+^ CD25^+^ CTLA4^+^ T cells.

### Adoptive transfer of *Cbx3*/HP1γ-insufficient CD8^+^ effector T cells inhibits tumor growth in mice

**Figure 4:**
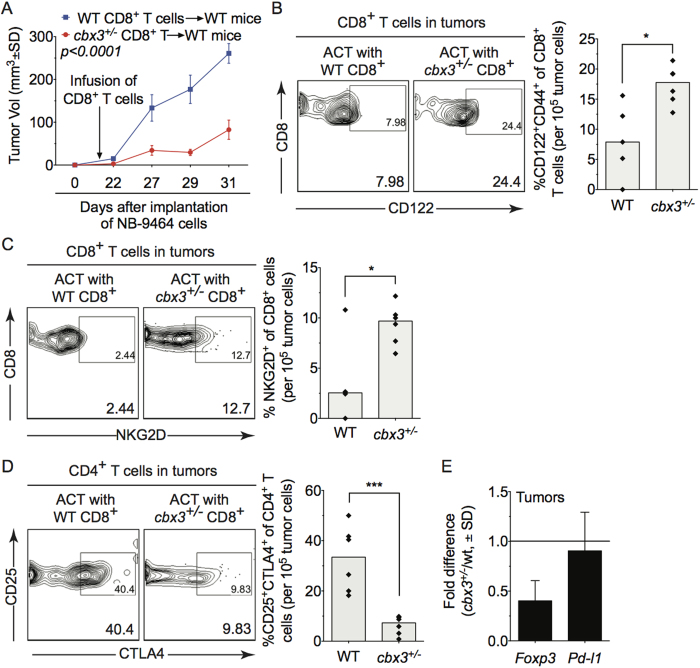
Adoptive transfer of *Cbx3*/HP1γ-insufficient CD8^+^ effector T cells inhibits tumor growth in mice. (**A**) On day 14 after induction, wt tumor-bearing mice were treated with wt or *Cbx3*/HP1γ-insufficient CD8^+^ effector T cells. Tumor volume was measured on day 22 after tumor induction (day 8 after treatment) until day 31 (n = 8 per treatment group). (**B**) NK1.1^−^CD122^+^ CD44^+^ CD3^+^ CD8^+^ T cells were gated from total CD8^+^ T-cell population in day 31 tumor-cell suspensions of treated mice. Percent NK1.1^−^CD122^+^ CD44^+^ CD3^+^ CD8^+^ T cells were calculated from total CD8^+^ T cells, *p = *0.0104*. (**C**) NK1.1^−^NKG2D^+^ CD44^+^ CD3^+^ CD8^+^ T cells were gated from total CD8^+^ T-cell population in day 31 tumor-cell suspensions of treated mice. Percent NKG2D^+^ CD44^+^ CD3^+^ CD8^+^ T cells were calculated from total CD8^+^ T cells, *p = *0.0373*. (**D**) CD25^+^ CTLA4^+^ T cells were gated from total CD4^+^ T-cell population within day 31 tumors of treated mice. Percent CD25^+^ CTLA4^+^ T cells were calculated from total CD4^+^ T cell-population, ***p = *0.0007*. (**E**) *Foxp3* and *PD-L1* mRNA expression was assessed by RT-qPCR in day 31 tumors obtained from treated mice (3 tumors from each treatment group). Fold difference was calculated, and the unit 1 indicated no change in expression levels. Each symbol represented an individual mouse; bars represented group median. Statistical analysis was performed with GraphPad unpaired student t-test.

To formally demonstrate that CD8^+^ T cells cause inhibition of tumor growth, adoptive transfer experiments were performed. NB-9464 tumor cells were implanted in wt mice, on day 12 or 14 after implantation, mice were treated with *in vitro*-activated wt or *Cbx3*/HP1γ-insufficient CD8^+^ T cells, and tumor growth was monitored until day 31. Treatment of wt tumor-bearing mice with *Cbx3*/HP1γ-insufficient CD8^+^ activated T cells alone was successful in inhibiting NB tumor growth (Fig. 4A). By contrast, tumor growth continued in mice treated with wt CD8^+^ effector T cells. Analyses of TILs recovered from treated mice revealed 2-fold more CD122^+^ CD44^+^ CD8^+^ effector T cells in tumors from mice treated with *Cbx3*/HP1γ-insufficient CD8^+^ effector T cells compared to those treated with wt cells (Fig. 4B). Similarly, a higher frequency (3.8-fold more) of *Klrk1*/NKG2D^+^ CD8^+^ T cells were present in tumors from mice treated with *Cbx3*/HP1γ-insufficient CD8^+^ T cells than those treated with wt cells (Fig. 4C). There was a significant reduction (4-fold less) in the frequency of CD4^+^ CD25^+^ CTLA4^+^ T cells and *Foxp3* expression in tumors obtained from mice treated with *Cbx3*/HP1γ-insufficient CD8^+^ T cells compared to those treated with wt cells (Fig. 4D,E). Expression of *Pd-l1* in tumors from mice treated with wt or *Cbx3*/HP1γ-insufficient CD8^+^ effector T cells remained similar (Fig. 4E). We observed no obvious differences in the frequency of other immune cells (Fig. S5A–C). Our results show that treatment with *Cbx3*/HP1γ-insufficient CD8^+^ effector T cells alone was sufficient to limit tumor growth in wt tumor-bearing mice.

### *Cbx3*/HP1γ occupies Prf1, Gzmb and Ifng loci and in part modulates histone H3K9me3 deposition

**Figure 5:**
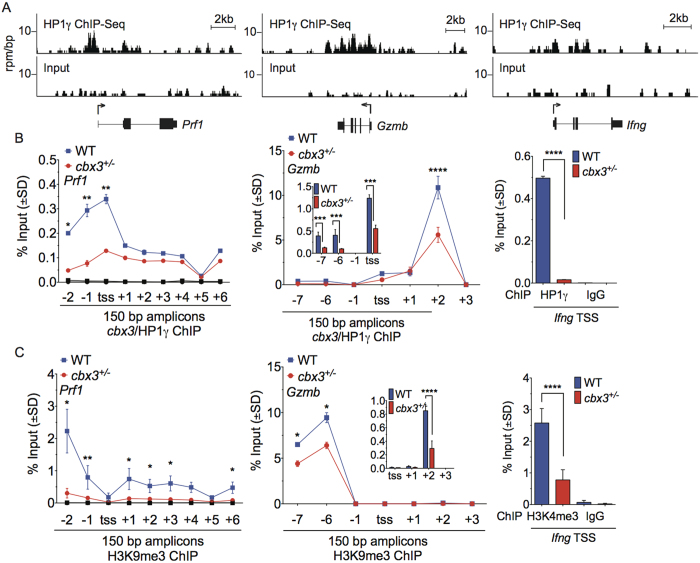
*Cbx3*/HP1γ occupies *Prf1, Gzmb* and *Ifng* loci and modulates histone H3K9me3 deposition. (**A**) ChIP-Seq was performed using chromatin from activated wt CD8^+^ T cells. Read-density tracks of *Cbx3*/HP1γ ChIP-Seq peaks across *Prf1, Gzmb* and *Ifng* were in black. The *y*-axis represented the number of reads per million mapped per 25-bp window; *x*-axis marked genomic locations. (**B**) The recruitment of *Cbx3*/HP1γ to *Prf1, Gzmb* and *Ifng* loci was confirmed by ChIP-qPCR, *p = 0.0181, **p = *0.0056* (−1kb) and *0.0062* (tss), ***p = 0.0001, ****p < *0.0001*. (**C**) Histone H3K9me3 deposition on *Prf1, Gzmb* and *Ifng* loci was assessed, *p = *0.0271*, **p = 0.0011, ****p < *0.0001*. All ChIP-qPCR results were representative of 4–5 independent ChIPs. TSS: transcription start site, numbers on *x*-axis indicated positions of primers (in kb) along *Prf1* and *Gzmb* loci, and 150 bp products were amplified using specific primers. Statistical analysis was performed with GraphPad unpaired student t-test and One-Way Anova.

It is not known if and how *Cbx3*/HP1γ controls CD8^+^ T-cell effector programming. To objectively address these questions, Chromatin Immunoprecipitation followed by deep Sequencing (ChIP-Seq) was performed using chromatin from *in vitro*-activated wt CD8^+^ T cells. *Cbx3*/HP1γ was distributed across the entire genome of activated mouse CD8^+^ T cells with less occupancy at 3′-UTRs and downstream regions compared to ChIP-Seq profile of an unrelated control antibody (Table S1 and Fig. S6). Among genes occupied by *Cbx3*/HP1γ were *Prf1, Gzmb* and *Ifng*, and ChIP-qPCR analyses validated *Cbx3*/HP1γ occupancy at these loci (Fig. 5A,B). *Cbx3*/HP1γ was recruited mainly to the −2, −1 kb 5′ upstream and transcription start site (TSS) regions of *Prf1*, while it associated predominantly with the −7, −6 kb 5′ upstream, TSS and +2 kb regions of *Gzmb. Cbx3*/HP1γ occupancy of *Ifng* locus could only be confirmed at the TSS site. By contrast, in insufficient CD8^+^ effector T cells, *Cbx3*/HP1γ recruitment to corresponding regions was significantly reduced (>2 fold reduction). Although *Bcl6, Prdm1, Tbx21* and *Eomes* expression in *Cbx3*/HP1γ-insufficient CD8^+^ T cells was modified, we observed no distinct *Cbx3*/HP1γ-bound peaks within these loci (Fig. S7A–D).

Because *Cbx3*/HP1γ has been shown to interact with H3K9me3, we asked if diminished *Cbx3*/HP1γ levels would lead to decreased histones H3K9me3 deposition at these loci. Indeed, less H3K9m3 was positioned at sites where there was reduced *Cbx3*/HP1γ occupancy, specifically at 5’ upstream regions and within the gene body of *Prf1* and *Gzmb* in *Cbx3*/HP1γ-insufficient CD8^+^ effector T cells compared to control cells (Fig. 5C). H3K9me3 deposition at *Prf1* and *Gzmb* TSS and +2 region of the latter was not readily detectable. In *Cbx3*/HP1γ-insufficient CD8^+^ effector T cells, positioning of H3K9m3 was decreased around *Ifng* TSS region, coinciding with reduced *Cbx3*/HP1γ occupancy. These results demonstrate that the degree of histones H3K9me3 deposition at *Prf1, Gzmb* and *Ifng* loci correlates, in part, with *Cbx3*/HP1γ occupancy.

### Runx3 occupancy and RNA Polymerase II (Pol II) recruitment/activation to Prf1, Ifng and Gzmb are regulated in part by *Cbx3*/HP1γ

We have previously shown that Runx3 together with Pol II in part regulate *Prf1, Ifng* and *Gzmb* expression in CD8^+^ effector T cells^19^,^20^. Here, ChIP-qPCR analyses showed that in *Cbx3*/HP1γ-insufficient CD8^+^ effector T cells, more Runx3 was deposited at sites where *Cbx3*/HP1γ and H3K9me3 occupancy was diminished (Fig. S8A). Additionally, more total Pol II was recruited to *Prf1* and *Ifng* TSS regions and at the −7, −6 kb 5′ upstream and +2 kb regions of *Gzmb* in *Cbx3*/HP1γ-insufficient CD8^+^ effector T cells compared to controls (Fig. S8B). Next we asked if *Cbx3*/HP1γ regulates Pol II initiation [Pol (S5)]^37^ and elongation [Pol II (S2)]^38^ phases as well. ChIP-qPCR results demonstrated that the presence of Pol II (S5) at *Prf1, Gzmb* and *Ifng* loci was augmented (Fig. S8C). Specifically, more Pol II (S5) was bound at the^+^ 1 and^+^ 4 regions of *Prf1* in *Cbx3*/HP1γ-insufficient CD8^+^ effector T cells. In *Cbx3*/HP1γ-insufficient CD8^+^ effector T cells, more Pol II (S5) occupied the −7 kb 5′ upstream and +2 kb regions of *Gzmb*, respectively; the binding pattern of Pol II (S5) and total Pol II was similar. There was also an increased in the level of Pol II (S5) assembled at the TSS region of *Ifng* in *Cbx3*/HP1γ-insufficient CD8^+^ effector T cells compared to control cells. There was a small increase in Pol II (S2) bound at all three loci in *Cbx3*/HP1γ-insufficient CD8^+^ effector T cells (Fig. S8D). These results show that more total and initiating/elongating Pol II are recruited to and assembled at *Prf1, Gzmb* and *Ifng* in *Cbx3*/HP1γ-insufficient CD8^+^ effector T cells.

## Discussion

Here, we reveal a previously unknown and essential role for *Cbx3*/HP1γ in the control of tumor immunity. We show that *Cbx3*/HP1γ insufficiency releases the effector capacity of CD8^+^ T cells to control tumor growth *in vivo*. Under *Cbx3*/HP1γ insufficient condition, chromatin remodeling occurs allowing increased Runx3 and active Pol II occupancy that in part could induce *Prf1, Gzmb* and *Ifng* expression. Together, these changes may have provided the anti-tumor immunity observed in our mouse model.

*Cbx3*/HP1γ function is diverse. It associates with both heterochromatin and euchromatin suggesting that it may participate in transcriptional repression and activation, respectively^7^,^8^. However, it is not understood what cellular conditions invoke the repressor or activator function to control gene expression. Our results demonstrate that in activated CD8^+^ T cells, *Cbx3*/HP1γ appears to act as a repressor of the activation-induced genes *Prf1, Gzmb* and *Infg*. We propose that in normal CD8^+^ T cells, *Cbx3*/HP1γ may interact with existing H3K9me3 to maintain an unfavorable chromatin structure that limits Runx3 invasion and Pol II recruitment/activation to prevent *Prf1, Gzmb* and *Infg* excessive expression. Because *Cbx3*/HP1γ occupancy does not completely correspond with H3K9me3 deposition at TSS of *Prf1* and *Gzmb* or +2 site of the latter, our data implies that *Cbx3*/HP1γ may interact with another protein(s) yet to be identified in CD8^+^ T cells. Additionally, we cannot rule out that *Cbx3*/HP1γ control unknown genes, which may affect *Prf1, Gzmb* and *Infg* expression.

The presence of CD8^+^ T_EX_ cells has been detected in a number of tumors nonetheless their effector capacity can be reinvigorated^17^. It is not clear if *Cbx3*/HP1γ-insufficient CD8^+^ tumor T cells are reinvigorated T_EX_ cells. The fact that more CD8^+^ T cells with effector characteristics are recovered from tumors of *Cbx3*/HP1γ-insufficient mice suggests that *Cbx3*/HP1γ insufficiency can reinvigorate the effector capacity of CD8^+^ T cells to control tumor growth. However, it is not yet known how *Cbx3*/HP1γ does so.

It is not yet understood why CD4^+^ Treg cells and CTLA4^+^CD25^+^CD4^+^ T cells are reduced. It is plausible that *Cbx3*/HP1γ-insufficient CD8^+^ effector T cells may alter the tumor microenvironment thus preventing CD4^+^ Treg infiltration^39^. Alternatively, *Cbx3*/HP1γ-insufficient CD8^+^ effector T cells may control CD4^+^ Treg numbers in tumors or *Cbx3*/HP1γ may directly control CD4^+^ Treg differentiation/function.

Several forms of immunotherapies are being used in the clinic with objective clinical responses. Here, we reveal an alternative novel strategy to boost tumor immunity. By reducing the levels of *Cbx3*/HP1γ we can increase CD8^+^ effector T cells at the same time eliminate CD4^+^ Treg immune suppression in the tumor microenvironment that may control tumor growth.

## Methods

### Mice and cell lines

*Cbx3*/HP1γ-deficient mice were generated, as described^13^. These mice were backcrossed to C57BL/6 for twelve generations. B6-*Rag2*^−/−^*Cγ*^−/−^ mice were purchased from Taconic. When needed, female 8–12 weeks old C57BL/6 mice were purchased from The Jackson Laboratory. All mice were maintained in specific pathogen-free conditions. All mouse protocols were approved by the BIDMC Institutional Animal Care and Use Committee. All experiments were performed in accordance with relevant guidelines and regulations. The NB-9464 neuroblastoma cell line was a kind gift from Dr. Crystal MacKall at Stanford University.

### Antibodies

All flow cytometry fluorochrome-conjugated antibodies were purchased from Biolegend, eBiosciences or BD Biosciences. All other antibodies were purchased as followed: anti-mouse total and phosphorylated *Cbx3*/HP1γ (Cell Signaling); anti-mouse RAE-1 pan-specific (R&D Systems); anti-mouse granzyme B (clone 16G6, eBiosciences); anti-mouse Eomes (Abcam); anti-mouse T-bet (clone 4B10, BioLegend); anti-mouse cleaved caspase 3 (Cell Signaling).

### Tumor induction

*Cbx3*/HP1γ^+/−^ and wild-type littermate mice were implanted subcutaneously with NB-9464 (1 × 10^6^/mouse) tumor cells in 100 μl PBS. NB-9464 tumor volume was measured and calculated (W × L × 0.4) on day 22 and every 2 days until day 30 or 36 depending on the size of the tumor, at which time mice were sacrificed for analysis.

### Fetal liver chimeras

At 17.5 days post coitum (d.p.c), pregnant female mice were euthanized and fetuses were retrieved. Genotyping of fetuses were performed. Fetal livers from fetuses of the correct genotype were dissected. Fetal liver cell suspension was prepared by passing through a 40 μm cell strainer and a 3-ml syringe in cold medium. Fetal liver cells were washed and resuspended at 1 × 10^7^/ml in cold PBS. 200 μl of fetal liver cell suspension was injected intravenously into 7 week-old *Rag2*^−/−^*Cγ*^−/−^ recipients. Ten weeks after reconstitution, chimeric mice were implanted subcutaneously with 1 × 10^6^ NB-9464 tumor cells in 100 μl PBS. Tumor volume was measured and calculated (W × L × 0.4) on day 21 and every 2 days until day 29, at which time mice were sacrificed for analysis. Control wt fetal livers were obtained from littermate fetuses.

### Adoptive T-cell therapy (ACT)

*In vitro*-generated CD8^+^ effector T cells were prepared as above. On day 0, female C57BL/6 mice were implanted subcutaneously with NB-9464 (1 × 10^6^/mouse) tumor cells in 100 μl PBS. On day 14 after tumor induction, mice were treated with 3 × 10^6^ CD8^+^ effector T cells via intravenous injection. NB-9464 tumor volume was measured and calculated (W × L × 0.4) on day 22 until day 31, at which time mice were sacrificed for analysis.

### Fluorescence-activated cell sorting or flow cytometry (FACS)

FACS was performed on the BD 5-laser LSR II. Analysis was performed with FlowJo software (Tree Star, Inc.). Tumor cells and tumor infiltrating lymphocytes were prepared from NB-9464 tumors 29 or 30 days after implantation. Tumors were harvested, minced, and cell suspension was filtered through a 70 μm cell strainer (Falcon). Cells were washed and stained for appropriate surface markers as indicated in figures. Intracellular IFNγ staining was performed according to manufacturer’s protocol (Affymetrix/eBioscience). Briefly, activated or intratumoral CD8^+^ T cells (1 × 10^6^) were treated with 1X cell stimulation cocktail with protein transport inhibitors (Affymetrix/eBioscience) in complete T-cell medium for 4 hrs at 37 °C. Cells were harvested and washed 2X with FACS buffer [PBS, 2% fetal bovine serum (FBS)]. Standard surface staining was performed. Cells were washed and incubated in fixation/permeabilization buffer (Affymetrix/eBioscience) for 30 minutes at room temperature (RT) or 18 hrs at 4 °C. Following incubation, cells were washed 2X with permeabilization buffer (Affymetrix/eBioscience) and stained for IFNγ using fluorochrome-antibodies. Cells were incubated for 30 minutes at RT. Cells were washed 2X with permeabilization buffer and resuspended in FACS buffer for analysis. Intracellular FOXP3 staining of intratumoral CD4^+^ CD25^+^ T cells was performed using similar protocol but without activation with stimulation cocktail.

### *In vitro* activation and differentiation of CD8^+^ T cells to generate effector cells

CD8^+^ CD44^−^ T cells were purified from spleen and peripheral lymph nodes using mouse CD8 FlowComp dynabeads according manufacturer’s protocol (Life Technologies/Thermo Fisher Scientific). T cells (1 × 10^6^/ml) were activated with plate-bound anti-CD3 (clone 145-2C11, 0.25 μg/ml, BioLegend) and anti-CD28 (clone 37.51, 0.5 μg/ml, BioLegend) in T-cell medium (DMEM, 10% FBS, P/S, non-essential amino acids, HEPES, L-glutamate and sodium pyruvate) at 37 °C in 10% CO_2_ for 2 days. Cells were removed from CD3/CD28 activation and recultured (5 × 10^5^/ml) in T-cell medium supplemented with IL-2 (10 U/ml or 100 U/ml) at 37 °C in 10% CO_2_. Cells were subcultured everyday. On day 5, cells were harvested and used for analyses or adoptive T-cell therapy.

### Co-cultures of effector cells with NB-9464 tumor cell line for apoptosis induction

Target NB-9464 tumor cells were plated in 12-well plates at a density of 5 × 10^5^ cells per well in 750 μl of RPMI with 10% fetal bovine serum (FBS). Activated CD8^+^ or CD4^+^ T cells were added. For ratio of 1:1 (effector:target), 5 × 10^5^ CD8^+^ or CD4^+^ T cells: 5 × 10^5^ NB-9464 cells were co-cultured in the same well; ratio 5:1, 2.5 × 10^6^ CD8^+^ or CD4^+^ T cells: 5 × 10^5^ NB-9464 cells. Plates were incubated at 37 °C in 5% CO_2_ for 24 hours. Wells were washed to remove non-adherent T cells. Adherent NB-9464 cells were collected and washed with 1 ml cold PBS.

### Histology and immunohistochemistry

Hematoxylin and eosin (H&E) staining and immunohistochemistry were performed using 4 μm thick formalin-fixed, paraffin-embedded tissue sections. Briefly, slides were soaked in xylene, passed through graded alcohols and put in distilled water. Slides were then pre-treated with 1.0-mM EDTA, pH 8.0 or 1.0 mM Citrate (Zymed) in a steam pressure cooker (Decloaking Chamber, BioCare Medical) as per manufacturer’s instructions, followed by washing in distilled water. All subsequent steps were performed at room temperature in a hydrated chamber. Slides were pre-treated with Peroxidase Block (DAKO) for 5 minutes to quench endogenous peroxidase activity, followed by Serum free Protein Block (DAKO) for 20 minutes. Rabbit monoclonal antibody against mouse KI-67 (clone D3B5, Cell Signaling Technology) was applied and slides were incubated overnight at 4 °C. Slides were washed 2X in 50 mM Tris-Cl, pH 7.4 then followed with goat anti-rabbit IgG-HRP (SouthernBiotech), and incubated for 15 minutes. After further washing, immunoperoxidase staining was developed using a DAB chromogen (DAKO) and counterstained with hematoxylin. Images were acquired with the Nikon Eclipse TE2000 and were captured with the Hamamatsu Orca ER digital CCD camera and analyzed with NIS elements 4.13 software. Images were shown as 100X (10X ocular and 10X objective lens) and 400X (10X ocular and 40X objective lens).

### Western blots

Cells (1 × 10^6^) cells were lysed with Radio-Immunoprecipitation Assay (RIPA) buffer (Boston BioProducts) containing protease inhibitor cocktail (Roche) on ice for 30 minutes. Cells were centrifuged at 14,000 rpm for 15 minutes at 4 °C. Protein concentration was determined by Bio-Rad Protein Assay Kit (BioRad). 10 μg of protein extracts were denatured at 95 °C for 10 minutes, separated by SDS-PAGE, and transferred onto PVDF membranes (EMD Millipore). Membranes were probed with antibodies. Proteins of interest were detected with HRP-conjugated secondary antibodies and visualized with the Pierce ECL Western blotting substrate (Thermo Fisher Scientific). Membranes were exposed with HyBlot CL Autoradiography films (Denville Scientific, Inc.), and developed with the Kodax X-OMAT 2000 Processor. Films were scanned with the Epson XP-610 scanner using black and white setting, and images were captured by Adobe Photoshop Elements Editor.

### Reverse transcription-quantitative polymerase chain reaction (RT-qPCR)

The Bio-Rad Hard-Shell PCR Plates (BioRad) were used. In each well, 2.5 μl of sample was added in triplicates along with 7.5 μl of the primer mixture, which consisted of 0.5 μl forward primer, 0.5 μl reverse primer, 1.7 μl PCR grade water, and 5 μl of Roche LightCycler 480 SYBR Green I Master (Roche). The plate was centrifuged at 300 RCF for 1 minute and read in the Bio-Rad CFX384 Real-Time System (BioRad). The qPCR settings were as follow: 95 °C for 10:00 minutes 95 °C for 0:10 minutes, 62 °C for 0:20 minutes, 72 °C for 0:20 minutes (Read Plate), GOTO step 2 for 39 times, 92 °C for 0:05 minutes, Melt Curve 65.0 °C to 97.0 °C at increment 0.5 °C for 1:00 minutes (Read Plate), 40 °C for 0:10 minutes.

### Chromatin immunoprecipitation (ChIP)

The chromatin immunoprecipitation (ChIP) was performed using the MAGnify ChIP System according to manufacturer’s protocol with minor modifications to optimize the assay (Life Technologies). Briefly, 2 × 10^7^ CD8^+^ T cells were used for each ChIP experiment. The volume of Dynabeads Protein A/G, DNA Purification Magnetic Beads, and all the buffers were decreased by half of the suggested amount. Cells were lysed with 500 μl of lysis buffer containing 1X protease inhibitors and incubated on ice for 20 minutes. Chromatin was sheared using a Sonic Dismembrator (Fisher Scientific Model 120) at 50% power, at 10 seconds per cycle, for 7 cycles. The sheared chromatin was added to the Antibody-Dynabeads complex and incubated overnight at 4 °C in a rotating shaker for maximum binding. The heat source used was the Thermal Cycler from Bio-Rad (model C1000). The DNA was eluted using 80 μl of elution buffer, and the purified DNA was immediately used for qPCR.

### ChIP followed by deep sequencing (ChIP-Seq)

#### Chromatin Immunoprecipitation

Mouse day-5-activated T cells were fixed with 1% formaldehyde for 15 min and quenched with 0.125 M glycine. Chromatin was isolated by the addition of lysis buffer and disruption with a Dounce homogenizer. Lysates were sonicated and the DNA sheared to an average length of 300–500 bp. Genomic DNA (Input) was prepared by treating aliquots of chromatin with RNase, proteinase K and heat for de-crosslinking, followed by ethanol precipitation. Pellets were resuspended and the resulting DNA was quantified on a NanoDrop spectrophotometer. Extrapolation to the original chromatin volume allowed quantitation of the total chromatin yield. An aliquot of chromatin (30 μg) was precleared with protein G agarose beads (Invitrogen). Genomic DNA regions of interest were isolated using *cbx3*^+/−^/HP1γ antibody (Cell Signaling). Complexes were washed, eluted from the beads with SDS buffer, and subjected to RNase and proteinase K treatment. Crosslinks were reversed by incubation overnight at 65 °C, and ChIP DNA was purified by phenol-chloroform extraction and ethanol precipitation.

#### ChIP Sequencing (Illumina)

Illumina sequencing libraries were prepared from the ChIP and Input DNAs by the standard consecutive enzymatic steps of end-polishing, dA-addition, and adaptor ligation. After a final PCR amplification step, the resulting DNA libraries were quantified and sequenced on HiSeq 2500. Sequences (50 nt reads, single end) were aligned to the mouse genome (mm10) using the BWA algorithm. Aligns were extended in silico at their 3′-ends to a length of 200 bp, which is the average genomic fragment length in the size-selected library, and assigned to 32-nt bins along the genome. The resulting histograms (genomic “signal maps”) were stored in BigWig files. Details of data analysis are shown in Supplementary Information.

## Additional Information

**How to cite this article**: Sun, M. *et al*. *Cbx3*/HP1γ deficiency confers enhanced tumor killing capacity on CD8^+^ T cells. *Sci. Rep.* **7**, 42888; doi: 10.1038/srep42888 (2017).

**Publisher's note:** Springer Nature remains neutral with regard to jurisdictional claims in published maps and institutional affiliations.

## Supplementary Material

Supplementary Information
