# Supplementary material for: Cbx3/HP1γ deficiency confers enhanced tumor-killing capacity on CD8+ T cells
Source: Sci Rep. 2017 Feb 21;7:42888. doi: 10.1038/srep42888 (PMC5318867; doi:10.1038/srep42888)
Supplement: Supplementary Information [file srep42888-s1.pdf]

SUPPLEMENTARY INFORMATION

***Cbx3*/HP1γ deficiency confers enhanced tumor-killing capacity on $CD8^+$ T cells**

Michael Sun, Ngoc Ha, Duc-Hung Pham, Megan Frederick, Bandana Sharma, Chie Naruse, Masahide Asano, Matthew E. Pipkin, Rani E. George and To-Ha Thai[¶]

[¶]Corresponding author: tthai@bidmc.harvard.edu

Supplementary Information

Full-length Western blots for Figure 1

Supplementary Information
Full-length Western blots for Fig. 1

B

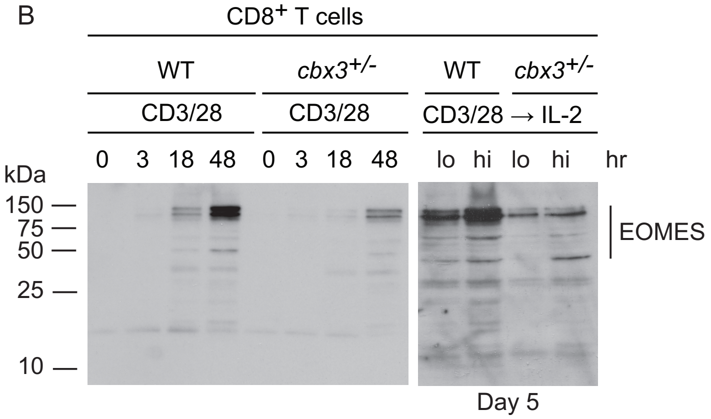

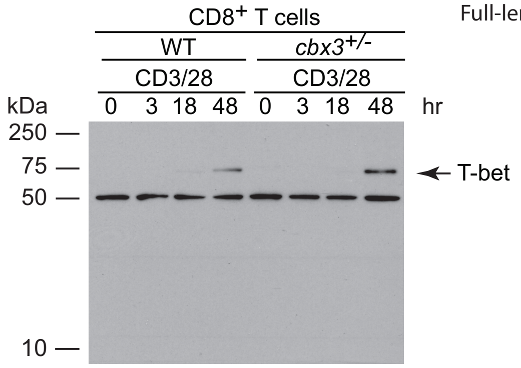

E

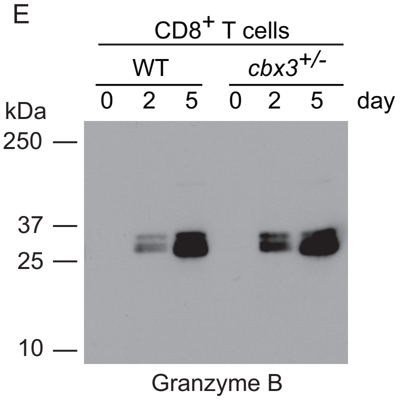

F

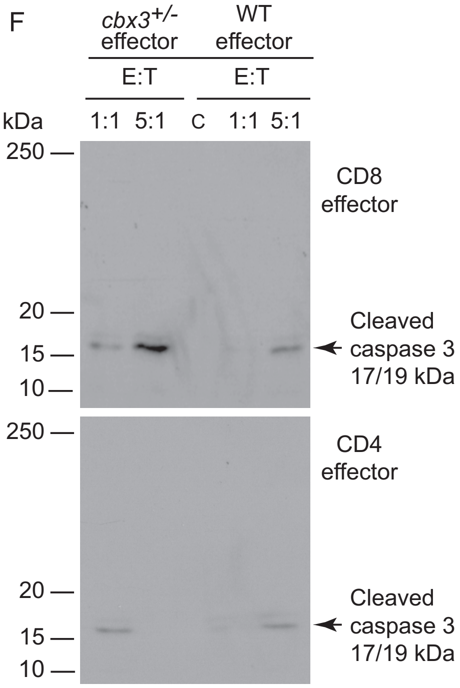

Full-length Western blots for Figure 2

Supplementary Information
Full-length Western blots for Fig. 2

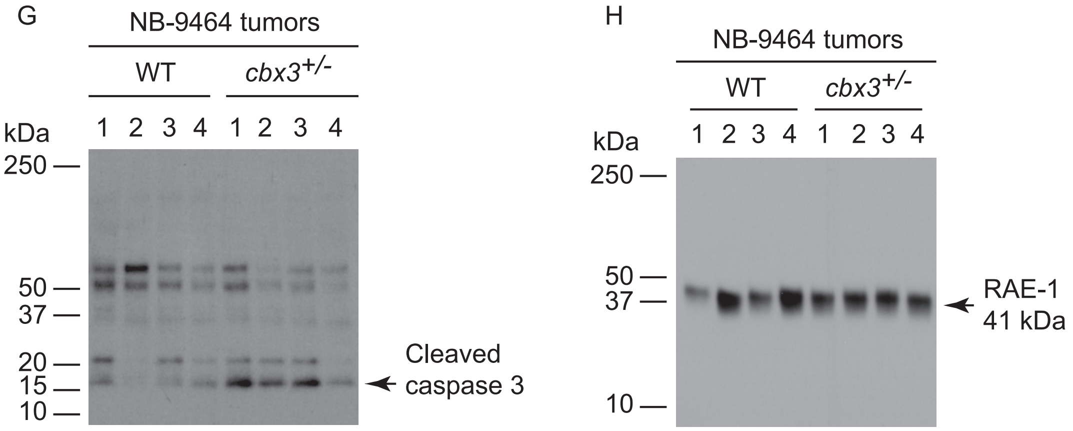

Supplementary Online Methods

ChIP-Seq

Data Analysis.

1. Sequence Analysis: The 50-nt sequence reads identified by the Sequencing Service (using
Illumina's Hi-Seq) are mapped to the genome using the BWA algorithm with default settings.
Alignment information for each read is stored in the BAM format. Only reads that pass Illumina's purity filter, align with no more than 2 mismatches, and map uniquely to the genome are used in the subsequent analysis. In addition, unless stated otherwise, duplicate reads ("PCR duplicates") are removed.

2. Determination of Fragment Density: Since the 5′-ends of the aligned reads (= "tags") represent the end of ChIP/IP-fragments, the tags are extended in silico (using Active Motif software) at their 3′-ends to a length of 150-250 bp, depending on the average fragment length in the size selected library (normally 150 bp). To identify the density of fragments (extended tags) along the genome, the genome is divided into 32-nt bins and the number of fragments in each bin is determined. This information ("signal map"; histogram of fragment densities) is stored in both a BAR file, which can be viewed in the Integrated Genome Browser (IGB), and a bigWig file, which can be uploaded to the UCSC Genome Browser (see Section V.). BAR/bigWig files also provide the peak metrics in the Active Motif analysis program described below.

3. "Peak Finding": The generic term "Interval" is used to describe genomic regions with local

enrichments in tag numbers. Intervals are defined by the chromosome number and a start and end

coordinate. The two main peak callers used at Active Motif are MACS and SICER. MACS is suitable to identify the binding sites of transcription factors that bind to discrete sites (often containing a consensus DNA sequence), while SICER is used to study proteins that bind to extended regions in the genome (such as histones or RNA polymerase II). Methylated sites or regions can be identified with both algorithms. Both methods look for significant enrichments in the ChIP/IP data file when compared to the Input data file (~ random background). Peaks can also be called by simple thresholding of the BAR files described in the previous paragraph.

4. Additional Analysis Steps:

a. Tag Normalization: In the default analysis, the tag number of all samples is reduced (by random sampling) to the number of tags present in the smallest sample.

b. Input File Analysis (optional): The signal map of the Input/IgG control file can be analyzed as an additional "sample". In this case, the strongest Input/IgG control peaks (which must represent false positives) are determined by simple thresholding of the BAR file, and only Input/IgG control peaks that overlap with Intervals in the ChIP/IP data are used in the analysis. By doing so, the output Active Region table (see Section IV.4, below) will show for each region the corresponding fragment density in the Input/IgG control sample, thus allowing for the identification of possible false positive ChIP/IP peaks.

5. Active Region Analysis: To compare peak metrics between 2 or more samples, overlapping

Intervals are grouped into "Active Regions", which are defined by the start coordinate of the most upstream Interval and the end coordinate of the most downstream Interval (= union of overlapping Intervals). In locations where only one sample has an Interval, this Interval defines the Active Region. The use of Active Regions is necessary because the locations and lengths of Intervals are rarely exactly the same when comparing different samples.

6. Annotations: After defining the Intervals and Active Regions, their genomic locations along with their proximities to gene annotations and other genomic features are determined and presented in Excel spreadsheets. In addition, average and peak (i.e. at "Summit") fragment densities within Intervals and Active Regions are compiled.

Data Visualization

1. UCSC Genome Browser

http://genome.ucsc.edu/cgi-bin/hgGateway/

2. IGB (Integrated Genome Browser)

http://bioviz.org/igb/download.html

3. IGV (Integrative Genomics Viewer)

http://www.broadinstitute.org/igv/

Primers for qPCR:

Bcl6:

(F) 5'-AGATGTGCCT CCATACTGCT-3'

(R) 5'-GCCTGGCAGCGATCACATTT-3'

Prdm1/Blimp1:

(F) 5'-AAAACGTGTGGGTACGACCT-3';

(R) 5’-GTAAGGATGCCTCGGCTTGA-3’

Runx3:

(F) 5’-AACGCTTCCGCTGTCATGAA-3’

(R) 5’-GTCGGGGTTCCCGGGGTCCA-3’

Eomes:

(F) 5’-CCTATGGCTCAAATTCCACC-3’

(R) 5’-GTTAGGAGATTCTGGGTGAA-3’

Tbx21:

(F) 5’-CCACTGGATG CGCCAGGAAG-3’

(R) 5’- CACTGCAATGAACTGGGTCT-3’

Prf1:

(F) 5’-TCCAAGGTAGCCAATTTTGC-3’

(R) 5’-GAGGAGATGAGCCTGTGGTA -3’

Gzmb:

(F) 5’-ACTGCTGCTCACTGTGAAGG-3’

(R) 5’- CACAGCTCTAGTCCTCTTGG -3’

Ifng:

(F) 5’-GCACAGTCATTGAAAGCCTA-3’

(R) 5’-GAAAGAGATAATCTGGCTCT-3’

Foxp3:

(F) 5’-CAAGCAGATCATCTCCTGGA-3’

(R) 5’-AAGAGCTCTTGTCCATTGAG-3’

Pd-l1:

(F) 5’-GGACTACAAGCGAATCACGC -3’

(R) 5’-CACTGTTTGTCCAGATTACC -3’

Cbx3/HP1γ:

(F) 5’-GGTGAACTCTTCAAGTCTCCG-3’

(R) 5’-TTATTGTGCT TCATCTTCAG GACAAG-3’

Il-2:

(F) 5’-GTACAGCATGCAGCTCGCATCC-3’

(R) 5’-AGCATCCTGGGGAGTTTCAG-3’

Il-2rα:

(F) 5’-TGCAAGAGAG GTTTCCGAAG-3’

(R) 5’-TGTTTCCAAGGAGGTGGCTC-3’

Icos:

(F) 5’-GCTCCCAGGGAAGCTATTACTTCTGC-3’

(R) 5’-CCCTACGGGTAGCCAGAGCTTCAGC-3’

Klrk1/NKG2D:

(F) 5’-CGAGTCCTTGCTATAGCCTTGG-3’

(R) 5’-GGTAACAGTTGTTTCTGTGA-3’

<u>Primers for ChIP-qPCR:</u>

Prf1:

-2kb (F) 5’-CTATCTTCAGGCACACCAAAGAG-3’

-2kb (R) 5’-AAAATAAGAATAAGTTGGGCTGGAG-3’

-1kb (F) 5’-AAAAGCTGTTAAAAGTGTGTTTCTGA-3’

-1kb (R) 5’-AAAGCTCAGAGACATTCAGTCCTTA-3’

TSS (F) 5’-CAGGGCAGGAAGTAGTAATGATATG-3’

TSS (R) 5’-CTTCCTCCTCCTTACCTGAAGTC-3’

+1kb (F) 5’-GACAGAGAGGAGATAGAGGGAGATT-3’

+1kb (R) 5’-CTCAGGTTTTCTGTCTTGACATTC-3’

+2kb (F) 5’-TTAAATCCCTCTGGATTTCTCTGTA-3’

+2kb (R) 5’-TAGTCATAAACTCTGGAAAGCTGTG-3’

+3kb (F) 5’-GAGAGCTTATTTCAGTCACATTTCC-3’

+3kb (R) 5’-TGTCTAACGATAGAGAAGGTTAGCTG-3’

+4kb (F) 5’-CTCTATGCATGAGCACTTACATCC-3’

+4kb (R) 5’-AGGTTTCCTGTCTCTGTCCCTAC-3’

+5kb (F) 5’-CTTACCACAGGCTCATCTCCTC-3’

+5kb (R) 5’-TTCAGGCAGTCTCCTACCTCAT-3’

+6kb (F) 5’-GAAATTCTCCTACCATGCCAAGT-3’

+6kb (R) 5’-TCTCAGGCATGTTATTGTTGTTATT-3’

Gzmb:

-7kb (F) 5’-CTCCTTGGGTACTTTCTCTAGC-3’

-7kb (R) 5’-TTGCATACACTAGCAAGGTTTT-3’

-6kb (F) 5’-TATTCTGACTGGTGTGAGGTGG-3’

-6kb (R) 5’-TAAAAAATGGGCTCAGAGCTAA-3’

TSS (F) 5’-ACTCTGATACCATAGGCTACAAACC-3’

TSS (R) 5’-TGATGACGTCTTCTGAGTACTTGTG-3’

+1kb (F) 5’-GTGCCCACAAAATGATTGGCTT-3’

+1kb (R) 5’-CAAAGTCCTCTCGAATAAGGAA-3’

+2kb (F) 5’-AGATATGTGCGGGGGACCCAAA-3’

+2kb (R) 5’-AAAGATCACAGCCAGTGGGTAA-3’

+3kb (F) 5’-TGACATCTTCCTATGGAAGTTT-3’

+3kb (R) 5’-CAACTGTATGAAAGTTCTGTAG-3’

Ifng:

TSS (F) 5’-ACTCTAACATGCCACAAAACCATAG-3’

TSS (R) 5’-CTTCCAGTTTTATACCTGATCGAAG-3’

***In vitro* activation of CD4$^+$ T cells for intracellular detection of IFNγ and IL-4.** Naïve CD4$^+$ T cells were purified from spleen and peripheral lymph nodes using mouse CD4 Dynabeads according to manufacturer’s protocol (Life Technologies/Thermo Fisher Scientific) followed by depletion of CD25$^+$ cells using MACS beads (Miltenyi Biotec). T cells (1 x 10^6/ml) were activated with plate-bound anti-CD3 (clone 145-2C11, 0.25 μg/ml, BioLegend) and anti-CD28 (clone 37.51, 0.5 μg/ml, BioLegend) in T-cell medium (DMEM, 10% FBS, P/S, non-essential amino acids, HEPES, L-glutamate and sodium pyruvate) containing 20 U/ml rhuIL-2 at 37°C in 10% CO_2. On day 2 after activation, cells were removed and expanded into new wells containing 20 U/ml rhuIL-2. On day 5, IFNγ and IL-4 intracellular staining was performed as described in Material and Methods.

SI Figure Legends

Fig. S1. *Cbx3*/HP1γ does not regulate IL-2, CD25, CD132 and CD127 expression or the generation of effector and central memory CD8$^+$ T cells. For all *in vitro* experiments, Dynal beads followed by MACS purified spleen and peripheral lymph node CD44$^-$CD8$^+$ T cells were activated with plate-bound CD3/CD28 antibodies for 2 days, then removed and cultured in 10 IU/ml rIL-2 from days 3-5. (A) Western blot was performed to detect

Cbx3/HP1γ protein expression in $CD8^+$ T cells. (B) RT-qPCR analysis was performed to determine mRNA levels of *Il2* and *Il2rα* in $CD8^+$ T cells activated and IL-2 conditioned on days 3-5. Results represented fold change, 1 indicated no change between wt littermate and *Cbx3*/HP1γ-insufficient mice; statistical analysis was performed with GraphPad student t-test. (C-F) Flow cytometry was performed to assess the expression levels of surface CD25 (C), CD127 (IL-7Rα) and CD132 (common γ chain or IL-2Rγ) (D), $CD44^+$ and $CD62L^+$ on day 0 cells (E), CD62L and CD44 on activated cells (F). Results were representative of 5 experiments (n=10 per genotype).

Fig. S2. Tumor-bearing *Cbx3*/HP1γ-insufficient mice survive longer. Mice were implanted sc with NB-9464 tumor cells and survival was monitored until day 36. Survival was analyzed using the log-rank (Mantel-Cox) test (n=8).

Fig. S3. The number of $CD25^+FOXP3^+CD4^+$ T cells was decreased in tumors from *Cbx3*/HP1γ-insufficient mice. Flow cytometry was performed to identify intratumoral $CD4^+$ Treg cells within day 30 tumors excised from wt and *Cbx3*/HP1γ-insufficient mice (n=8 per group). Cells were gated on CD4 then CD25 and FOXP3. Results depicted absolute numbers; statistical analysis was performed with GraphPad student t-test.

Fig. S4. The composition of other immune populations is not altered in *Cbx3*/HP1γ-insufficient mice. Flow cytometry was performed to identify tumor-infiltrating lymphocyte populations within day 30 tumors excised from wt or *Cbx3*/HP1γ-insufficient mice (n=8). (A) $NK1.1^+CD8^-$ T cells were gated from total $CD8^-$ cell population in day 30 tumor cell suspensions. Percent $NK1.1^+CD8^-$ T cells were calculated from total $CD8^-$ T cells. (B) $NK1.1^+NKG2^+CD8^-$ T cells were gated from total $NK1.1^+CD8^-$ T-cell population in day 30 tumor-cell suspensions. Percent $NK1.1^+NKG2^+CD8^-$ T cells were

calculated from total NK1.1$^+$CD8$^-$ T cells. (C) CD8$^+$CTLA4$^+$ T cells were gated from total CD8$^+$ T-cell population within day 30 tumors from wt or *Cbx3*/HP1γ$^{+/-}$ mice. Percent CD8$^+$CTLA4$^+$ T cells were calculated from total CD8$^+$ T cell-population. (D) CD8$^+$ICOS$^+$ and CD4$^+$ICOS$^+$ T cells in day 30 tumors were gated from total CD8$^+$ and CD4$^+$ T-cell populations, respectively. Percent CD8$^+$ICOS$^+$ and CD4$^+$ICOS$^+$ T cells were calculated from total CD8$^+$ and CD4$^+$ T cells, respectively. (E) NK cells (CD3$^-$DX5$^+$) cells were gated from total CD3$^-$ tumor cells. (F) CD4$^-$CD8$^-$CD80$^+$ and CD4$^-$CD8$^-$CD86$^+$ cells were gated from total CD4$^-$CD8$^-$ tumor cells. Percent CD4$^-$CD8$^-$CD80$^+$ and CD4$^-$CD8$^-$CD86$^+$ cells were calculated from total CD4$^-$CD8$^-$ tumor cells. (G) B220$^+$ B cells were gated from total CD4$^-$CD8$^-$ tumor cells. Percent B220$^+$ B cells were calculated from CD4$^-$CD8$^-$ tumor cells. (H) Gr-1$^+$ and Mac-1$^+$ cells were gated from total CD4$^-$CD8$^-$ tumor cells. Percent Gr-1$^+$ and Mac-1$^+$ cells were calculated from total CD4$^-$CD8$^-$ tumor cells. Each symbol represented an individual mouse; bars represented group median. (I) Intracellular IFNγ and IL-4 expression was assessed in naïve CD4$^+$ T cells that have been cultured under non-polarizing condition (anti-CD3/CD28, 20 U/ml IL-2) for 4 days; results were representative of 2 experiments. (J) The frequency of natural CD4$^+$ Treg cells was determined in the thymus and spleen of mice bearing day 30 tumors. Statistical analysis was performed with GraphPad student t-test.

Fig. S5. The frequency of other immune cells remains unaltered in tumors treated with *Cbx3*/HP1γ-insufficient CD8$^+$ effector T cells. (A) B220$^+$ B cells were gated from total CD4$^-$CD8$^-$ NB-9464 tumor cells. Percent B220$^+$ B cells were calculated from CD4$^-$CD8$^-$ tumor cells. (B,C) Gr-1$^+$ and Mac-1$^+$ cells were gated from total CD4$^-$CD8$^-$ NB-9464 tumor cells. Percent Gr-1$^+$ and Mac-1$^+$ cells were calculated from total CD4$^-$CD8$^-$ tumor

cells. Each symbol represented an individual mouse; bars represented group median. Statistical analysis was performed with GraphPad unpaired student t-test.

Fig. S6. *Cbx3*/HP1γ is distributed across the entire genome of mouse CD8$^+$ effector T cells. All Chromatin Immunoprecipitation followed by deep Sequencing (ChIP-Seq) experiments were performed with day 5 *in vitro*-activated wt CD8$^+$ T cells as in figure 1. *Pie chart* showed the distribution of *Cbx3*/HP1γ-bound regions across the genome of wt CD8$^+$ effector T cells. HP1γ: ChIP with anti- HP1γ; non-HP1γ: chIP with irrelevant antibody.

Fig. S7. *Cbx3*/HP1γ is not recruited to *Bcl6*, *Prdm1*, *Tbx21*, or *Eomes* locus. All Chromatin Immunoprecipitation followed by deep Sequencing (ChIP-Seq) experiments were performed with day 5 *in vitro*-activated wt CD8$^+$ T cells as in figure 1. (A) Read-density tracks of *Cbx3*/HP1γ ChIP-Seq peaks across *Bcl6*, (B) *Prdm1*/Blimp1, (C) *Tbx21*/Tbet and (D) *Eomes* were in black. The *y*-axis represented the number of reads per million mapped per 25-bp window; *x*-axis marked genomic locations.

Fig. S8. Runx3 occupancy and RNA Polymerase II (Pol II) recruitment/activation to *Prf1*, *Ifng* and *Gzmb* are regulated in part by *Cbx3*/HP1γ. Chromatin was prepared as in Fig. 5. (A) Runx3 occupancy at *Prf1*, *Gzmb* and *Ifng* loci was assessed by ChIP-qPCR, *$p=0.0107$ and 0.0258, **$p=0.0018$, ****$p<0.0001$. (B) The recruitment of total Pol II was determined, **$p=0.0017$, ***$p=0.0002$, ****$p<0.0001$. (C) The density of initiating Pol II (S5) was assessed, *$p=0.01$, **$p=0.0011$, ***$p=0.0002$. (D) The assembly of elongating Pol II (S2) was detected, *$p=0.0271$, **$p=0.0015$. All ChIP-qPCR results were representative of 3-4 independent ChIPs. Numbers on *x*-axis indicated positions of primers (in kb) along *Prf1* and *Gzmb* loci, and 150 bp products were amplified using

specific primers. Statistical analysis was performed with GraphPad unpaired student t test and One-Way Anova.

Fig. S1

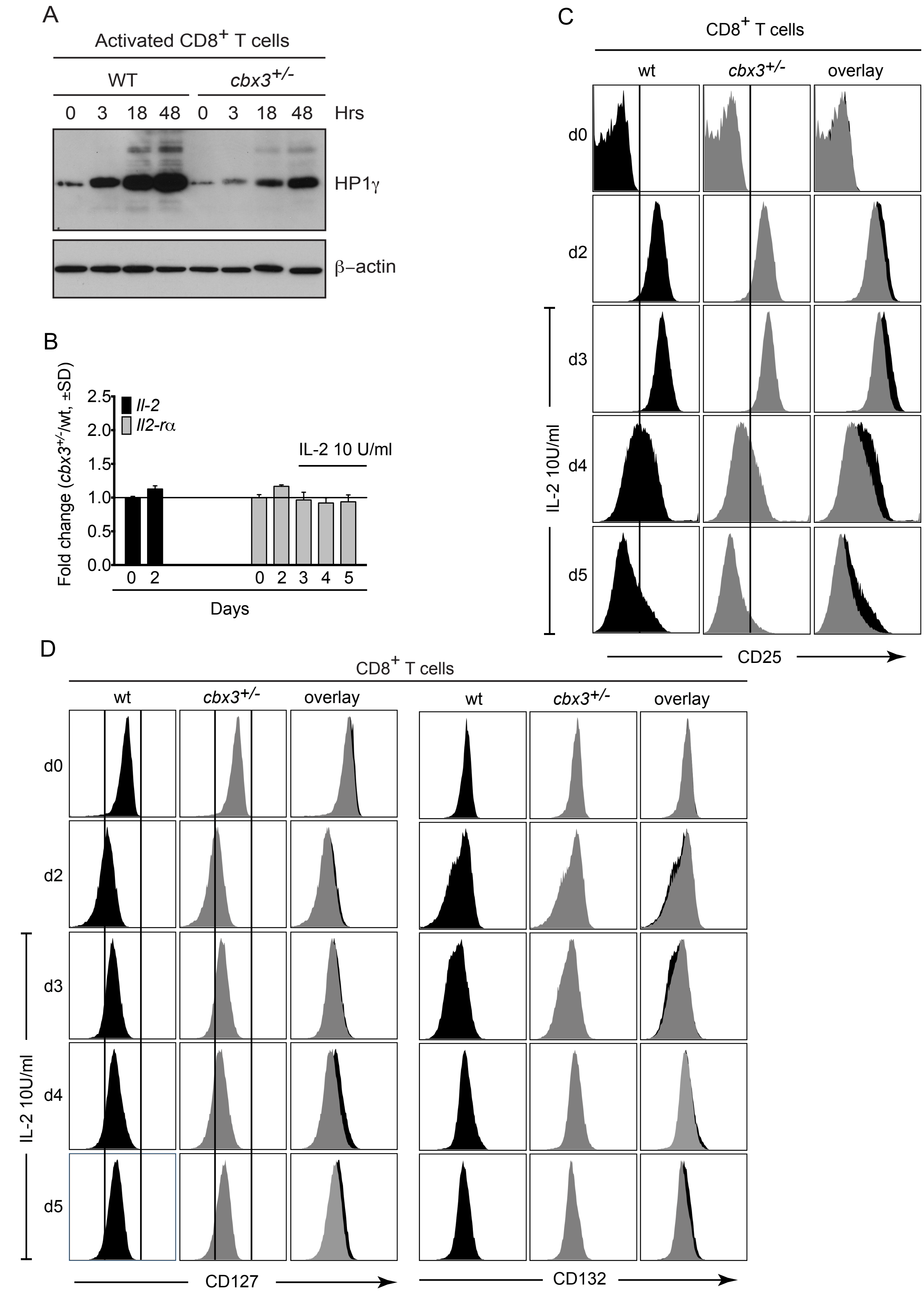

A
Activated CD8+ T cells
WT
cbx3+/-
0 3 18 48 0 3 18 48 Hrs
HP1γ
β-actin
B
Fold change (cbx3+/-/wt, ±SD)
Il-2
Il2-rα
IL-2 10 U/ml
2.5
2.0
1.5
1.0
0.5
0.0
0 2
0 2 3 4 5
Days
C
CD8+ T cells
wt
cbx3+/-
overlay
d0
d2
d3
d4
d5
IL-2 10U/ml
CD25
D
CD8+ T cells
wt
cbx3+/-
overlay
wt
cbx3+/-
overlay
d0
d2
d3
d4
d5
IL-2 10U/ml
CD127
CD132

E

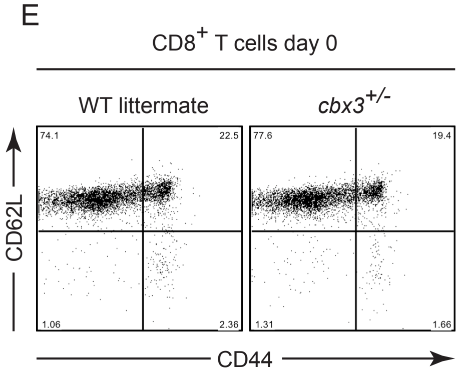
CD8+ T cells day 0
WT littermate
cbx3+/-
CD62L
CD44
74.1
22.5
1.06
2.36
77.6
19.4
1.31
1.66

F

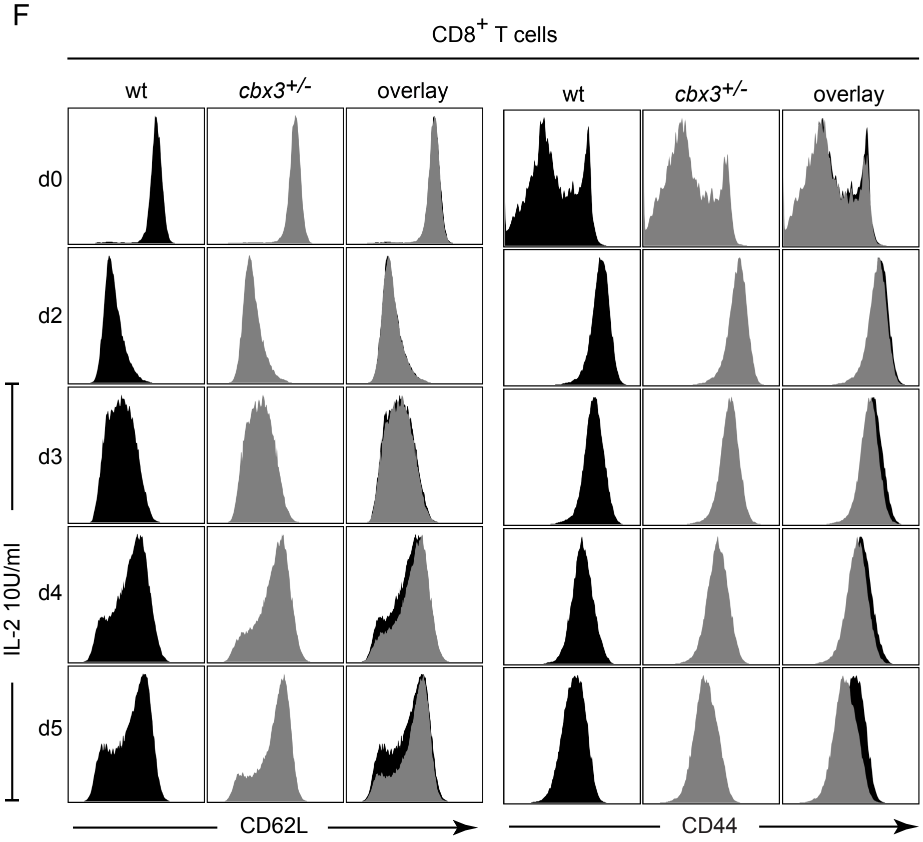
CD8+ T cells
wt
cbx3+/-
overlay
d0
d2
d3
d4
d5
IL-2 10U/ml
CD62L
CD44

Fig. S2

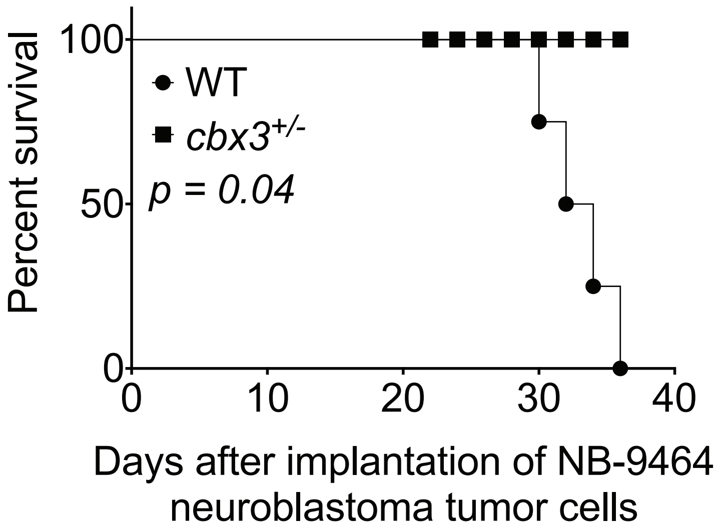

Percent survival
100
50
0
WT
cbx3+/-
p = 0.04
0
10
20
30
40
Days after implantation of NB-9464 neuroblastoma tumor cells

Fig. S3

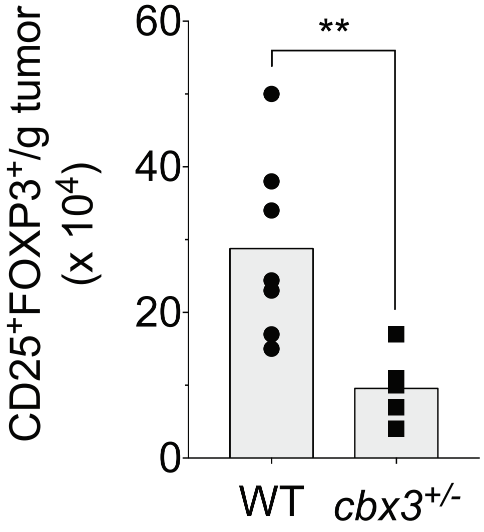

Fig. S4

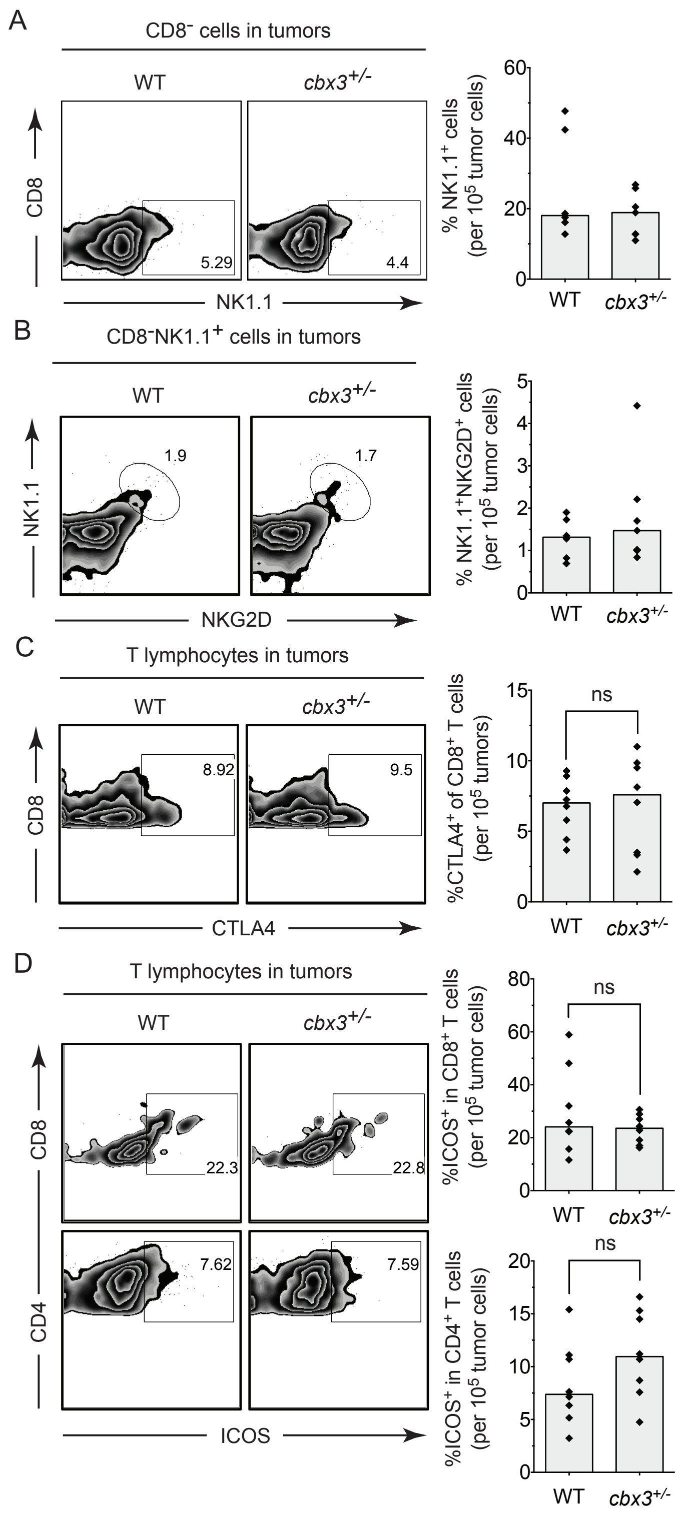
A
CD8⁻ cells in tumors
WT
cbx3+/-
CD8
NK1.1
5.29
4.4
% NK1.1⁺ cells (per 10⁵ tumor cells)
60
40
20
0
WT
cbx3+/-
B
CD8⁻NK1.1⁺ cells in tumors
WT
cbx3+/-
NK1.1
NKG2D
1.9
1.7
% NK1.1⁺NKG2D⁺ cells (per 10⁵ tumor cells)
5
4
3
2
1
0
WT
cbx3+/-
C
T lymphocytes in tumors
WT
cbx3+/-
CD8
CTLA4
8.92
9.5
%CTLA4⁺ of CD8⁺ T cells (per 10⁵ tumors)
15
10
5
0
ns
WT
cbx3+/-
D
T lymphocytes in tumors
WT
cbx3+/-
CD8
CD4
ICOS
22.3
22.8
7.62
7.59
%ICOS⁺ in CD8⁺ T cells (per 10⁵ tumor cells)
80
60
40
20
0
ns
WT
cbx3+/-
%ICOS⁺ in CD4⁺ T cells (per 10⁵ tumor cells)
20
15
10
5
0
ns
WT
cbx3+/-

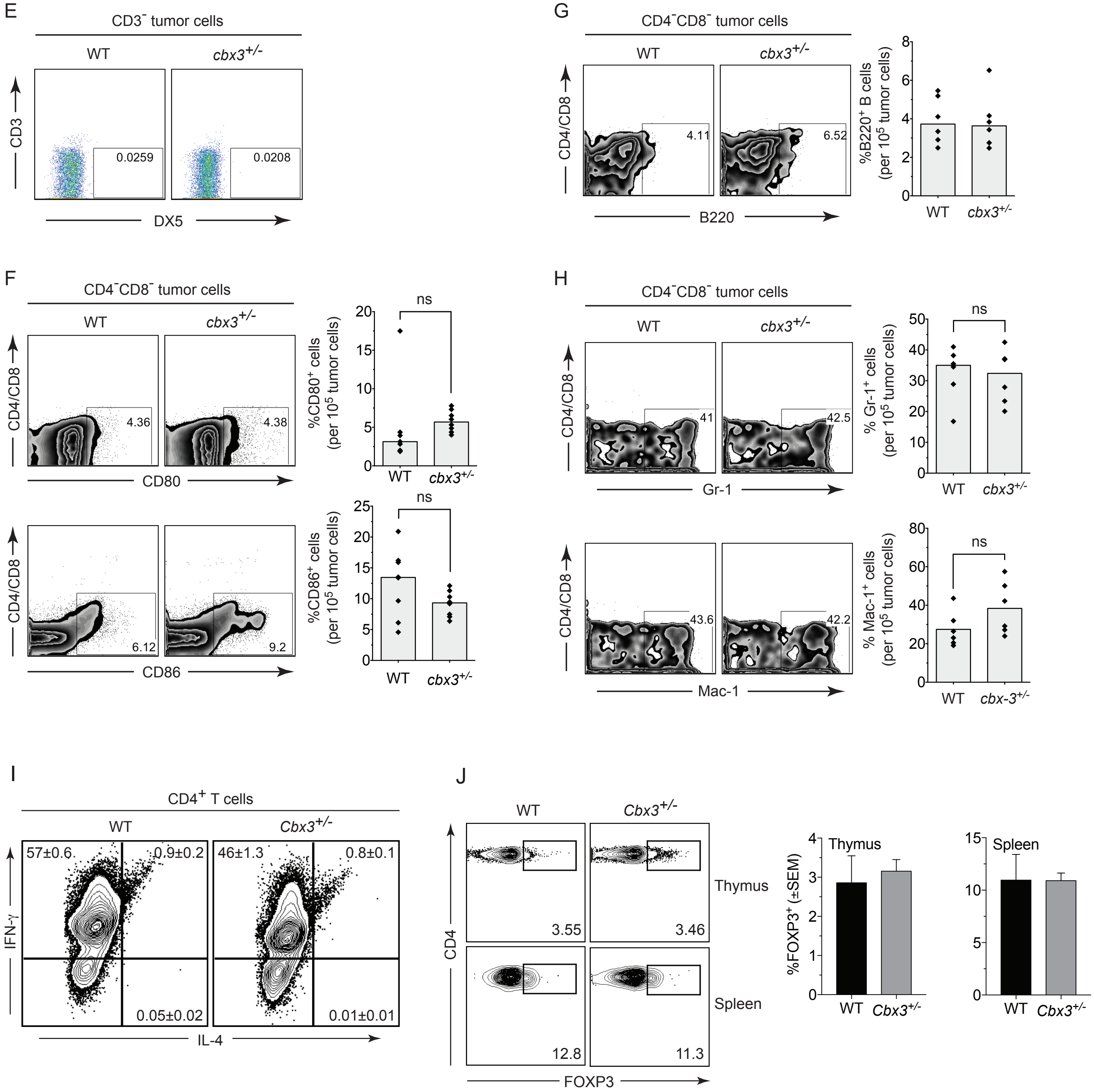

E
CD3⁻ tumor cells
WT
cbx3+/-
CD3
DX5
0.0259
0.0208
F
CD4⁻CD8⁻ tumor cells
WT
cbx3+/-
CD4/CD8
CD80
4.36
4.38
%CD80+ cells (per 10^5 tumor cells)
ns
CD86
6.12
9.2
%CD86+ cells (per 10^5 tumor cells)
G
CD4⁻CD8⁻ tumor cells
B220
4.11
6.52
%B220+ B cells (per 10^5 tumor cells)
H
CD4⁻CD8⁻ tumor cells
Gr-1
41
42.5
% Gr-1+ cells (per 10^5 tumor cells)
Mac-1
43.6
42.2
% Mac-1+ cells (per 10^5 tumor cells)
cbx-3+/-
I
CD4+ T cells
WT
Cbx3+/-
IFN-γ
IL-4
57±0.6
0.9±0.2
0.05±0.02
46±1.3
0.8±0.1
0.01±0.01
J
CD4
FOXP3
Thymus
Spleen
3.55
3.46
12.8
11.3
%FOXP3+ (±SEM)

Fig. S5

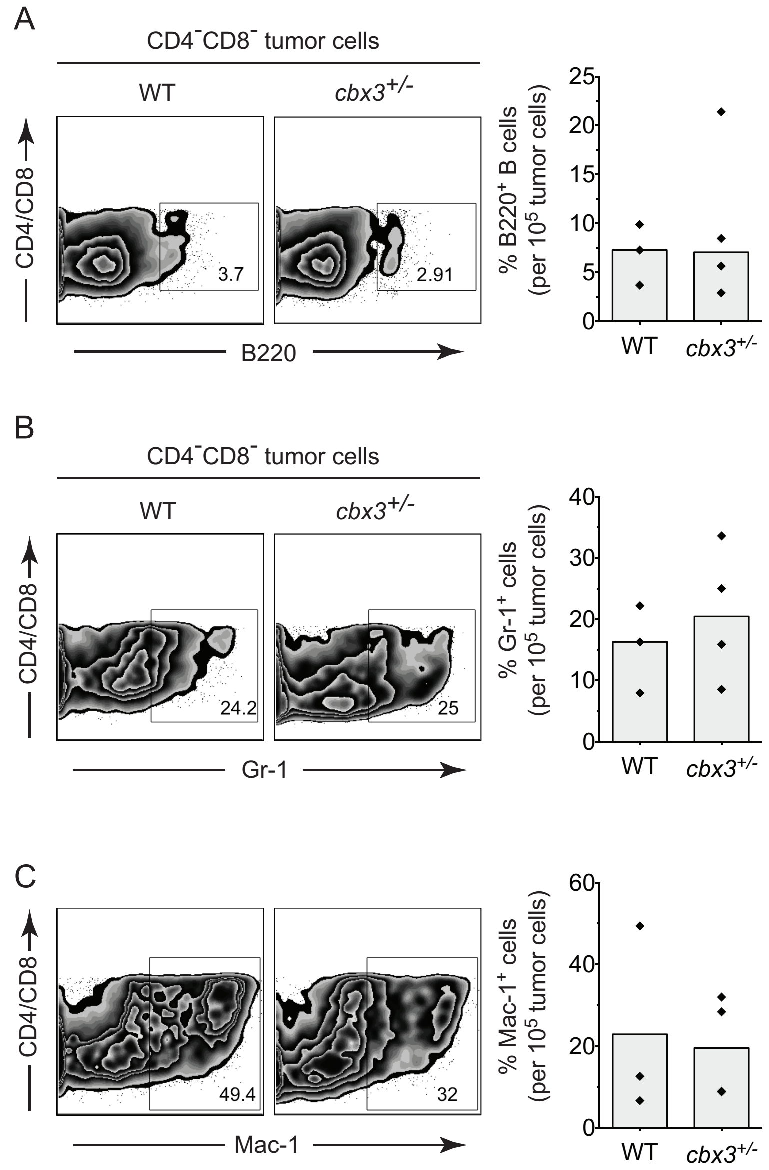

A
CD4-CD8- tumor cells
WT
cbx3+/-
CD4/CD8
B220
3.7
2.91
% B220+ B cells (per 10^5 tumor cells)
25
20
15
10
5
0
WT
cbx3+/-
B
CD4-CD8- tumor cells
WT
cbx3+/-
CD4/CD8
Gr-1
24.2
25
% Gr-1+ cells (per 10^5 tumor cells)
40
30
20
10
0
WT
cbx3+/-
C
CD4/CD8
Mac-1
49.4
32
% Mac-1+ cells (per 10^5 tumor cells)
60
40
20
0
WT
cbx3+/-

Fig. S6

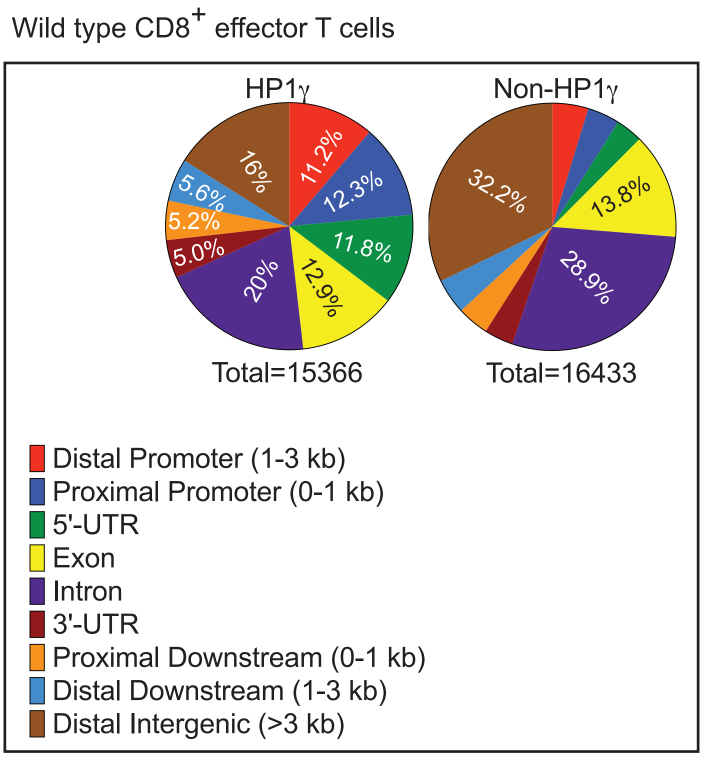

Wild type CD8+ effector T cells
HP1γ
Non-HP1γ
11.2%
12.3%
11.8%
12.9%
20%
5.0%
5.2%
5.6%
16%
Total=15366
32.2%
13.8%
28.9%
Total=16433
Distal Promoter (1-3 kb)
Proximal Promoter (0-1 kb)
5'-UTR
Exon
Intron
3'-UTR
Proximal Downstream (0-1 kb)
Distal Downstream (1-3 kb)
Distal Intergenic (>3 kb)

Fig. S7

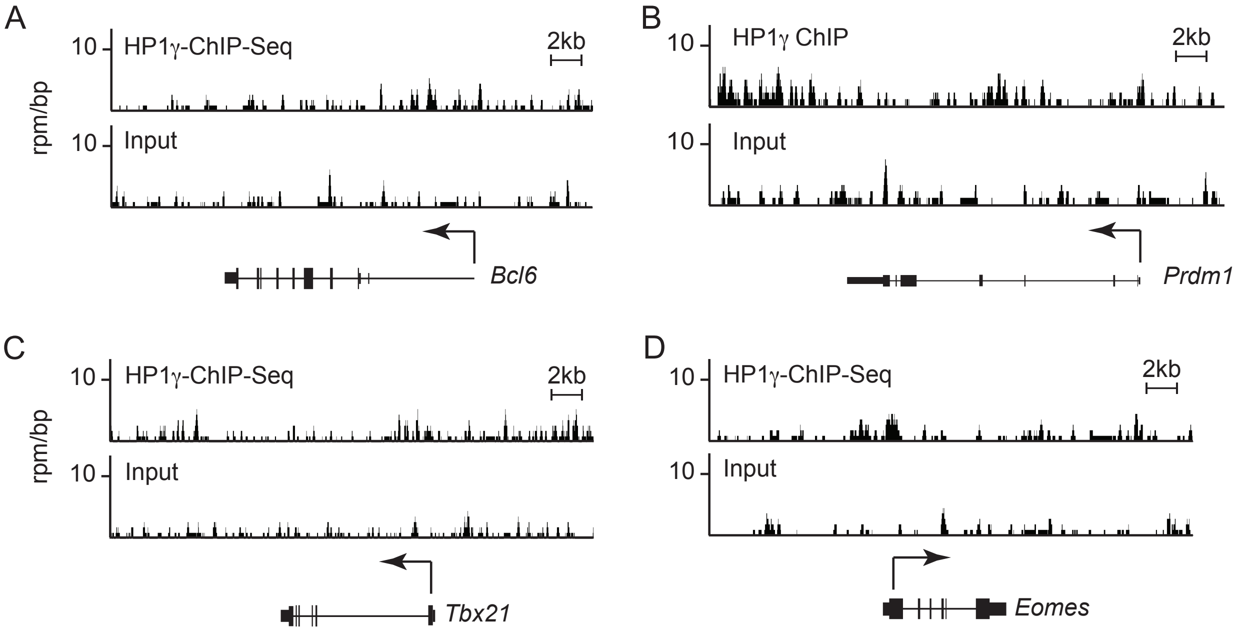

A
10
HP1γ-ChIP-Seq
2kb
rpm/bp
10
Input
Bcl6
B
10
HP1γ ChIP
2kb
10
Input
Prdm1
C
10
HP1γ-ChIP-Seq
2kb
rpm/bp
10
Input
Tbx21
D
10
HP1γ-ChIP-Seq
2kb
10
Input
Eomes

Fig. S8

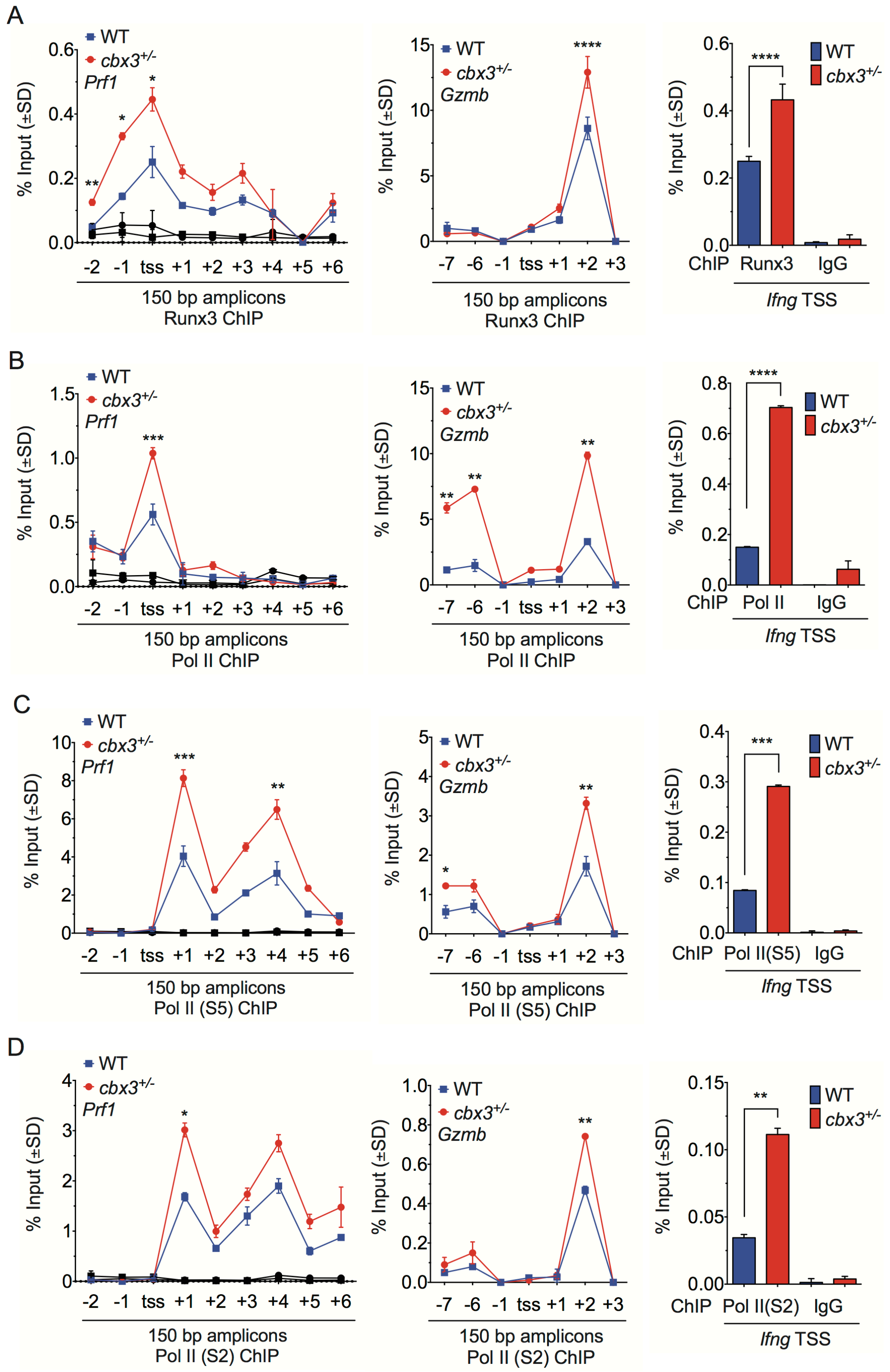

A
WT
cbx3+/-
Prf1
% Input (±SD)
-2 -1 tss +1 +2 +3 +4 +5 +6
150 bp amplicons
Runx3 ChIP
Gzmb
-7 -6 -1 tss +1 +2 +3
150 bp amplicons
Runx3 ChIP
ChIP Runx3 IgG
Ifng TSS
B
Prf1
150 bp amplicons
Pol II ChIP
Gzmb
150 bp amplicons
Pol II ChIP
ChIP Pol II IgG
Ifng TSS
C
Prf1
150 bp amplicons
Pol II (S5) ChIP
Gzmb
150 bp amplicons
Pol II (S5) ChIP
ChIP Pol II(S5) IgG
Ifng TSS
D
Prf1
150 bp amplicons
Pol II (S2) ChIP
Gzmb
150 bp amplicons
Pol II (S2) ChIP
ChIP Pol II(S2) IgG
Ifng TSS

Table S1. ChIP-Seq analysis. List of target genes bound by *Cbx3*/HP1γ in wild type mouse day-5 activated and IL-2-conditioned $CD8^+$ T cells

Chr	Start	End	Peak Summit	Gene List	Dist to Start	Position
1	13,588,200	13,595,399	13,593,456	Tram1	-3592	up
1	26,685,600	26,688,399	26,687,520	4931408C20Rik	-60	up
1	36,250,400	36,277,999	36,264,416	Uggt1, Neurl3	-20114, 9009	up, down
1	36,280,600	36,283,599	36,282,155	Neurl3	-8730	up
1	36,288,000	36,300,799	36,296,736	Arid5a	-10997	up
1	36,306,200	36,326,399	36,307,808	Arid5a, Kansl3	75, 61373	in gene, down
1	37,612,400	37,617,999	37,614,304	2010300C02Rik	105507	in gene
1	39,709,000	39,715,799	39,713,824	Rfx8	7165	in gene
1	51,466,600	51,480,799	51,478,496	Nabp1	-97	up
1	53,769,200	53,782,399	53,772,672	Stk17b	159	in gene
1	53,832,800	53,841,199	53,838,720	Hecw2	344266	in gene
1	54,835,200	54,851,799	54,847,416	Ankrd44	67315	in gene
1	58,501,600	58,511,399	58,507,696	Orc2, Gm15834	-2777, 2548	up, in gene
1	58,651,000	58,656,599	58,652,800	Gm10068, Gm20257, Als2cr12	6207, 159, 43189	down, in gene, down
1	64,084,400	64,092,599	64,090,432	Klf7	16125	in gene
1	74,226,400	74,228,599	74,227,568	Arpc2	-8982	up
1	74,397,200	74,399,799	74,398,176	Ctdsp1, Mir26b, Vil1	6567, 3866, -11208	down, down, up
1	74,669,400	74,675,799	74,672,432	Ttll4	10678	in gene
1	80,376,200	80,387,799	80,384,832	Gm6189	353	in gene
1	80,607,600	80,615,799	80,609,216	Dock10	120233	in gene
1	80,626,200	80,644,399	80,638,320	Dock10	-7911	up
1	85,275,600	85,280,799	85,280,496	C130026I21Rik, LOC101056250	-9930, 13782	up, in gene
1	85,590,200	85,593,399	85,593,088	Sp110, Sp140	10803, -7615	in gene, up
1	85,678,200	85,687,999	85,687,584	Sp100	37534	in gene
1	86,522,400	86,529,999	86,526,080	Ptma	-656	up
1	87,861,200	87,887,599	87,867,536	Dgkd, Gm19582	14249, -13237	in gene, up
1	88,211,400	88,293,199	88,277,504	Ugt1a10, Ugt1a9, Ugt1a7c, Ugt1a6b	222093, 206725, 182503, 174247	down, down, down, down
1	88,294,200	88,310,799	88,302,400	Trpm8	-4312	up
1	88,696,000	88,703,599	88,701,355	Arl4c	-3681	up
1	105,989,800	105,995,199	105,991,232	Gm7160, Zcchc2	-956, 826	up, in gene
1	106,530,000	106,559,399	106,532,800	Bcl2, D630008O14Rik	125522, 47037	in gene, down
1	106,594,800	106,614,599	106,612,496	Bcl2	101794	in gene

1	106,701,400	106,717,399	106,712,592	Bcl2, Kdsr	1698, 47150	in gene, down
1	119,525,800	119,529,999	119,526,672	Tmem185b	518	in gene
1	125,389,200	125,392,599	125,391,264	Actr3	44463	down
1	125,430,800	125,436,599	125,435,392	Actr3	335	in gene
1	125,437,400	125,446,199	125,441,184	Actr3	-5457	up
1	127,620,600	127,628,999	127,626,352	Tmem163	51669	in gene
1	127,632,400	127,636,799	127,635,424	Tmem163	42597	in gene
1	131,133,400	131,144,599	131,142,928	Dyrk3, Eif2d	-4694, -10279	up, up
1	133,023,200	133,037,799	133,025,536	Mdm4, Pik3c2b	-188, -20476	up, up
1	138,126,800	138,142,999	138,136,480	Ptprc	-5335	up
1	139,407,800	139,414,799	139,410,304	4933436E23Rik, Zbtb41	30852, -12079	down, up
1	153,313,400	153,321,599	153,313,632	Lamc1	19154	in gene
1	155,086,000	155,091,799	155,088,800	Ier5	148	in gene
1	156,039,400	156,042,399	156,040,352	Tor1aip1, Tor1aip2	-3872, 4688	up, in gene
1	161,031,800	161,043,399	161,040,240	Zbtb37, Gas5, Mir5117, Snord47	–5981, 5074, 2887, 2148	up, down, down, down, down
1	161,796,200	161,802,799	161,799,904	Fasl	-11409	up
1	164,067,000	164,079,999	164,074,128	Sele, Sell	25894, 12052	down, in gene
1	165,192,600	165,195,999	165,195,584	Sft2d2	-1151	up
1	165,704,000	165,712,199	165,710,298	Rcsd1	-10562	up
1	171,039,800	171,045,399	171,043,840	Fcgr3	15563	down
1	171,627,200	171,633,599	171,627,872	Slamf7	25165	down
1	171,645,400	171,652,799	171,648,960	Slamf7	-8851	up
1	172,508,600	172,521,799	172,518,208	Igsf9, Tagln2, Ccdc19	35995, 17962, -2922	down, down, up
1	180,848,000	180,853,199	180,851,280	Sde2	129	in gene
1	182,101,800	182,116,599	182,108,064	Srp9	-16673	up
1	183,264,000	183,268,799	183,266,128	Brox	30880	down
1	183,298,000	183,300,399	183,299,328	Brox, Aida	-2320, 2268	up, in gene
1	189,793,600	189,800,799	189,798,768	Ptpn14	88420	in gene
1	192,061,000	192,069,999	192,063,680	Traf5, Gm3934	28884, 10810	in gene, down
1	193,171,600	193,181,199	193,173,664	Irf6, A130010J15Rik, Traf3ip3	20552, 195, 27882	down, in gene, down
2	3,748,200	3,766,399	3,758,592	Fam107b	45134	in gene
2	5,378,400	5,379,799	5,379,312	Camk1d	335352	in gene
2	11,154,800	11,164,599	11,159,616	Prkcq	-12766	up
2	11,180,800	11,199,799	11,187,200	Prkcq	14818	in gene
2	11,200,600	11,212,599	11,211,936	Prkcq	39554	in gene
2	13,568,200	13,583,399	13,576,176	Gm9875, Vim	18323, 1865	down, in gene

2	18,688,000	18,695,399	18,690,256	Bmi1, LOC101055977, BC061194	13238, 475, -8766	down, in gene, up
2	18,961,600	18,973,999	18,972,768	Pip4k2a	25353	in gene
2	22,793,000	22,800,199	22,795,552	Apbb1ip	21225	in gene
2	22,804,000	22,811,999	22,807,200	Apbb1ip	32873	in gene
2	25,222,800	25,226,199	25,224,312	4933433C11Rik, Fam166a, Tubb4b, Slc34a3	−9682, 5567, 390, 9922	up, down, in gene, down
2	26,667,000	26,677,199	26,675,056	Lcn4	-3774	up
2	29,454,200	29,459,399	29,458,624	Med27	111788	in gene
2	29,460,200	29,463,799	29,461,856	Med27	115020	in gene
2	30,162,000	30,167,999	30,166,832	Tbc1d13, Endog, D2Wsu81e	32961, -4692, 11627	down, up, down
2	30,804,400	30,811,599	30,808,160	1700001O22Rik, Ntmt1	-4496, 183	up, in gene
2	31,060,600	31,065,599	31,062,240	Fnbp1	79768	in gene
2	31,113,400	31,120,199	31,116,800	Fnbp1	25208	in gene
2	32,056,800	32,089,599	32,076,016	Nup214, Fam78a, Ppapdc3	101566, 7689, -19635	down, in gene, up
2	32,160,800	32,167,599	32,162,144	Prrc2b	10996	in gene
2	32,445,800	32,452,999	32,451,024	Slc25a25, Naif1	446, 567	in gene, in gene
2	32,536,600	32,539,799	32,537,664	Fam102a	2305	in gene
2	32,604,800	32,608,599	32,607,744	St6galnac4, St6galnac6	20254, 8035	down, in gene
2	32,718,600	32,730,399	32,725,760	Cdk9, Mir2861, Mir3960, Sh2d3c	−12976, −12872,−12788, 4705	up, up, up, in gene
2	34,968,000	34,978,799	34,977,856	Traf1, Hc	-16084, 83585	up, down
2	35,038,600	35,042,199	35,041,072	Hc	20369	in gene
2	52,408,600	52,411,999	52,410,432	Arl5a	14442	in gene
2	58,155,600	58,163,799	58,160,672	Cytip, Gm13546	-550, -3302	up, up
2	59,470,200	59,478,999	59,475,840	Dapl1	-8813	up
2	73,483,800	73,492,399	73,485,440	Wipf1	43970	in gene
2	75,655,400	75,661,399	75,657,120	Hnrnpa3	-2139	up
2	75,685,200	75,689,999	75,686,960	Nfe2l2	17681	in gene
2	75,701,800	75,705,799	75,705,312	Nfe2l2, E030042O20Rik	-671, 577	up, in gene
2	75,888,200	75,898,399	75,897,936	Agps	65759	in gene
2	79,252,000	79,257,599	79,255,776	Itga4	350	in gene
2	92,454,600	92,461,599	92,459,264	Slc35c1	1254	in gene
2	93,186,000	93,201,199	93,190,400	Trp53i11, Tspan18	2816, 144087	in gene, down
2	93,449,600	93,474,399	93,460,832	Cd82, Gm10804	2114, 8013	in gene, in gene
2	101,884,800	101,887,599	101,886,037	Commd9	-225	up
2	101,989,200	101,993,599	101,992,576	Ldlrad3	193801	in gene
2	105,384,800	105,391,599	105,386,400	0610012H03Rik, Rcn1	162058, 12919	down, in gene
2	118,373,200	118,376,999	118,374,091	Gpr176	-672	up

2	118,482,800	118,492,999	118,488,096	Eif2ak4, Srp14	99479, -8400	down, up
2	119,872,000	119,874,399	119,873,120	6330405D24Rik	5283	in gene
2	119,895,800	119,899,399	119,897,632	Mga	404	in gene
2	121,138,600	121,141,799	121,140,512	Lcmt2, Adal	186, -1629	in gene, up
2	122,145,000	122,168,799	122,148,016	Patl2, B2m, Trim69	38173, 329, -12684	in gene, in gene, up
2	122,678,400	122,683,199	122,681,984	Slc30a4	20679	in gene
2	127,307,400	127,325,599	127,311,851	Stard7	41622	down
2	127,441,800	127,446,399	127,444,864	Gpat2, Fahd2a	19665, -299	down, up
2	129,178,400	129,194,999	129,189,760	AI847159, Slc20a1	-9087, -9013	up, up
2	130,421,200	130,424,799	130,423,696	Pced1a, Vps16	945, -624	in gene, up
2	131,176,800	131,181,199	131,180,320	Spef1, Cenpb, Cdc25b	-5510, -308, -6628	up, up, up
2	132,245,600	132,248,799	132,247,904	Tmem230, Pcna	-116, 5276	up, down
2	132,685,800	132,693,199	132,686,832	1110034G24Rik	-3451	up
2	139,837,400	139,842,399	139,841,664	Tasp1	225121	in gene
2	152,412,400	152,416,599	152,414,944	Zcchc3, 6820408C15Rik	100, -643	in gene, up
2	152,772,600	152,797,199	152,789,904	Cox4i2, Bcl2l1	35731, 41778	down, in gene
2	156,142,800	156,146,999	156,144,352	Nfs1, Romo1, Rbm39	-166, 199, 35888	up, in gene, down
2	157,133,800	157,143,199	157,139,424	Samhd1, Rbl1	-4202, 65110	up, down
2	158,714,800	158,736,999	158,725,163	Ppp1r16b	58430	in gene
2	158,738,800	158,745,799	158,742,464	Ppp1r16b	75731	in gene
2	164,865,200	164,869,999	164,867,840	Pltp, Pcif1	-10132, -11528	up, up
2	164,905,400	164,912,599	164,910,960	Zfp335, Gm11458	790, -400	in gene, up
2	164,937,800	164,949,399	164,943,968	Mmp9	-4283	up
2	166,644,200	166,661,799	166,646,592	Prex1	67240	in gene
2	166,896,400	166,907,199	166,905,728	Arfgef2, Cse1l	100147, -368	down, up
2	167,419,200	167,424,399	167,420,245	Slc9a8	-1476	up
2	167,695,400	167,699,599	167,695,648	Cebpb, A530013C23Rik	6733, 4440	down, in gene
2	167,918,200	167,926,799	167,920,544	Ptpn1	-11783	up
2	167,934,600	167,946,199	167,943,072	Ptpn1	10745	in gene
2	168,203,000	168,210,799	168,207,536	Adnp, Dpm1	-474, 22843	up, down
2	173,042,000	173,050,799	173,046,576	Rbm38	24674	down
2	173,262,800	173,265,799	173,263,520	Pmepa1	13013	in gene
2	174,462,200	174,464,399	174,463,744	Tubb1, Atp5e, Slmo2	13049, 357, 9197	down, in gene, down
3	19,657,600	19,666,599	19,659,504	Trim55	15044	in gene
3	30,895,600	30,900,199	30,898,208	Gpr160, Phc3	42258, 71207	down, down
3	51,223,200	51,230,399	51,224,544	Ccrn4l	97	in gene

3	51,727,800	51,751,799	51,748,096	Maml3	356910	in gene
3	51,754,200	51,772,399	51,769,488	Maml3	335518	in gene
3	52,926,600	52,932,999	52,927,760	Gm20750	-94	up
3	59,079,200	59,084,799	59,082,016	Med12l	75038	in gene
3	59,086,000	59,105,199	59,098,040	Med12l, Gpr171, P2ry14	91062, 3781, 32584	in gene, in gene, down
3	59,111,800	59,121,399	59,117,536	Med12l, Gpr171, P2ry14	110558, -15715, 13088	in gene, up, in gene
3	67,581,400	67,584,199	67,582,736	Mfsd1	-32	up
3	69,002,000	69,009,199	69,003,040	Ift80, Smc4	1530, -1932, -6732, -6862	in gene, up, up, up
3	69,038,400	69,047,199	69,044,304	Smc4, Trim59	39332, 438	down, in gene
3	86,836,200	86,844,799	86,837,920	Dclk2	82964	in gene
3	87,842,400	87,855,399	87,844,160	Sh2d2a, Prcc	-2595, 41402	up, down
3	87,905,400	87,913,599	87,906,048	Hdgf, Mrpl24, Rrnad1	-273, -13496, 24147	up, up, down
3	88,509,400	88,522,999	88,521,856	Lmna, Mex3a	-18524, -10539	up, up
3	89,176,000	89,194,199	89,183,136	Clk2, Scamp3, Fam189b, Gba	18331, 5651, -89, -19792	down, down, up, up
3	89,872,000	89,874,799	89,873,264	Il6ra	39898	in gene
3	95,010,200	95,016,999	95,015,824	Pi4kb, Zfp687, 4930481B07Rik	41093, -586, 199	down, up, in gene
3	95,657,600	95,676,999	95,672,288	Mcl1, Adamtsl4	13567, 15570	down, down
3	95,880,200	95,883,799	95,882,768	Mrps21, C920021L13Rik, Gm129, BC028528	-12149, 11246, -540, 9162	up, in gene, up, down
3	96,192,000	96,198,399	96,197,072	Sv2a, Bola1	15845, 514	down, in gene
3	96,231,600	96,238,599	96,237,952	Hist2h2be, Hist2h3c2, Hist2h2aa2	16831, 1175, 2422	down, down, down
3	96,261,800	96,270,399	96,269,120	Hist2h4, Hist2h3b, Hist2h2bb	-5803, 426, -580	up, down, up
3	96,555,600	96,564,799	96,558,112	Gm15441, Txnip	8689, 155	in gene, in gene
3	97,929,200	97,939,399	97,932,352	Sec22b, Gm5544	31125, 2179	down, in gene
3	101,260,200	101,302,399	101,272,432	Cd2, Gm10355	15507, 34749	down, down
3	103,015,600	103,022,799	103,020,592	Csde1	46	in gene
3	103,913,800	103,917,799	103,914,304	Ptpn22, Rsbn1	54012, 184	down, in gene
3	104,636,400	104,645,199	104,636,784	Slc16a1	-1884	up
3	105,890,200	105,916,999	105,900,896	Adora3, I830077J02Rik	30038, 31768	in gene, down
3	106,783,600	106,790,999	106,790,480	Cd53	-331	up
3	106,815,000	106,837,599	106,831,328	Olfr266	-8771	up
3	108,725,200	108,728,799	108,726,784	Gpsm2	-4485	up
3	110,249,800	110,252,399	110,250,800	Prmt6	198	in gene
3	127,873,800	127,877,199	127,875,760	5730508B09Rik	20563	in gene
3	131,105,000	131,107,999	131,106,528	Lef1	-3943	up
3	133,383,400	133,398,999	133,395,008	Ppa2	84892	down
3	135,599,800	135,611,999	135,609,280	Nfkb1	82267	in gene

3	135,717,600	135,723,199	135,718,432	Gm9799	-8912	up
3	137,849,800	137,856,399	137,852,928	LOC101055801, H2afz	11582, -11671	down, up
3	137,857,800	137,869,599	137,861,568	LOC101055801, H2afz, Dnajb14	2942, -3031, -6107	down, up, up
3	139,204,600	139,207,799	139,205,936	Stpg2	43	in gene
3	152,914,600	152,923,599	152,915,664	St6galnac5	66543	in gene
3	157,532,200	157,535,199	157,534,208	Zranb2, Mir186	-189, -10071	up, up
4	6,440,800	6,447,399	6,445,472	Nsmaf	8799	in gene
4	11,715,000	11,727,399	11,721,840	Gem	17393	down
4	16,161,400	16,167,399	16,163,296	Ripk2, A530072M11Rik	202, -814	in gene, up
4	33,257,200	33,262,199	33,261,408	Pnrc1	-12621	up
4	40,850,800	40,855,199	40,854,768	B4galt1, Mir5123	-770, -4630	up, up
4	46,483,400	46,490,999	46,486,624	Nans	-2705	up
4	46,562,200	46,583,599	46,563,840	Coro2a	38089	in gene
4	59,254,200	59,261,599	59,254,704	LOC101055769	-5347	up
4	62,467,200	62,486,799	62,475,744	Wdr31, Bspry	-4872, -4323	up, up
4	63,845,200	63,849,999	63,847,232	Tnfsf8	14052	in gene
4	70,377,800	70,378,599	70,378,176	Cdk5rap2	32191	in gene
4	72,198,200	72,202,399	72,202,016	Tle1, C630043F03Rik	-1097, 772	up, in gene
4	88,088,000	88,096,599	88,094,320	Focad	-310	up
4	102,978,600	102,993,399	102,984,064	Sgip1, Gm12709, Tctex1d1	223701, 5691, -2315	down, in gene, up
4	106,955,200	106,961,599	106,958,528	Ssbp3	47058	in gene
4	106,968,600	106,973,999	106,970,592	Ssbp3	59122	in gene
4	108,305,600	108,320,799	108,311,040	Zyg11b, Selrc1	-9950, -17112	up, up
4	108,346,000	108,352,399	108,350,240	Selrc1	22088	down
4	120,048,800	120,057,399	120,051,040	Hivep3	236362	in gene
4	122,884,200	122,888,599	122,886,416	Cap1	-535	up
4	122,981,800	122,986,399	122,983,840	Mycl1	-12259	up
4	123,563,200	123,572,999	123,564,576	Macf1	119784	in gene
4	129,080,200	129,089,199	129,086,432	Rnf19b	28161	down
4	129,563,400	129,574,399	129,573,088	Lck, Fam167b	553, 5492	in gene, down
4	129,906,800	129,912,199	129,909,536	Gm12966, E330017L17Rik, Spocd1	10587, 3310, -11173	down, down, up
4	130,908,000	130,919,599	130,914,352	Laptm5	1018	in gene
4	132,746,000	132,750,599	132,749,077	Smpdl3b	8094	in gene
4	132,965,400	132,979,599	132,976,512	Fgr	2417	in gene
4	133,261,000	133,267,199	133,263,296	Map3k6, Sytl1, Tmem222	22478, -209, 14494	down, up, down
4	133,609,800	133,614,999	133,612,224	Sfn, Zdhhc18	-10056, 21205	up, in gene

4	133,617,200	133,632,799	133,622,688	Zdhhc18, Gm13213	10741, 15995	in gene, down
4	134,046,800	134,054,199	134,050,592	Zfp683	-603	up
4	134,091,400	134,100,999	134,098,912	Aim1l, Cd52, Ubxn11	30460, -3839, -3671	down, up, up
4	134,113,600	134,130,399	134,120,192	Ubxn11, Sh3bgrl3, Cep85	17609, 8561, 66893	in gene, down, down
4	134,360,400	134,365,599	134,365,152	Slc30a2, Extl1	22106, 7395	down, in gene
4	136,027,000	136,035,599	136,028,544	Tceb3	-6895	up
4	136,173,200	136,180,799	136,177,568	E2f2	5174	in gene
4	137,047,600	137,051,199	137,048,960	Zbtb40	-265	up
4	139,573,200	139,579,799	139,575,840	Iffo2	45292	in gene
4	143,209,600	143,217,799	143,212,864	Prdm2	-155	up
4	145,256,800	145,266,599	145,257,280	Tnfrsf1b, Tnfrsf8	-10410, 57867	up, down
4	145,303,400	145,324,599	145,322,480	Tnfrsf8	-7333	up
4	146,453,800	146,458,199	146,457,432	Gm13251	8402	in gene
4	146,480,400	146,485,199	146,480,672	Gm13241	5873	down
4	146,492,000	146,496,399	146,492,448	Gm13247	-9552	up
4	147,514,400	147,530,399	147,519,440	Gm13152, Gm16503	18754, -20778	down, up
4	148,624,000	148,628,799	148,626,160	Masp2, Tardbp	23616, 836	down, in gene
4	149,645,200	149,666,199	149,658,720	Clstn1, Pik3cd	72082, 42909	down, in gene
4	149,669,400	149,674,599	149,672,080	Pik3cd	29549	in gene
4	149,687,800	149,707,399	149,696,752	Pik3cd, Tmem201	4877, 41284	in gene, down
4	150,912,600	150,917,999	150,917,120	Park7, Tnfrsf9	-7199, -3035	up, up
4	150,919,000	150,934,799	150,924,784	Park7, Tnfrsf9	-14863, 4629	up, in gene
4	150,998,200	151,010,599	151,005,744	Uts2, Per3	8647, 38878	down, in gene
4	153,953,400	153,959,199	153,956,864	BC039966, A430005L14Rik, Dffb	-6388, -373, 18217	up, up, down
4	154,855,000	154,862,599	154,856,416	Ttc34, Mmel1	216, -13169	in gene, up
4	155,126,000	155,128,399	155,127,056	Morn1	40479	in gene
4	155,692,600	155,698,399	155,694,800	B930041F14Rik, Ssu72	458, -10015	in gene, up
4	155,884,800	155,894,399	155,892,000	Cpsf3l, Pusl1, Acap3	22433, -238, 125	down, up, in gene
4	155,990,000	155,997,399	155,992,256	B3galt6, Sdf4	422, -658	in gene, up
4	156,002,200	156,005,999	156,003,840	B3galt6, Sdf4, Tnfrsf4	-11162, 10926, -9855	up, in gene, up
4	156,007,000	156,037,199	156,008,848	Sdf4, Tnfrsf4, Gm10560, Tnfrsf18, Ttll10	15934, -4847, 14976, -17494, 41969	in gene, up, down, up, down
4	156,339,400	156,343,599	156,342,864	Vmn2r-ps159	11761	down
5	3,459,600	3,511,999	3,484,688	Cdk6	140376	in gene
5	3,571,200	3,575,799	3,574,480	Fam133b, 1700109H08Rik, Rbm48	30647, 2764, 22067	down, in gene, down
5	8,421,600	8,427,199	8,423,040	Dbf4, Slc25a40	-324, 190	up, in gene
5	9,085,200	9,091,999	9,087,056	Tmem243	-13681	up

5	32,726,200	32,730,399	32,730,272	Pisd	55354	down
5	33,933,400	33,936,999	33,935,989	Nelfa	269	in gene
5	36,084,600	36,092,799	36,089,248	Sorcs2	308891	in gene
5	65,897,800	65,924,199	65,903,200	Rhoh	39631	down
5	75,063,400	75,068,399	75,067,360	Gsx2	-8241	up
5	92,501,200	92,506,599	92,505,600	Scarb2	8	in gene
5	100,426,200	100,430,599	100,429,504	Sec31a, 5430416N02Rik	-13270, 31	up, in gene
5	100,505,200	100,510,199	100,509,792	Lin54, Cops4	-9153, -8517	up, up
5	100,581,800	100,588,799	100,585,952	Plac8	-13747	up
5	100,631,800	100,650,799	100,642,496	Coq2	31760	down
5	100,718,800	100,729,999	100,723,968	Hpse	-4285	up
5	100,868,800	100,873,399	100,872,192	Agpat9	25963	in gene
5	104,045,600	104,048,399	104,046,272	Nudt9	-739	up
5	104,582,000	104,588,199	104,582,656	Zfp33b	10914	in gene
5	105,057,000	105,065,199	105,062,848	Gbp8	-9287	up
5	105,143,000	105,151,799	105,150,528	Gbp4	-10942	up
5	105,354,000	105,359,599	105,354,944	Gbp11	-8472	up
5	105,493,200	105,503,999	105,500,320	Lrrc8b	84545	down
5	105,555,400	105,560,999	105,557,344	Lrrc8c	37873	in gene
5	105,729,400	105,733,199	105,732,400	Lrrc8d	32431	in gene
5	107,684,400	107,689,599	107,687,840	4930428O21Rik	15640	down
5	107,690,600	107,706,599	107,697,120	4930428O21Rik	6360	down
5	107,717,400	107,737,199	107,734,752	Gfi1, A430072P03Rik, Evi5	-8947, 9595, 140355	up, down, down
5	107,957,800	107,969,199	107,960,736	Fam69a	26341	in gene
5	107,976,000	107,987,399	107,986,368	Fam69a	709	in gene
5	109,548,400	109,559,599	109,556,080	Crlf2	2913	in gene
5	113,830,000	113,835,199	113,830,960	Selplg, Coro1c	-459, 77746	up, down
5	114,921,600	114,926,399	114,922,464	Oasl2, Oasl1	25530, -776	down, up
5	115,165,000	115,170,399	115,167,872	Mlec, Cabp1	-9696, 18249	up, down
5	115,626,400	115,635,199	115,632,768	Gcn1l1, 1110006O24Rik, Rab35	67505, -952, 781	down, up, in gene
5	118,046,600	118,051,399	118,048,480	Tesc	20656	in gene
5	120,578,600	120,592,799	120,584,416	Tpcn1, Iqcd	4197, -4607	in gene, up
5	120,611,000	120,622,599	120,613,168	Iqcd, 1110008J03Rik, Ddx54, Ccdc42b	24145, -579, 38, 21067	down, up, in gene, down
5	122,820,600	122,826,399	122,821,088	Anapc5	251	in gene
5	123,114,800	123,124,399	123,122,416	Tmem120b, Rhof	46141, 10213	down, in gene
5	124,011,800	124,023,399	124,021,856	Hip1r, Vps37b	48228, 10404	down, in gene

5	124,072,200	124,086,999	124,084,352	Abcb9	11446	in gene
5	124,471,800	124,484,599	124,477,312	Setd8, Rilpl2, Snrnp35, Rilpl1	37382, 923, -5843, 54079	down, in gene, up, down
5	125,388,200	125,393,799	125,389,616	Ubc	401	in gene
5	129,497,200	129,502,999	129,501,088	Sfswap	-143	up
5	130,141,000	130,146,399	130,144,096	Tpst1, Kctd7	70770, -792	down, up
5	134,180,600	134,185,999	134,183,856	Wbscr16, Gtf2ird2	-7089, -182	up, up
5	134,186,800	134,189,799	134,187,616	Gtf2ird2	3578	in gene
5	135,529,000	135,541,199	135,540,000	Hip1	5122	in gene
5	135,542,000	135,547,199	135,542,096	Hip1	3026	in gene
5	137,149,000	137,154,999	137,152,384	Muc3	14755	in gene
5	137,304,400	137,313,199	137,307,872	Ache, Ufsp1, Srrt, Trip6	19595, 13203, -198, 6369	down, down, up, down
5	137,595,400	137,601,399	137,600,976	Tfr2, Mospd3, Pcolce	31125, 64, 10428	down, in gene, down
5	138,277,800	138,282,199	138,280,352	Gal3st4, Gpc2, Stag3	-7598, -415, -157	up, up, up
5	139,291,800	139,306,999	139,304,256	Adap1	21208	in gene
5	140,606,000	140,611,399	140,609,504	Lfng, Gm10091, Ttyh3	2163, -6191, 39527	in gene, up, down
5	140,633,400	140,641,199	140,636,896	Ttyh3	12135	in gene
5	142,897,600	142,916,999	142,902,480	Fbxl18, Actb	-7242, 4244	up, down
5	146,259,000	146,261,599	146,261,152	Cdk8, Mir5105	29477, 149	in gene, down
5	146,832,000	146,841,599	146,833,184	Rpl21, Rasl11a	294, -11887	in gene, up
5	149,183,600	149,187,999	149,184,672	5730422E09Rik, Uspl1	-2636, 112	up, in gene
6	15,785,200	15,800,799	15,799,648	Mdfic	78987	in gene
6	29,747,600	29,749,599	29,748,896	Smo	13399	in gene
6	31,484,400	31,491,799	31,487,424	Mkln1	88596	in gene
6	37,867,400	37,882,799	37,873,088	Trim24	2277	in gene
6	41,543,000	41,555,199	41,546,016	Trbd1, Trbj1-1, Trbj1-2, Trbj1-3	12815, 12152, 12015, 11693	in gene, down, down, down
6	48,645,200	48,651,999	48,649,472	Gimap8	2238	in gene
6	48,673,600	48,688,399	48,686,448	Gimap9, Gimap4	10313, 1870	down, in gene
6	48,689,600	48,711,799	48,702,160	Gimap4, Gimap6, Gimap7	17582, 6084, -16461	down, in gene, up
6	48,713,800	48,753,999	48,737,744	Gimap6, Gimap7, Gimap1, Gimap5	-29500, 19123, -1303, -8453	up, down, up, up
6	48,780,800	48,799,399	48,785,632	Gimap3	-14781	up
6	54,815,200	54,823,199	54,816,288	Znrf2	-628	up
6	59,405,400	59,414,599	59,409,600	Gprin3	16690	in gene
6	71,378,600	71,382,999	71,381,856	Rmnd5a	58781	down, down
6	71,630,200	71,641,399	71,633,616	Kdm3a	-711	up
6	72,546,600	72,552,999	72,550,816	Capg	6377	in gene
6	72,952,000	72,963,199	72,962,656	Tmsb10	-3908	up

6	82,862,200	82,877,399	82,872,672	2310069B03Rik	9181	down
6	82,909,400	82,916,199	82,913,333	Sema4f	26417	in gene
6	85,450,800	85,453,799	85,452,384	Smyd5, Pradc1, Cct7	20408, -414, 879	down, up, in gene
6	86,522,600	86,528,199	86,526,816	Pcbp1, 1600020E01Rik	-645, -514	up, up
6	91,126,000	91,130,799	91,129,024	Nup210	-12198	up
6	91,681,800	91,686,799	91,682,816	Slc6a6	-1251	up
6	99,417,800	99,423,599	99,420,768	Foxp1	14577	in gene
6	103,649,000	103,649,399	103,649,088	Chl1	138212	in gene
6	108,184,800	108,189,399	108,185,552	Sumf1	31	in gene
6	108,311,200	108,336,999	108,318,496	Itpr1	105400	in gene
6	108,517,400	108,529,599	108,528,032	Itpr1	314936	in gene
6	108,658,000	108,669,999	108,667,888	0610040F04Rik, Bhlhe40	-6954, 7259	up, down
6	114,891,800	114,910,999	114,898,440	Vgll4, 4631423B10Rik	23312, 6706	in gene, down
6	115,670,800	115,681,599	115,677,040	Raf1	-405	up
6	119,394,400	119,400,199	119,397,536	Adipor2	19947	in gene
6	119,503,000	119,510,599	119,508,373	Wnt5b	35974	in gene
6	120,134,800	120,139,399	120,134,944	Ninj2	41564	in gene
6	124,709,600	124,713,599	124,712,752	Lpcat3, Emg1, Phb2, Mir141, Mir200c	49648, -574, 463, 5233, 5638	down, up, in gene, down, down
6	124,802,600	124,814,799	124,806,176	Spsb2, Tpi1, Usp5	-2765, 8120, 23271	up, down, down
6	124,907,200	124,912,999	124,908,192	Lag3, Ptms, A230083G16Rik	3513, 9754, -8671	in gene, down, up
6	124,964,600	124,967,999	124,965,216	Cops7a	313	in gene
6	125,037,000	125,042,599	125,039,472	Zfp384, Ing4, Acrbp	30234, -376, -10455	down, up, up
6	125,263,000	125,268,599	125,263,152	4930417O13Rik, Tuba3a	-2373, 22890	up, down
6	127,989,200	127,997,999	127,991,936	Tspan9	151642	in gene
6	128,776,600	128,779,999	128,777,760	Klrb1c	10881	down
6	128,833,200	128,849,999	128,842,976	Klrb1b, BC035044	-16661, 48148	up, down
6	129,208,400	129,239,199	129,236,128	2310001H17Rik	2354	in gene
6	129,601,800	129,611,399	129,609,632	Klrd1	17821	down, down
6	129,677,800	129,695,199	129,680,480	Klrc1, Mir680-1, Klri1	-1570, -11055, 36652	up, up, down
6	136,513,200	136,533,399	136,518,864	Atf7ip	13	in gene
6	136,830,400	136,832,799	136,831,696	Wbp11, BC049715, Smco3	-3480, 2853, 3754	up, in gene, in gene
6	136,898,200	136,903,999	136,899,200	Erp27	22980	down
6	137,540,000	137,547,999	137,545,232	Eps8	103888	in gene
6	142,908,200	142,910,999	142,908,336	St8sia1	56116	in gene
6	144,670,200	144,680,399	144,670,560	4933425H06Rik	-2308	up
6	145,109,600	145,132,399	145,116,288	Lrmp	-5451	up

6	149,185,400	149,194,199	149,193,568	Amn1	-4856	up
6	149,303,200	149,314,399	149,307,664	2810474O19Rik	-1750	up
7	3,643,600	3,647,799	3,644,464	Prpf31, Cnot3, Mir3572	14479, -805, -11498	down, up, up
7	5,028,400	5,032,999	5,031,216	Zfp524, Zfp865, Zfp784	15708, 10840, 7230	down, in gene, down
7	13,023,200	13,027,799	13,023,392	Trim28, Chmp2a, Ube2m	-760, 11385, 14883	up, down, down
7	15,919,600	15,924,799	15,922,453	Sepw1	-82	up
7	16,307,200	16,315,199	16,309,792	Bbc3	209	in gene
7	16,841,200	16,847,399	16,842,288	Strn4, Prkd2	26399, -614	down, up
7	19,117,800	19,120,999	19,118,464	Fbxo46	-1395	up
7	19,262,200	19,272,999	19,269,152	Vasp, Ppm1n, Rtn2	2702, 10897, -13515	in gene, down, up
7	19,342,600	19,346,799	19,345,152	D830036C21Rik, Ercc1, Cd3eap	-2927, 81, 14331	up, in gene, down
7	24,096,600	24,108,999	24,105,824	Zfp180, Zfp112	23880, -6496	in gene, up
7	24,315,200	24,319,199	24,316,352	Zfp94	314	in gene
7	24,362,000	24,371,799	24,369,920	Lypd5, Kcnn4	20696, -343	down, up
7	24,886,200	24,893,999	24,890,576	Dmrtc2, Rps19, Cd79a, Arhgef1	20519, 5862, -6935, -12410	down, down, up, up
7	25,685,200	25,694,599	25,688,928	B9d2	7770	down
7	25,695,800	25,704,399	25,702,416	B9d2, Ccdc97	21258, 16637	down, down
7	25,708,400	25,721,399	25,718,912	Ccdc97, Hnrnpul1	141, 35808	down, down
7	27,978,800	27,990,999	27,989,440	Zfp780b, Gm4636	-10283, 24675	up, in gene
7	28,372,000	28,387,199	28,374,117	Plekhg2, Zfp36, Med29, Paf1	-1455, 5111, 18573, -18879	up, down, down, up
7	28,779,000	28,782,599	28,782,112	Sirt2, Gm19897, Rinl	15360, -598, -6857	in gene, up, up
7	28,808,000	28,819,199	28,812,096	Rinl, Hnrnpl, Ech1	23127, 1206, -13242	down, in gene, up
7	30,147,600	30,155,599	30,151,930	Zfp146	17797	down
7	30,317,800	30,330,199	30,325,616	Clip3, Alkbh6, Syne4, Sdhaf1	33863, 16863, 10800, -3241	down, down, down, up
7	30,999,000	31,005,399	31,002,016	Fam187b	28212	down
7	31,031,000	31,035,599	31,033,648	Fxyd5, Fxyd7	8674, 17806	in gene, down
7	31,036,400	31,042,999	31,039,008	Fxyd5, Fxyd7, Fxyd1	3314, 12446, 16648	in gene, down, down
7	31,083,600	31,092,399	31,090,240	Fxyd3, Hpn	-13543, 25050	up, down
7	31,145,000	31,163,799	31,154,848	Gramd1a	-3798	up
7	38,087,600	38,096,799	38,091,136	Ccne1	16354	down
7	38,182,800	38,192,999	38,190,592	1600014C10Rik	7375	in gene
7	45,392,000	45,396,399	45,395,296	Snrnp70, Kcna7	351, -10664	in gene, up
7	45,632,000	45,638,199	45,635,360	Izumo1, Rasip1, A030001D20Rik, Mamstr	13549, 7823, 3405, -4617	down, in gene, down, up
7	45,892,800	45,905,399	45,903,744	Kdelr1, Syngr4, Tmem143	30904, -7033, 6675	down, up, in gene
7	45,919,200	45,925,399	45,921,824	Tmem143, Emp3, Ccdc114	24755, -398, -6574	down, up, up
7	47,107,000	47,116,999	47,110,560	Ptpn5	23124	in gene

7	51,858,200	51,865,799	51,861,632	Fancf	635	in gene
7	64,284,200	64,287,799	64,287,328	Mtmr10	-342	up
7	66,113,200	66,120,199	66,114,464	Chsy1	4949	in gene
7	66,178,600	66,190,599	66,181,344	Chsy1	71829	down
7	68,270,200	68,287,999	68,278,528	Pgpep1l, Gm16157, Fam169b	-14295, -1934, 4689	up, up, in gene
7	68,347,600	68,355,799	68,351,024	Fam169b	77185	in gene
7	73,524,000	73,545,599	73,541,728	Chd2, 1810026B05Rik	18, 16667	in gene, in gene
7	73,599,200	73,608,599	73,606,112	Gm7710	12271	down
7	74,491,000	74,503,399	74,497,760	Slco3a1	57020	in gene
7	74,551,400	74,555,999	74,555,808	Slco3a1	-1028	up
7	75,607,400	75,616,599	75,610,278	Akap13	154744	in gene
7	75,773,600	75,783,399	75,775,456	AU020206	6643	in gene
7	78,713,200	78,728,599	78,722,320	E430016F16Rik	-4100	up
7	80,195,400	80,200,999	80,199,872	Sema4b	13031	in gene
7	80,259,800	80,262,199	80,261,136	Ttll13, Ngrn, Vps33b	14760, -79, -8519	down, up, up
7	80,403,800	80,426,999	80,418,160	Furin	-12729	up
7	80,571,800	80,580,799	80,575,808	Crtc3	113069	down
7	98,485,400	98,504,599	98,488,032	Gucy2d, Lrrc32	44610, -6190	down, up
7	98,528,200	98,542,399	98,534,384	A630091E08Rik	28606	down
7	98,547,200	98,588,799	98,577,312	A630091E08Rik, 2210018M11Rik	-14322, 79257	up, down
7	99,407,400	99,417,399	99,407,712	Gdpd5	26163	in gene
7	101,058,200	101,062,399	101,060,832	Gm5735	643	down
7	101,900,400	101,908,999	101,906,464	Anapc15, Tomt, Lamtor1, Lrrc51	10049, -105, 627, 27393	down, up, in gene, down
7	114,559,200	114,564,399	114,562,688	Cyp2r1	284	in gene
7	119,798,000	119,808,199	119,800,768	Eri2, 2610020H08Rik	-6710, 6638	up, in gene
7	119,911,800	119,923,199	119,912,592	Lyrm1, Dnahc3	16300, 182585	in gene, down
7	125,490,600	125,501,999	125,493,952	Nsmce1	-2410	up
7	125,625,200	125,641,399	125,634,016	Gtf3c1	73672	down
7	126,694,800	126,713,399	126,702,080	Slx1b, Bola2, Coro1a	-6297, 6080, 2674	up, down, in gene
7	127,090,200	127,104,999	127,093,136	AI467606, Qprt	1700, 28893	in gene, down
7	127,251,200	127,256,199	127,253,968	Zfp771, Dctpp1	9442, 6699	in gene, down
7	127,284,200	127,289,999	127,287,776	Gm17511, Itgal	-1309, -8484	up, up
7	127,875,200	127,885,799	127,876,960	Zfp668, Zfp646, Prss53, Vkorc1	-137, -741, 14010, 18657	up, up, down, down
7	128,371,800	128,376,599	128,372,624	Rgs10	45548	down
7	132,319,200	132,330,399	132,323,264	Chst15, Gm19463, Gm10584	-6109, -7045, 7600	up, up, down
7	135,651,000	135,656,599	135,652,544	Ptpre, 5830432E09Rik	114720, -230	in gene, up

7	139,966,400	139,977,799	139,971,280	Mir202, 6430531B16Rik, Adam8	-13520, 7475, 21208	up, down, down
7	140,370,200	140,374,999	140,374,032	Olfr530	-424	up
7	141,004,800	141,012,399	141,005,712	Ifitm3, Ifitm6	5032, 11180	down, down
7	141,326,000	141,329,999	141,327,184	Deaf1, Tmem80, Eps8l2	11599, -946, -11818	in gene, up, up
7	142,338,600	142,348,199	142,341,648	Ifitm10	30611	in gene
7	142,352,400	142,356,199	142,353,968	Ifitm10	18291	in gene
8	3,516,000	3,518,999	3,516,944	Mcoln1, Pnpla6	16425, 1560	down, in gene
8	4,675,600	4,680,199	4,677,856	Gm7461	509	in gene
8	11,631,000	11,636,599	11,635,328	Ankrd10	426	in gene
8	13,354,000	13,358,599	13,356,896	Tfdp1	17222	in gene
8	14,305,400	14,307,799	14,306,912	Dlgap2	211037	in gene
8	14,981,000	15,003,599	14,990,560	Arhgef10, Kbtbd11	78843, -20465	in gene, up
8	15,009,400	15,020,799	15,016,832	Arhgef10, Kbtbd11	105115, 5807	down, in gene
8	34,804,400	34,812,399	34,808,672	Dusp4, Gm9648	1062, 1737	in gene, down
8	34,813,600	34,821,799	34,817,328	Dusp4, Gm9648, Tnks	9718, -6919, 148362	in gene, up, down
8	35,374,600	35,381,399	35,375,840	Ppp1r3b, Gm20359	99, -820	in gene, up
8	69,897,800	69,908,199	69,902,560	Yjefn3, Ndufa13, Tssk6, Gatad2a	-8585, -2, 345, 93819	up, up, in gene, down
8	70,547,600	70,554,599	70,550,048	Ell	10373	in gene
8	70,603,600	70,613,399	70,608,314	Isyna1, Ssbp4, Lrrc25	13833, 0, -8530	down, in gene, up
8	70,691,200	70,705,999	70,699,904	Jund, Gm16486	2165, -8256	in gene, up
8	70,892,400	70,900,399	70,897,312	Slc5a5, Rpl18a, Snora68, Map1s	-4555, 131, -1456, -8662	up, in gene, up, up
8	71,375,600	71,386,599	71,376,096	Use1, Ocel1, Nr2f6, Ushbp1	9248, 4798, 5856, 19705	down, down, in gene, down
8	71,556,600	71,564,999	71,558,912	Mvb12a, Tmem221, Nxnl1, Slc27a1	15982, -41, 7737, -10015	down, up, down, up
8	71,690,000	71,697,799	71,692,720	Jak3, Insl3, B3gnt3	16337, 3468, 9080	down, down, in gene
8	71,907,200	71,909,399	71,908,608	Zfp882	2	in gene
8	72,155,600	72,177,799	72,160,800	Tpm4, Rab8a	25508, -400	down, up
8	72,443,400	72,449,999	72,443,712	Calr3, 1700030K09Rik	66, -168	in gene, up
8	72,474,600	72,480,799	72,475,200	Cherp, Slc35e1	33, 17414	in gene, down
8	81,007,800	81,017,399	81,015,328	Usp38, LOC101055650	-422, 1752	up, in gene
8	81,772,800	81,785,199	81,773,872	Inpp4b	58672	in gene
8	81,801,200	81,808,799	81,805,728	Inpp4b	90528	in gene
8	83,734,000	83,743,199	83,737,024	Cd97	4287	in gene
8	84,142,200	84,149,599	84,148,096	Podnl1, Cc2d1a, 4930432K21Rik	22107, -343, 58	down, up, in gene
8	84,974,400	84,987,999	84,985,952	Rnaseh2a, LOC101055733, Prdx2, Junb, Hook2	−19941, −16419, 16304, −7204, −4643	up, up, down, up, up
8	105,263,200	105,271,799	105,265,600	B3gnt9, Tradd, Fbxl8, Hsf4, Nol3	−10449,−1006, 952, −4201, −10847	up, up, in gene, up, up
8	107,586,800	107,591,799	107,588,262	Psmd7	220	in gene

8	111,029,600	111,035,199	111,033,547	Ddx19b, Aars	-1796, -420	up, up
8	111,542,800	111,546,399	111,543,808	Znrf1	7168	in gene
8	119,420,400	119,434,199	119,426,389	Mlycd, Osgin1	31497, -10773	down, up
8	120,713,600	120,730,799	120,716,704	Irf8	-19672	up
8	121,845,000	121,852,999	121,849,648	Gm20269	9499	down
8	122,357,200	122,364,599	122,358,976	Trhr2	1770	in gene
8	122,434,000	122,439,599	122,435,520	Cyba, Mvd, 9330133O14Rik, Gm20735	-2580, 7902, -8136, 15802	up, in gene, up, down
8	122,541,800	122,552,399	122,551,616	Piezo1	-287	up
8	122,566,800	122,573,199	122,568,032	Cdt1, Aprt, Galns	17, 8875, 43455	in gene, down, down
8	124,893,800	124,899,799	124,898,048	Gnpat, Exoc8, Sprtn, Egln1	35015, -343, 162, 51206	down, up, in gene, down
8	126,594,200	126,596,999	126,594,800	Irf2bp2	-1364	up
8	126,822,600	126,862,599	126,850,128	A630001O12Rik	10895	in gene
9	7,860,200	7,869,599	7,863,456	Birc3	9714	in gene
9	7,870,600	7,874,799	7,870,944	Birc3	2226	in gene
9	14,749,400	14,752,399	14,752,304	Piwil4, Fut4, 1700012B09Rik	-11571, -182, 18726	up, up, down
9	14,753,600	14,758,999	14,758,704	Fut4, 1700012B09Rik	-6582, 12326	up, in gene
9	15,011,800	15,023,999	15,018,592	Panx1	26886	in gene
9	20,605,600	20,610,399	20,607,392	5730577I03Rik	-83	up
9	20,867,200	20,879,599	20,868,501	A230050P20Rik, Angptl6, Ppan	-141, 11209, -19674	up, down, up
9	21,163,600	21,169,199	21,165,296	Pde4a	-418	up
9	21,283,800	21,292,599	21,287,760	Atg4d, Kri1, Cdkn2d, Ap1m2	22461, 209, 3449, 24573	down, in gene, down, down
9	21,314,400	21,328,199	21,324,448	Ap1m2, Slc44a2	-12115, 3729	up, in gene
9	21,473,400	21,480,199	21,478,016	Dnm2	53108	in gene
9	22,275,800	22,280,199	22,278,656	Zfp810	28982	in gene
9	32,717,200	32,753,799	32,717,424	Ets1	21382	in gene
9	35,121,400	35,128,199	35,124,048	St3gal4, 4930581F22Rik, Dcps	-7238, 7320, 51939	up, in gene, down
9	40,428,200	40,436,999	40,432,832	Gramd1b	22932	in gene
9	44,002,600	44,041,999	44,025,152	Gm10688, Thy1	-30183, -18232	up, up
9	44,266,200	44,269,399	44,268,384	Nlrx1, Abcg4	215, 19860	in gene, down
9	44,331,800	44,344,999	44,334,832	Dpagt1, H2afx, Hmbs, Vps11	7987, 117, 9396, 26838	down, in gene, down, down
9	44,406,400	44,413,799	44,407,232	Slc37a4, Trappc4, Rps25, Ccdc84	9056, 316, -482, 10775	down, in gene, up, down
9	44,769,400	44,776,399	44,771,632	Arcn1, Ift46	-3824, -1377	up, up
9	44,971,800	44,988,999	44,985,856	Ube4a, Cd3g, Cd3d, Cd3e	-20256, -5425, 4070, 23734	up, up, in gene, down
9	45,052,000	45,063,199	45,055,200	Mpzl2, Mpzl3	12856, 12	down, in gene
9	45,072,800	45,086,999	45,083,040	Mpzl3, Amica1	27852, 3857	down, in gene
9	45,822,000	45,830,199	45,829,376	Cep164, Bace1	-738, -9153	up, up

9	46,112,800	46,126,199	46,119,600	Sik3	106780	in gene
9	46,171,400	46,177,999	46,174,192	Sik3	161372	in gene
9	48,494,000	48,498,999	48,495,728	Rbm7, Gm5617	-398, 383	up, in gene
9	56,932,800	56,939,399	56,937,728	Snx33, Imp3	-9357, 228	up, in gene
9	57,334,800	57,341,199	57,339,968	Gm5121	-5176	up
9	57,400,000	57,406,999	57,404,288	4930430J02Rik, Ppcdc	13218, 35826	down, down
9	64,560,800	64,570,199	64,562,656	Megf11	177030	in gene
9	64,784,600	64,802,399	64,798,336	Dennd4a	-12675	up
9	65,285,800	65,297,599	65,293,008	Cilp, Clpx	27828, -1287	down, up
9	66,277,400	66,284,999	66,282,864	Dapk2	124638	down
9	66,873,800	66,876,799	66,874,080	Rab8b, Gm19279	45625, 12868	in gene, down
9	70,283,000	70,285,999	70,284,768	Myo1e	77418	in gene
9	72,093,200	72,102,799	72,096,960	Tcf12	14859	in gene
9	72,271,600	72,276,999	72,274,624	Zfp280d	-275	up
9	73,068,800	73,076,199	73,071,760	Rab27a	26895	in gene
9	75,407,400	75,419,399	75,409,264	Mapk6, 4933433G15Rik	750, -920	in gene, up
9	78,476,000	78,479,999	78,476,816	Mto1, Eef1a1	28606, 4908	down, down
9	79,917,600	79,923,599	79,919,472	Filip1	58410	in gene
9	90,242,800	90,248,599	90,245,456	Tbc1d2b	25313	in gene
9	92,249,200	92,253,799	92,250,624	Plscr1	430	in gene
9	100,543,800	100,546,999	100,545,632	Nck1, Slc35g2	421, 25453	in gene, down
9	101,069,800	101,080,399	101,070,064	Msl2	-4663	up
9	101,096,800	101,100,599	101,097,776	Msl2, Ppp2r3a	-2794, 154056	up, down
9	107,292,400	107,313,799	107,306,496	Mapkapk3, Cish	-16619, 9807	up, down
9	107,330,800	107,342,599	107,333,584	Hemk1, 6430571L13Rik	4766, -7056	in gene, up
9	108,458,600	108,463,399	108,460,240	Klhdc8b, Ccdc71	1341, -278	in gene, up
9	110,903,600	110,917,999	110,907,064	Als2cl, Gm590	26890, 9797	down, down
9	114,407,800	114,422,399	114,412,160	Tmppe, Glb1	11065, 11041	down, in gene
9	114,467,400	114,474,199	114,473,328	Glb1	72209	in gene
9	114,484,000	114,503,599	114,488,384	Glb1, Ccr4, Trim71	87265, 8160, 75985	down, down, down
9	114,637,000	114,641,599	114,640,368	Cnot10	-168	up
9	116,177,800	116,191,799	116,182,112	Tgfbr2	-6749	up
9	119,737,200	119,746,399	119,744,368	Scn11a	81088	down
9	121,669,200	121,680,199	121,674,432	Vipr1, Sec22c	31716, 30597	down, down
9	121,712,600	121,722,199	121,718,688	Sec22c, Deb1, Nktr	-13659, 8299, -493	up, down, up
9	122,856,000	122,867,599	122,866,096	Zfp445	-90	up

9	123,843,400	123,853,599	123,851,424	Fyco1, Xcr1	475, 10605	in gene, down
9	124,268,600	124,285,399	124,269,168	2010315B03Rik	43526	down
9	124,422,400	124,426,399	124,425,920	4930526I15Rik, Gm20783	2664, 14948	down, down
9	124,438,800	124,442,999	124,440,272	Gm20783	596	in gene
10	6,983,800	6,993,999	6,990,400	Oprm1, Ipcef1	201799, 32785	in gene, in gene
10	7,112,800	7,122,799	7,116,640	Cnksr3	95597	down
10	17,716,000	17,728,199	17,721,888	Cited2	-1340	up
10	19,208,200	19,218,399	19,210,101	Gm20139	68352	down
10	19,557,600	19,588,199	19,578,688	Ifngr1	-13270	up
10	21,356,200	21,367,799	21,357,760	Hbs1l, Aldh8a1	61781, -19540	in gene, up
10	21,973,400	21,986,799	21,976,320	Sgk1	94136	in gene
10	22,142,200	22,143,199	22,142,784	E030030I06Rik	6486	in gene
10	39,399,400	39,405,599	39,403,008	Fyn	33209	in gene
10	41,593,800	41,597,799	41,595,888	Ccdc162	-8135	up
10	42,838,000	42,843,199	42,839,072	Sec63	77576	down
10	42,995,600	43,007,399	42,998,960	Gm5079, Sobp	-7804, 175570	up, down
10	54,004,800	54,012,599	54,006,176	Man1a	69620	in gene
10	59,972,600	59,983,799	59,980,896	Anapc16	22216	down
10	61,294,000	61,302,199	61,297,536	Prf1	-300	up
10	62,448,400	62,451,399	62,450,024	Supv3l1, 4930507D05Rik, Vps26a	-331, 864, 36781	up, in gene, down
10	62,599,600	62,604,199	62,602,432	Ddx21	-134	up
10	66,928,000	66,932,799	66,930,976	LOC101056093	10674	down
10	67,095,800	67,101,199	67,097,376	Reep3	-388	up
10	75,561,800	75,567,399	75,566,496	Fam211b, Ggt1	-6166, -7097	up, up
10	75,585,600	75,599,599	75,592,288	Ggt1, Ggt5	18695, 2907	down, in gene
10	75,834,800	75,840,199	75,835,488	Gstt2	-607	up
10	76,011,200	76,018,199	76,016,432	Gm5134	61918	down
10	77,528,400	77,538,399	77,532,240	Itgb2	1892	in gene
10	78,242,600	78,249,599	78,244,832	Trappc10	-190	up
10	78,292,400	78,306,399	78,299,040	Agpat3	52660	in gene
10	79,979,000	79,989,999	79,988,736	Wdr18, Grin3b, Tmem259, Cnn2, Abca7	28584, 18012, -4406, 136, -8879	down, down, up, in gene, up
10	79,991,000	79,997,599	79,991,808	Tmem259, Cnn2, Abca7	-7478, 3208, -5807	up, in gene, up
10	80,022,000	80,037,399	80,024,544	Abca7, Hmha1, Polr2e	26929, 7872, 15115	down, in gene, down
10	80,114,200	80,118,399	80,115,072	Stk11	-1464	up
10	80,164,000	80,177,599	80,173,013	Midn, Cirbp, 1600002K03Rik, Efna2	24723, 5172, 69, -6469	down, down, in gene, up
10	80,397,400	80,410,599	80,403,456	Mex3d, Mbd3, Uqcr11, Tcf3	-15805, -3977, 3365, 30197	up, up, in gene, down

10	80,601,800	80,612,199	80,603,008	Adat3, Scamp4	128, 126	in gene, in gene
10	80,666,200	80,684,799	80,678,624	Btbd2, Mknk2, Mob3a	-21553, -6648, 23196	up, up, down
10	80,797,000	80,800,999	80,798,400	Dot1l, Plekhj1, Sf3a2, Amh, Jsrp1	43194, 226, -335, -6848, 15098	down, in gene, up, up, down
10	81,127,400	81,133,399	81,133,152	Map2k2, Zbtb7a	27205, -3119	down, up
10	81,176,600	81,185,799	81,178,112	Pias4, Eef2, Snord37, Dapk3, 2310050B05Rik	-10392, 1481, -849, -4895, -16579	up, in gene, up, up, up
10	81,256,600	81,268,799	81,260,832	Zfr2, Matk, Mrpl54, Apba3, Mir3057, Tjp3	27669, 3287, 6094, -7340, -10765, 30435	down, in gene, down, up, up, down
10	81,507,800	81,510,599	81,510,208	S1pr4, Gna15	-10071, 14017	up, in gene
10	82,054,800	82,061,999	82,061,056	Zfp873	12929	in gene
10	82,124,800	82,132,799	82,128,128	AU041133	115	in gene
10	82,238,800	82,243,799	82,241,248	Zfp938	27	in gene
10	83,002,400	83,007,799	83,006,784	Chst11	21287	in gene
10	88,401,400	88,413,199	88,405,920	Gnptab	26508	in gene
10	93,066,800	93,071,999	93,070,720	4930485B16Rik	10898	in gene
10	93,163,800	93,170,199	93,167,584	Cdk17, Mir1931	6708, 4799	in gene, down
10	93,183,000	93,192,999	93,191,936	Cdk17	31060	in gene
10	93,344,800	93,362,399	93,353,312	Gm17745	17080	down
10	95,295,600	95,300,999	95,298,976	Cradd	25121	in gene
10	95,322,000	95,328,399	95,323,488	Cradd, 2310039L15Rik	609, -12788	in gene, up
10	99,380,200	99,394,999	99,386,635	B530045E10Rik	87747	in gene
10	99,412,200	99,431,799	99,415,392	B530045E10Rik	116504	in gene
10	116,554,400	116,558,799	116,557,888	Cnot2	23623	in gene
10	117,826,800	117,832,399	117,829,392	Rap1b	16582	in gene
10	118,879,000	118,886,199	118,883,568	Gm10744, Ifng	86, -832	in gene, up
10	127,107,400	127,122,199	127,107,952	Os9	13208	in gene
10	127,130,800	127,147,799	127,139,443	Os9	-18283	up
10	127,183,200	127,187,399	127,186,816	Slc26a10, Arhgef25, Dtx3, F420014N23Rik, Pip4k2c	-6171, 3238, 8893, -8433, 24806	up, in gene, down, up, down
11	3,121,000	3,124,599	3,123,856	Sfi1	69607	down
11	3,127,000	3,208,399	3,182,080	Sfi1, Eif4enif1	11383, -20273	in gene, up
11	3,647,600	3,651,999	3,649,472	Tug1, Morc2a	-664, -22	up, up
11	4,266,800	4,274,599	4,269,298	Lif	11730	in gene
11	4,332,000	4,346,999	4,339,776	Hormad2	101306	down
11	4,414,000	4,420,199	4,418,336	Hormad2	22746	in gene
11	5,504,600	5,513,799	5,509,456	Xbp1	-11511	up
11	5,995,000	6,003,599	5,998,240	Camk2b	67508	in gene
11	6,518,200	6,530,999	6,525,120	Myo1g, Gm11974	-4162, 3640	up, down
11	6,708,800	6,712,399	6,709,792	Gm11981	10245	down

11	21,090,400	21,093,999	21,091,424	Peli1	100	in gene
11	34,681,600	34,686,999	34,682,016	Dock2	101807	down
11	46,073,800	46,084,199	46,076,672	Adam19	20685	in gene
11	46,317,000	46,352,399	46,326,688	Cyfip2, Itk, Gm12167	-13829, 62827, -15599	up, in gene, up
11	46,353,200	46,375,599	46,365,344	Itk, Gm12167	24171, 23057	in gene, down
11	46,379,600	46,394,599	46,389,184	Itk	331	in gene
11	46,395,600	46,401,999	46,396,192	Itk, Fam71b	-6677, -8538	up, up
11	48,787,200	48,790,799	48,787,424	Gnb2l1	-12936	up
11	48,848,200	48,859,399	48,853,728	Trim7, Gm16170, Irgm1	27590, -235, 17618	down, up, down
11	49,173,200	49,179,199	49,177,792	Btnl9	9297	in gene
11	49,975,600	49,983,999	49,979,120	Rasgef1c	77285	in gene
11	50,021,600	50,027,199	50,024,928	Rnf130	-403	up
11	50,322,800	50,327,799	50,326,000	Canx	-327	up
11	51,576,600	51,588,199	51,584,448	Col23a1, Agxt2l2	294528, -309	down, up
11	51,685,600	51,692,799	51,689,376	0610009B22Rik, Sec24a	-742, 67458	up, down
11	52,070,800	52,080,399	52,076,288	Cdkl3	72067	in gene
11	53,768,200	53,777,199	53,770,176	Irf1, Gm12216	162, 89080	in gene, down
11	54,139,000	54,142,599	54,140,576	P4ha2	39652	down
11	54,212,800	54,220,399	54,214,944	4933405E24Rik	-1656	up
11	54,962,600	54,970,799	54,965,408	Tnip1, Anxa6	-2468, 68063	up, down
11	55,076,400	55,084,999	55,079,392	Ccdc69	-1261	up
11	55,118,800	55,125,799	55,121,136	Gm2a, Gm12231, Slc36a3	23151, -2303, 30570	down, up, down
11	55,128,800	55,133,199	55,131,296	Gm12231, Slc36a3	-12463, 20410	up, in gene
11	58,100,400	58,106,399	58,103,776	Gm12248, Cnot8	494, -377	in gene, up
11	58,306,000	58,310,799	58,307,200	Zfp692, Zfp672	131, 23139	in gene, down
11	58,952,800	58,956,799	58,954,912	Hist3h2ba, Hist3h2bb-ps, Hist3h2a, Trim17	6001, -400, 227, -8869, 23138	down, up, in gene, up
11	59,883,800	59,887,599	59,885,568	Nt5m	37495	down
11	60,217,000	60,225,599	60,222,784	Srebf1, Tom1l2	-2180, 130121	up, down
11	60,726,000	60,729,799	60,728,512	Flii, Smcr7, Mir5100	-1291, 114, -151	up, in gene, up
11	60,934,200	60,938,799	60,935,744	Gm16516, Map2k3	-3877, 3687	up, in gene
11	61,646,800	61,651,399	61,648,528	Grap	-4793	up
11	62,813,000	62,818,599	62,815,424	Trim16	-4829	up
11	68,426,600	68,460,399	68,448,432	Pik3r5	16307	in gene
11	69,088,200	69,104,199	69,088,672	Vamp2, Per1	144, -10284	in gene, up
11	69,336,800	69,345,599	69,340,880	Kcnab3, A030009H04Rik, Chd3	14622, 111, 28511	down, in gene, down
11	69,661,400	69,673,999	69,666,400	Fxr2, Sox15, Mpdu1, Mir1934, Cd68, Eif4a1	33429, 11363, -3758, 3357, -338, 6023	down, down, up, down, up, down

11	69,911,200	69,918,799	69,914,432	Neurl4, Gps2, Eif5a	12554, 240, 7526	down, in gene, down
11	69,999,200	70,007,199	70,002,128	Ctdnep1, Gabarap, Phf23, Dvl2, Acadvl, Mir324	20960, 10758, 6357, 1502, 13279, -9915	down, down, down, in gene, down, up
11	70,078,000	70,084,799	70,084,656	Asgr2	-7988	up
11	70,107,200	70,121,599	70,120,992	Asgr2, Mgl2	28348, -9365	down, up
11	70,645,200	70,658,599	70,656,768	Gp1ba, Slc25a11, Rnf167, Pfn1, Eno3, Spag7	17646, -9729, 9179, -2118, -445, 12648	down, up, down, up, up, down
11	70,763,200	70,767,399	70,764,096	Zfp3	-351	up
11	73,090,400	73,097,199	73,092,224	1200014J11Rik, Itgae	44357, 1629	down, in gene
11	74,837,200	74,846,799	74,837,696	Mnt, Sgsm2	6772, 59384	in gene, down
11	75,162,600	75,174,399	75,165,456	Smg6, Hic1, Mir212, Mir132, Ovca2, Dph1	239584, 4799, -7932, -8226, 13352, 25027	down, in gene, up, up, down, down
11	79,125,400	79,139,199	79,131,952	Ksr1	14402	in gene
11	79,656,200	79,664,399	79,659,168	Rab11fip4, 4930542H20Rik	67956, 15918	in gene, down
11	83,064,800	83,071,199	83,069,792	Slfn2	4680	in gene
11	83,077,400	83,083,599	83,079,680	Slfn2	14568	down
11	83,751,800	83,754,599	83,753,536	1100001G20Rik, Heatr6	6596, -101	down, up
11	86,573,000	86,601,799	86,582,560	Tubd1, Gm9975, Vmp1	37569, -1792, 101262	down, up, down
11	86,603,000	86,614,799	86,606,368	Vmp1	77454	in gene
11	86,805,600	86,811,799	86,807,776	Dhx40	-116	up
11	87,460,000	87,464,199	87,460,608	Tex14, Rnu3b4, Rnu3b2	55543, -1678, -10760	in gene, up, up
11	87,742,000	87,765,399	87,759,424	Rnf43, Supt4a, Bzrap1	96337, 21859, 2560, -1117	down, down, up
11	88,967,800	88,972,199	88,969,024	Coil	-4911	up
11	94,063,000	94,070,399	94,065,312	Spag9	69221	in gene
11	95,412,600	95,416,399	95,413,792	Spop	-291	up
11	95,742,400	95,749,199	95,746,256	Zfp652	-2811	up
11	95,829,800	95,836,199	95,834,240	Phospho1, Abi3, Gngt2	9740, 8236, -8055	down, in gene, up
11	96,436,200	96,444,599	96,441,264	Gm11529	23283	down
11	96,986,200	96,999,999	96,996,672	Sp2	-18984	up
11	97,658,200	97,662,799	97,662,624	Mllt6	-788	up
11	97,710,000	97,724,399	97,722,496	Pcgf2, Psmb3, Pip4k2b	-21999, 19062, 22208	up, down, in gene
11	97,819,800	97,833,599	97,829,888	Lasp1, B230217C12Rik	30216, -10892	in gene, up
11	97,881,800	97,885,999	97,883,536	Fbxo47	618	in gene
11	98,468,600	98,473,399	98,470,912	Ikzf3	75119	in gene
11	98,535,400	98,542,199	98,540,512	Ikzf3, Zpbp2	5519, -10585	in gene, up
11	98,930,800	98,944,599	98,932,080	Cdc6, Rara	24191, -5616	down, up
11	99,040,000	99,043,999	99,040,811	Igfbp4	-449	up
11	99,096,600	99,112,799	99,110,720	Tns4	-21414	up
11	99,143,200	99,173,999	99,163,072	Ccr7	-7995	up

11	99,252,200	99,256,599	99,255,040	Krt222	-10973	up
11	100,837,200	100,839,799	100,837,600	Stat5b	12985	in gene
11	106,154,000	106,162,599	106,160,512	Map3k3, Limd2, Strada	75610, -370, 33091	down, up, down
11	106,270,600	106,280,599	106,273,168	Psmc5, Smarcd2, Tcam1	16983, -196, -3504	down, up, up
11	106,533,800	106,552,399	106,550,384	Tex2	62546	in gene
11	106,687,000	106,692,599	106,690,208	Pecam1	25073	in gene
11	106,777,200	106,785,599	106,779,360	Milr1, Polg2, Ddx5, Mir3064, Cep95	28092, 177, 9134, 3399, -9892	down, in gene, down, down, up
11	107,009,800	107,019,599	107,012,928	1810010H24Rik	-15295	up
11	107,411,400	107,432,199	107,417,840	Pitpnc1	52880	in gene
11	107,450,400	107,474,199	107,462,560	Pitpnc1, Psmd12	8160, -16968	in gene, up
11	108,268,000	108,277,799	108,273,664	Prkca	70224	in gene
11	109,360,000	109,363,799	109,363,552	1700096J18Rik, Gm11696, Gna13	16685, 102, 758	down, in gene, in gene
11	109,428,000	109,436,399	109,434,336	9930022D16Rik, Amz2, LOC100503496	20413, 8390, -5742	down, in gene, up
11	109,489,600	109,499,999	109,497,056	Arsg	23682	in gene
11	109,787,800	109,798,199	109,793,035	1700012B07Rik	35011	in gene
11	115,405,800	115,410,799	115,409,024	Cdr2l, Ict1, Atp5h, Kctd2	27108, 5258, 10895, -11102	down, in gene, down, up
11	116,019,400	116,026,999	116,020,736	Galk1, H3f3b, Unk	-8017, 3768, -9586	up, down, up
11	116,028,800	116,033,599	116,030,720	H3f3b, Unk	-6216, 398	up, in gene
11	116,127,600	116,131,799	116,130,672	Trim65, Mrpl38	456, 8196	in gene, down
11	116,420,200	116,426,599	116,423,776	Rnf157, Ubald2	-10744, -10318	up, up
11	116,841,800	116,848,999	116,843,456	Jmjd6, Mettl23, Srsf2, Mfsd11	-7, -59, 9638, -10559	up, up, down, up
11	117,302,000	117,312,199	117,308,416	Sept9	108755	in gene
11	117,313,000	117,343,399	117,318,304	Sept9	118643	in gene
11	117,770,600	117,774,799	117,773,056	Tnrc6c, Tmc6, Tmc8	118767, 7627, -9241	down, in gene, up
11	118,215,800	118,238,399	118,237,264	Cyth1	11328	in gene
11	118,259,200	118,267,399	118,260,384	Usp36	29860	in gene
11	120,346,600	120,352,399	120,347,456	Actg1, 0610009L18Rik, Fscn2	1028, -1222, -14078	in gene, up, up
11	120,570,000	120,584,399	120,584,032	P4hb, Arhgdia	-11096, -2412	up, up
11	120,594,200	120,601,399	120,598,608	Alyref, Anapc11, Npb, Pcyt2	-243, 187, -9869, 19282	up, in gene, up, down
11	120,809,000	120,828,199	120,827,883	Fasn, Ccdc57	-3336, 104989	up, in gene
12	3,234,000	3,240,999	3,239,184	1700012B15Rik, Rab10	3393, 70785	in gene, down
12	8,496,800	8,501,599	8,499,696	Rhob	289	in gene
12	16,806,200	16,812,799	16,811,008	Greb1, E2f6	-10122, 43	up, in gene
12	17,009,200	17,011,999	17,010,384	Pqlc3, 2410004P03Rik	-10266, 1343	up, in gene
12	20,226,800	20,234,399	20,232,544	LOC101055976	-11132	up
12	33,150,200	33,159,799	33,157,984	F730043M19Rik, Atxn7l1	-10398, 10298	up, in gene

12	51,827,200	51,834,399	51,827,440	Hectd1, Gm5785	2096, -745	in gene, up
12	51,851,400	51,871,199	51,856,832	Heatr5a	114489	down
12	51,886,000	51,890,599	51,886,240	Heatr5a	85081	in gene
12	51,970,800	51,977,599	51,972,448	Heatr5a	-1127	up
12	54,199,600	54,226,399	54,211,344	Egln3	-7470	up
12	67,056,800	67,059,799	67,059,456	Mdga2	163093	in gene
12	73,609,200	73,619,199	73,611,872	Prkch	26831	in gene
12	73,690,400	73,708,799	73,697,664	Prkch	112623	in gene
12	73,710,000	73,713,399	73,712,816	Prkch	127775	in gene
12	73,714,400	73,730,799	73,716,256	Prkch	131215	in gene
12	73,947,800	73,959,999	73,949,792	Hif1a, Gm5068, Snapc1	41925, 788, -14738	down, in gene, up
12	76,360,000	76,374,399	76,367,808	Zbtb25, Zbtb1	1752, -2458	in gene, up
12	76,884,400	76,892,799	76,887,200	Fntb	49733	in gene
12	80,642,600	80,646,199	80,644,000	Erh, Slc39a9	-139, -215	up, up
12	80,933,800	80,941,199	80,939,248	1700052I22Rik, Gm20337, Srsf5	-14801, 6151, -6256	up, down, up
12	91,297,800	91,301,399	91,299,616	Cep128	84793	in gene
12	91,584,600	91,593,599	91,590,368	Gtf2a1	119	in gene
12	100,889,000	100,894,599	100,890,016	Gpr68	18182	in gene
12	103,331,400	103,334,599	103,331,664	Asb2	24337	in gene
12	105,021,600	105,026,399	105,023,104	Snhg10, Scarna13, Mir3069, Glrx5	9175, 8245, 8037, -9585	down, down, down, up
12	105,027,400	105,033,599	105,033,120	Snhg10, Scarna13, Mir3069, Glrx5	-841, -1771, -1979, 431	up, up, up, in gene
12	107,920,800	107,928,999	107,922,928	Bcl11b	80486	in gene
12	114,597,400	114,601,799	114,599,168	Igh	1410786	in gene
13	3,534,200	3,538,999	3,537,616	Gdi2	-459	up
13	3,608,000	3,612,199	3,611,456	BC016423	-348	up
13	5,856,600	5,870,199	5,861,664	1700016G22Rik, Klf6	-3572, 175	up, in gene
13	20,085,200	20,102,399	20,092,440	Elmo1	1933	in gene
13	21,778,400	21,784,199	21,779,664	Hist1h1b, Hist1h3i, Hist1h2an, Hist1h2bp	890, 3733, 7554, -7824	down, down, down, up
13	22,040,200	22,045,199	22,040,864	Gm11292, Hist1h2ah, Hist1h2bk, Hist1h4i	14165, -5312, 4977, 488	down, up, down, in gene
13	23,540,000	23,545,199	23,542,848	Hist1h4h, Hist1h2af, Hist1h3g, Hist1h2bh	11804, 8937, 7430, 504	down, down, down, down
13	23,549,000	23,553,199	23,551,120	Hist1h2bh, Hist1h3f, LOC101056226, Hist1h4f	–7768, 7068, 2730, 523	up, down, in gene, down
13	23,554,200	23,558,799	23,555,840	Hist1h3f, LOC101056226, Hist1h4f, Hist1h1d, Hist1h3e	11788, 7450, -4197, 808, 6525	down, in gene, up, in gene, down
13	23,563,800	23,577,999	23,572,064	LOC101056226, Hist1h1d, Hist1h3e, Hist1h2ae	23674, 17032, -9699, -844	down, down, up, up
13	23,618,400	23,623,799	23,621,712	Hist1h2be, Hist1h1e	-588, 846	up, in gene
13	23,681,600	23,686,199	23,684,608	Hist1h2ac, Hist1h2bc, Hist1h1t	-649, 409, -11203	up, in gene, up

13	23,738,600	23,743,399	23,738,944	Hist1h1c, Hist1h3c, Hist1h2bb, Hist1h2ab, Hist1h3b	137, 6577, -7790, -12144, -13436	in gene, down, up, up, up
13	23,746,800	23,751,799	23,751,504	Hist1h1c, Hist1h3c, Hist1h2bb, Hist1h2ab	12697, -5983, 4770, 416	down, up, down, in gene
13	24,601,000	24,607,599	24,604,768	Fam65b	-9842	up
13	30,609,600	30,615,999	30,615,888	4930519D14Rik	845	in gene
13	30,616,800	30,620,599	30,618,144	4930519D14Rik	3101	in gene
13	30,632,000	30,636,799	30,634,720	4930519D14Rik	19677	down
13	30,811,600	30,836,399	30,823,280	Exoc2	150767	in gene
13	32,991,800	33,000,399	32,993,280	Serpinb9	-11261	up
13	35,187,400	35,190,999	35,189,312	1700011B04Rik	7689	down
13	36,284,800	36,290,999	36,288,192	Fars2	170781	in gene
13	37,654,200	37,660,199	37,658,336	AI463229	9398	in gene
13	43,171,200	43,181,999	43,177,280	Tbc1d7	-5779	up
13	43,468,200	43,477,199	43,476,512	Ranbp9	4461	in gene
13	45,620,400	45,627,799	45,623,968	Atxn1	341023	in gene
13	45,929,800	45,934,399	45,932,384	Atxn1	32607	in gene
13	51,724,600	51,746,599	51,736,064	Sema4d	57580	in gene
13	51,752,600	51,758,199	51,756,416	Sema4d, Gm19281	37228, 12949	in gene, down
13	51,759,600	51,804,599	51,776,208	Sema4d, Gm19281	17436, -6843	in gene, up
13	52,978,800	52,997,799	52,989,344	Nfil3	-8305	up
13	54,184,600	54,189,399	54,187,776	Hrh2	-4353	up
13	58,127,400	58,133,399	58,129,344	Hnrnpa0	-788	up
13	58,380,800	58,385,799	58,385,120	2210016F16Rik, Hnrnpk	105, 17396, 7766	in gene, down, down
13	60,845,200	60,849,999	60,848,368	4930486L24Rik	16048	in gene
13	63,239,000	63,259,399	63,240,192	2010111I01Rik	225258	in gene
13	63,812,600	63,818,599	63,815,184	Ercc6l2	-136	up
13	81,782,000	81,789,599	81,782,960	Cetn3	-332	up
13	93,141,000	93,145,999	93,144,800	Cmya5, Papd4	-76, 47483	up, down
13	94,151,400	94,156,799	94,153,600	Lhfpl2	95804	in gene
13	95,985,800	95,988,999	95,986,688	Sv2c	145889	in gene
13	107,018,600	107,023,999	107,022,656	Kif2a, 3830408C21Rik	-542, 86	up, in gene
13	112,393,800	112,402,399	112,396,000	Ankrd55	107549	down
13	113,159,800	113,172,199	113,168,016	Gzmk	12881	down
13	117,207,200	117,212,999	117,211,680	Emb	-8893	up
13	119,487,400	119,490,199	119,488,960	4833420G17Rik, 3110070M22Rik, Gm7120	26201, -576, 100	down, up, in gene
13	119,596,400	119,599,199	119,597,840	LOC101055764	-8810	up
13	119,601,400	119,603,199	119,601,984	LOC101055764	-4666	up

13	119,609,400	119,618,599	119,616,576	LOC101055764, Ccl28	9926, -7243	down, up
14	8,002,000	8,005,599	8,002,880	Dnase1l3, Abhd6	-8698, -22	up, up
14	10,388,600	10,407,599	10,397,072	Fhit	764960	in gene
14	14,344,800	14,358,399	14,349,360	Il3ra	2865	in gene
14	17,999,000	18,005,199	18,002,032	Thrb	341072	in gene
14	20,784,200	20,786,599	20,784,832	Camk2g	9256	in gene
14	25,513,600	25,519,799	25,517,168	Zmiz1	57983	in gene
14	27,399,600	27,414,199	27,407,221	Arhgef3	169182	down
14	27,424,400	27,432,999	27,428,608	D14Abb1e	-239	up
14	31,425,600	31,436,999	31,435,424	Sh3bp5	609	in gene
14	31,678,800	31,691,599	31,686,976	Ankrd28	143439	down
14	54,193,000	54,236,399	54,224,358	Traj35, Traj34, Traj33, Traj32, Traj31, Traj30	40584, 39659, 39000, 38257, 36463, 34492	down, down, down, down, down, down
14	56,257,600	56,266,799	56,261,032	Gzmb	1228	in gene
14	57,570,000	57,578,999	57,575,920	N6amt2, 1700039M10Rik, Xpo4	-4351, 4306, 89036	up, down, down
14	57,997,800	58,000,599	57,999,392	Micu2	-130	up
14	59,639,800	59,646,799	59,643,648	Shisa2, Atp8a2	18367, 443186	down, down
14	65,069,400	65,074,399	65,070,976	Extl3	27130	in gene
14	65,330,000	65,332,199	65,330,336	Fbxo16	63635	down
14	65,374,000	65,380,999	65,377,712	Zfp395	19036	in gene
14	66,296,400	66,299,799	66,296,896	1700001G11Rik, Trim35	233, -129	in gene, up
14	70,051,600	70,062,999	70,056,848	Pebp4	216428	in gene
14	70,271,600	70,294,199	70,276,096	Ppp3cc, Slc39a14	13353, 75328	in gene, down
14	72,747,800	72,756,999	72,755,008	LOC101055910	-16695	up
14	73,134,200	73,150,999	73,143,584	Rcbtb2	1074	in gene
14	74,946,400	74,951,399	74,948,992	Lrch1	-1115	up
14	75,175,200	75,186,999	75,178,464	Lcp1	47341	in gene
14	75,843,400	75,852,799	75,846,976	Gm4285, Tpt1, Snora31	-2012, 1720, -947	up, in gene, up
14	77,852,600	77,871,799	77,858,752	Dnajc15	16165	in gene
14	78,531,600	78,537,599	78,536,608	Akap11	252	in gene
14	78,903,200	78,917,999	78,903,872	Vwa8	54694	in gene
14	79,279,600	79,302,399	79,300,976	Rgcc	659	in gene
14	121,474,800	121,481,199	121,480,448	Slc15a1	24806	in gene
14	121,897,000	121,905,399	121,898,464	Ubac2, Gpr18	19858, 17310	in gene, down
14	121,934,000	121,965,999	121,935,744	Ubac2, Gpr183	57138, 29449	in gene, down
14	122,048,200	122,063,799	122,053,440	Timm8a2	18766	down

14	122,848,400	122,860,199	122,852,816	Pcca	318488	in gene
15	5,236,800	5,247,199	5,242,256	Ptger4	1575	in gene
15	9,460,400	9,476,399	9,469,440	Capsl	33412	down
15	9,694,800	9,703,599	9,700,864	Spef2	47942	in gene
15	12,000,000	12,017,199	12,011,392	Sub1	-15385	up
15	25,884,400	25,893,599	25,884,736	Fam134b	41472	in gene
15	27,721,000	27,727,199	27,724,288	Trio	301560	down
15	30,861,800	30,865,999	30,863,536	Ctnnd2	690943	in gene
15	39,909,600	39,914,399	39,911,360	Lrp12	32397	in gene
15	59,595,800	59,601,199	59,600,768	Nsmce2	226570	in gene
15	61,983,200	61,992,399	61,987,872	Myc	2531	in gene
15	62,162,200	62,197,199	62,179,616	Pvt1	141629	in gene
15	64,069,600	64,087,799	64,070,528	Fam49b, Asap1	-10080, 312391	up, down
15	66,793,000	66,805,799	66,801,568	Tg, Sla	130798, 30261	in gene, in gene
15	66,840,200	66,863,199	66,851,680	Tg, Sla	180910, -19851	down, up
15	73,157,400	73,164,599	73,162,880	Ago2	22067	in gene
15	75,966,200	75,972,199	75,968,944	Zfp707, Ccdc166	-241, 13341	up, down
15	76,294,400	76,302,799	76,299,968	Spatc1, Mir3079, Smpd5, Oplah	31879, -9000, 5534, 7277	down, up, down, in gene
15	76,506,400	76,516,399	76,512,341	Hsf1, Dgat1, Scrt1	34896, -523, 9788	down, up, down
15	76,922,400	76,930,999	76,925,280	Zfp647	168	in gene
15	78,448,200	78,455,799	78,450,784	Kctd17, Tmprss6	22156, 17850	down, in gene
15	78,468,800	78,488,599	78,478,272	Tmprss6, Il2rb	-9638, 16794	up, down
15	78,489,400	78,523,599	78,511,040	Il2rb, C1qtnf6	-15974, 18611	up, down
15	78,556,400	78,567,199	78,563,552	Rac2	9231	in gene
15	78,587,800	78,602,799	78,594,672	Cyth4	-2375	up
15	78,891,000	78,900,599	78,895,264	Gga1, Gm10866, Sh3bp1	18074, 4448, -4522	down, down, up
15	79,544,000	79,548,799	79,546,880	Ddx17	-139	up
15	80,074,600	80,084,999	80,083,200	Rpl3, Snord83b, Snord43, Syngr1	206, -4621, -294, -8134	in gene, up, up, up
15	80,218,200	80,223,199	80,219,296	Mgat3	45575	down
15	80,596,200	80,617,199	80,601,200	Grap2	-22283	up
15	80,650,000	80,655,399	80,652,544	Grap2	29061	down
15	84,349,600	84,355,999	84,354,704	Parvg	29984	down
15	84,386,000	84,391,399	84,389,744	1810041L15Rik	57353	in gene
15	85,249,200	85,253,799	85,251,136	Fbln1	45128	in gene
15	86,010,600	86,021,799	86,019,440	Celsr1	14337	in gene
15	96,283,200	96,289,199	96,286,368	E330033B04Rik, Arid2	-11093, -1154	up, up

15	99,152,200	99,155,599	99,153,824	Spats2	26979	in gene
15	100,666,400	100,674,999	100,669,685	Bin2, Cela1	-185, 18235	up, down
15	100,718,200	100,730,399	100,718,528	Galnt6, I730030J21Rik	10848, 14209	in gene, down
15	100,731,600	100,739,799	100,735,776	Galnt6, I730030J21Rik	-6400, -3039	up, up
15	102,403,000	102,406,199	102,405,056	Sp1	-1260	up
16	4,001,200	4,004,599	4,003,744	Slx4	-2064	up
16	4,877,400	4,883,199	4,879,296	4930562C15Rik, Gm10914, Ubald1, Mgrn1	43880, -8572, 555, -6804	down, up, in gene, up
16	10,768,200	10,792,399	10,784,480	Socs1, Tnp2, Prm3, Prm2, Prm1	1056, 4175, 6434, 7617, 12343	in gene, down, down, down, down
16	10,975,000	10,979,999	10,975,120	Litaf, Gm19955	18001, 2272	in gene, down
16	11,142,200	11,147,199	11,144,080	Txndc11, Zc3h7a	-9548, 32313	up, in gene
16	15,635,800	15,639,799	15,637,664	Mcm4, Prkdc	-264, -202	up, up
16	17,756,800	17,762,399	17,759,648	Klhl22	27	in gene
16	23,121,400	23,128,999	23,128,128	Eif4a2, Rfc4	20649, -398	down, up
16	30,384,000	30,391,199	30,389,536	Atp13a3	-1006	up
16	32,101,400	32,112,199	32,106,432	Pigx, Cep19	-6705, 6630	up, in gene
16	32,509,200	32,524,799	32,520,416	Zdhhc19	24135	down
16	32,605,800	32,611,199	32,608,688	Tfrc	-208	up
16	32,749,400	32,752,599	32,751,168	Muc4	15282	in gene
16	38,368,400	38,379,199	38,379,040	Popdc2	16867	down
16	38,400,800	38,404,599	38,403,808	Pla1a	29337	in gene
16	38,547,000	38,551,599	38,550,293	Poglut1, Tmem39a	-109, -8405	up, up
16	45,995,400	46,016,199	46,011,072	Plcxd2	-659	up
16	46,110,600	46,120,999	46,117,792	Cd96	2456	in gene
16	49,765,400	49,779,599	49,774,496	Ift57	75202	down
16	49,789,000	49,804,999	49,797,328	Gm16619	-3205	up
16	49,850,000	49,864,399	49,855,328	Cd47	-326	up
16	49,945,800	49,951,399	49,950,928	Gm5486, Gm19723	4105, -4899	in gene, up
16	55,943,800	55,953,399	55,944,288	Cep97, Gm6958	-9440, -17027	up, up
16	57,390,400	57,392,399	57,391,632	Cmss1, Filip1l	215235, 38355	in gene, in gene
16	64,756,000	64,768,199	64,763,392	4930453N24Rik	7547	down
16	78,556,200	78,564,999	78,560,256	D16Ertd472e, 4930478L05Rik	16432, -60	in gene, up
16	81,359,000	81,369,599	81,363,392	Ncam2	162695	in gene
16	91,363,000	91,368,399	91,366,784	Ifnar2	-5999	up
16	91,456,600	91,462,999	91,458,464	A930006K02Rik	-6545	up
16	93,602,600	93,607,199	93,604,144	Setd4, Cbr1	-329, -3693	up, up
16	93,775,600	93,782,399	93,778,592	Dopey2	66685	in gene

16	93,783,600	93,789,399	93,786,528	Dopey2	74621	in gene
16	93,913,000	93,919,399	93,914,304	Chaf1b, Cldn14	30403, 94533	down, down
16	94,695,200	94,702,599	94,698,048	Dyrk1a	127842	down
16	95,280,000	95,287,599	95,283,424	Kcnj15	25773	in gene
17	3,024,400	3,038,799	3,029,952	LOC101055692, Gm10232	-7530, 14781	up, down
17	5,716,200	5,720,399	5,716,896	Zdhhc14	224296	in gene
17	5,885,000	5,888,999	5,887,392	Snx9	46012	in gene
17	6,769,400	6,784,199	6,783,208	Ezr	-428	up
17	13,211,400	13,227,399	13,212,864	Gm10512, Smok2a, Smok2b	7822, -8324, -22007	down, up, up
17	13,540,200	13,553,399	13,550,752	2700054A10Rik	3342	in gene
17	21,478,200	21,480,799	21,478,640	Zfp53	-10348	up
17	21,530,800	21,538,399	21,535,632	Zfp52	93	in gene
17	24,204,800	24,207,399	24,205,760	Tbc1d24, Ntn3, 1600002H07Rik	-198, 3627, 15009	up, in gene, down
17	25,217,800	25,227,599	25,220,128	Unkl, Gnptg	31728, 19988	in gene, down
17	25,247,000	25,255,799	25,248,384	Gnptg, Tsr3, Baiap3, Ube2i	-8268, 8214, 7980, 25926	up, down, in gene, down
17	25,268,400	25,274,599	25,274,096	Ube2i, Gm17801	214, -28	in gene, up
17	27,128,800	27,134,199	27,133,568	Itpr3, Mnf1, Ip6k3	76264, 323, 34196	down, in gene, down
17	28,221,200	28,226,199	28,221,312	Def6, Ppard	13534, -11442	in gene, up
17	28,505,800	28,513,199	28,512,288	LOC100862287	5236	in gene
17	29,080,800	29,083,999	29,083,488	Trp53cor1, Cdkn1a	-4527, -7498	up, up
17	29,093,400	29,101,199	29,095,312	Cdkn1a	4326	in gene
17	29,168,600	29,173,999	29,171,392	Cpne5	66398	in gene
17	29,385,200	29,403,399	29,394,432	Fgd2	33518	down
17	29,487,400	29,515,399	29,504,064	Pim1	13019	down
17	29,710,000	29,720,999	29,716,864	Ftsjd2, Ccdc167	56263, 153	down, in gene
17	31,274,400	31,279,799	31,275,648	Rsph1	1708	in gene
17	31,820,400	31,850,999	31,836,112	Sik1	19680	down
17	33,090,800	33,093,399	33,092,480	Zfp563	3113	in gene
17	33,561,000	33,566,999	33,563,840	Adamts10, Myo1f	39644, 8133	down, in gene
17	33,947,800	33,956,399	33,951,392	H2-Ke2, Wdr46, B3galt4, Rps18, Vps52	-11049, 10669, 96, 4249, -4490	up, down, in gene, down, up
17	34,045,400	34,048,599	34,047,200	Rxrb, Col11a2	15388, 7763	down, in gene
17	34,067,400	34,072,399	34,071,680	Col11a2, H2-Pb	32243, 4454	down, down
17	34,105,000	34,125,199	34,114,464	BC051537, H2-Oa, Gm19450, Brd2	-19155, 22085, 14535, 8143	up, down, down, in gene
17	34,160,200	34,167,999	34,165,632	H2-DMb2, H2-DMb1	20216, 12441	down, down
17	34,169,000	34,178,999	34,176,096	H2-DMb1, Psmb9, Tap1	22905, 11234, -11460	down, down, up
17	34,194,200	34,217,799	34,206,025	Psmb9, Tap1, Psmb8, Tap2, Gm15821	-18695, 18469, 7830, 1546, 8434	up, down, down, in gene, down

17	34,354,200	34,355,999	34,355,616	H2-Ea-ps, Btnl2	-8069, 794	up, in gene
17	34,886,200	34,892,199	34,886,656	C2, Zbtb12, Ehmt2	-4556, -7903, -11843	up, up, up
17	34,996,400	35,006,799	34,997,877	Vars, Vwa7	-3030, -18702	up, up
17	35,182,600	35,206,999	35,191,936	Aif1, Lst1, Ltb, Tnf, Lta	-15935, -3496, -2571, 10060, 13415	up, up, up, down, down
17	35,208,000	35,226,199	35,210,208	Tnf, Lta, Nfkbil1	-8212, -4857, 25607	up, up, down
17	35,227,000	35,237,199	35,233,739	Nfkbil1, Atp6v1g2, Ddx39b	2076, -2857, -8007	in gene, up, up
17	35,249,000	35,263,599	35,259,488	Ddx39b, H2-D1	17742, -3606	down, up
17	35,761,000	35,771,399	35,766,912	4833427F10Rik	-5538	up
17	35,863,600	35,874,599	35,871,488	Mdc1, 5530401N12Rik, Nrm, Ppp1r18, Dhx16	29990, -4602, 10170, 5893, -8290	down, up, down, in gene, up
17	35,914,200	35,921,599	35,918,064	Atat1, Mrps18b, Ppp1r10, Mir1894	-7996, -1695, 868, 175	up, up, in gene, down
17	36,019,000	36,029,799	36,021,528	A930015D03Rik, H2-T24, H2-T23, H2-T22	27024, -968, 11173, 21174	in gene, up, down, down
17	39,842,200	39,850,199	39,844,288	Gm21946, Rn18s-rs5	594709, -2066	in gene, up
17	45,563,200	45,575,599	45,566,560	Nfkbie, Slc35b2, Hsp90ab1, Slc29a1	10844, 2408, 6701, 33043	down, in gene, down, down
17	47,515,000	47,532,799	47,525,296	Ccnd3	20245	in gene
17	48,285,000	48,304,599	48,293,920	Treml4, Treml2	29625, -6118	down, up
17	50,291,600	50,295,599	50,293,056	Dazl	543	in gene
17	50,519,000	50,523,399	50,522,720	Plcl2	13173	in gene
17	52,161,000	52,171,799	52,161,104	Gm19585	11814	in gene
17	56,475,800	56,480,599	56,476,672	Ptprs, Gm9258	-192, -393	up, up
17	56,608,600	56,616,999	56,616,384	Safb, 2410015M20Rik, Rpl36, Lonp1	31402, -6613, 2989, 10519	down, up, down, in gene
17	57,287,000	57,292,999	57,289,712	Vav1	10612	in gene
17	70,987,000	70,992,799	70,990,336	Myl12b, Myl12a	180, 12197	in gene, down
17	71,189,400	71,194,399	71,192,624	Lpin2	8646	in gene
17	71,483,400	71,491,399	71,485,984	Smchd1, Ndc80	-10641, 40873	up, down
17	72,920,600	72,933,399	72,932,480	Lbh	14175	in gene
17	72,937,800	72,950,199	72,940,416	Lbh	22111	in gene
17	73,812,200	73,824,199	73,814,368	Ehd3	9527	in gene
17	80,138,200	80,142,999	80,139,872	Galm	12401	in gene
17	80,371,400	80,374,799	80,373,296	Arhgef33, Gm10190	65889, 246	in gene, in gene
17	84,175,000	84,183,799	84,181,344	Zfp36l2, Thada	6603, 284864	down, down
17	86,433,000	86,440,599	86,436,144	Prkce	268359	in gene
17	86,443,400	86,448,799	86,444,736	Prkce	276951	in gene
17	87,970,800	87,980,399	87,975,136	LOC101055631, Msh6	-181, 86	up, in gene
18	4,342,600	4,354,199	4,353,152	Map3k8, 4833419F23Rik	-199, -395	up, up
18	6,240,000	6,252,399	6,241,440	Kif5b	84	in gene
18	11,926,200	11,946,399	11,933,624	Cables1	94350	in gene

18	11,947,200	11,958,999	11,954,544	Cables1	115270	down
18	23,985,200	23,990,199	23,987,008	Zfp35	-2626	up
18	24,627,400	24,633,799	24,630,240	Elp2	26279	in gene
18	31,907,200	31,913,799	31,910,576	Wdr33, Sft2d3	106519, 1327	down, in gene
18	33,037,800	33,043,199	33,041,496	Camk4	102455	in gene
18	33,212,600	33,217,399	33,214,176	Stard4	-360	up
18	38,363,400	38,374,199	38,367,808	Gm4949	-7270	up
18	38,401,000	38,414,599	38,406,592	Ndfip1	-12383	up
18	39,490,000	39,494,199	39,491,712	Nr3c1	-4467	up
18	40,308,000	40,308,399	40,308,224	Kctd16	49863	in gene
18	41,977,200	41,983,399	41,978,624	Grxcr2	20425	down
18	49,978,800	49,989,999	49,982,944	Tnfaip8	3517	in gene
18	54,894,000	54,905,799	54,902,592	Zfp608	87588	in gene
18	65,412,200	65,422,199	65,415,776	Malt1	-15221	up
18	67,209,800	67,214,599	67,210,464	Gnal, Chmp1b	122128, 5105	in gene, down
18	70,520,600	70,534,599	70,533,072	Poli	-2751	up
18	73,811,800	73,818,199	73,815,600	Me2	-208	up
18	74,267,800	74,272,199	74,267,968	Mbd1	-320	up
18	74,281,400	74,293,199	74,286,240	Mbd1, Ccdc11	17952, 3140	down, in gene
18	80,205,600	80,212,199	80,206,928	Rbfa, Txnl4a	-6309, 130	up, in gene
18	84,864,800	84,876,199	84,874,208	Cyb5	22794	in gene
19	3,762,400	3,768,799	3,767,968	Suv420h1	547	in gene
19	4,110,600	4,127,999	4,121,328	Pitpnm1, Aip, Tmem134, Cabp4	21211, 4499, -4632, 18281	down, in gene, up, down
19	4,182,200	4,195,399	4,184,992	Carns1, Tbc1d10c, Ppp1ca, Rad9a	-9513, 6055, -7182, 16611	up, in gene, up, down
19	4,503,800	4,513,599	4,510,880	2010003K11Rik, Pcx	-12297, 408	up, in gene
19	5,686,000	5,692,199	5,687,584	Pcnxl3, Map3k11	1324, -1547	in gene, up
19	5,790,400	5,804,599	5,803,392	Malat1	-721	up
19	5,838,800	5,850,399	5,844,480	Neat1, Gm9783, Frmd8	998, -1143, 30728	in gene, up, down
19	6,274,800	6,278,599	6,276,320	Mir192, Ehd1	11476, -576	down, up
19	6,396,400	6,400,799	6,400,352	Pygm, Rasgrp2	15923, -231	down, up
19	8,816,000	8,821,599	8,820,192	Ttc9c, Hnrnpul2	-898, 791	up, in gene
19	8,940,200	8,945,599	8,942,187	Eml3, Mta2, Tut1	12493, 267, -11663	down, in gene, up
19	9,974,200	9,980,199	9,975,296	Fth1, Best1	-7407, 26337	up, down
19	10,725,200	10,743,599	10,739,120	Cd5, A430093F15Rik	-146, -1827	up, up
19	18,598,400	18,603,599	18,599,424	Ostf1	32389	in gene
19	23,132,400	23,137,599	23,136,288	Klf9	-4938	up

19	23,139,800	23,142,399	23,141,178	Klf9, Mir1192	-48, -8253	up, up
19	30,029,400	30,033,199	30,030,176	Uhrf2	-337	up
19	40,872,200	40,874,999	40,873,440	E030044B06Rik	5124	in gene
19	40,893,200	40,896,999	40,894,560	Zfp518a	-145	up
19	45,649,400	45,650,999	45,650,176	Fbxw4	10017	in gene
19	46,075,200	46,079,399	46,075,664	Pprc1, Nolc1	19125, -199	down, up
19	46,302,800	46,311,199	46,305,024	4833438C02Rik, Nfkb2, Psd	605, 287, 22132	in gene, in gene, down
19	53,439,400	53,447,199	53,441,696	5830416P10Rik	23100	in gene
19	53,458,000	53,471,399	53,465,056	5830416P10Rik, LOC73899	-260, 4441	up, down
19	53,544,200	53,552,599	53,545,760	Dusp5	16442	down
19	57,095,600	57,102,199	57,099,733	Ablim1	116299	in gene
19	57,111,000	57,121,399	57,118,768	Ablim1	97264	in gene
19	61,224,200	61,228,999	61,224,416	Csf2ra, Gm4242	4002, 8519	in gene, down
X	76,598,400	76,599,199	76,599,072	4930468A15Rik	3852	in gene
X	112,370,600	112,371,199	112,370,880	Apool	59472	in gene
X	169,993,800	169,994,999	169,994,576	Mid1	309329	down
X	170,672,600	170,678,199	170,672,832	Asmt	188	in gene

up = upstream of transcription start site (TSS)
down = downstream from TSS
